# A novel pyroptosis-regulated gene signature for predicting prognosis and immunotherapy response in hepatocellular carcinoma

**DOI:** 10.3389/fmolb.2022.890215

**Published:** 2022-09-05

**Authors:** Baozhu Zhang, Zhan Wang

**Affiliations:** ^1^ Department of Radiation Oncology, People’s Hospital of Shenzhen Baoan District, The Second Affiliated Hospital of Shenzhen University, Shenzhen, China; ^2^ Department of General Surgery, People’s Hospital of Shenzhen Baoan District, The Second Affiliated Hospital of Shenzhen University, Shenzhen, China

**Keywords:** hepatocellular carcinoma, pyroptosis, prognosis, immunotherapy response, immune infiltrated cells

## Abstract

**Background:** Pyroptosis, a newly discovered type of programmed cell death, has both anti-tumor and tumor-promoting effects on carcinogenesis. In hepatocellular carcinoma (HCC), however, the associations between pyroptosis-regulated genes and prognosis, immune microenvironment, and immunotherapy response remain unclear.

Samples and methods: Sequencing data were collected from The Cancer Genome Atlas database, The International Cancer Genome Consortium (ICGC), and The Integrative Molecular Database of Hepatocellular Carcinoma (HCCDB). First, we investigated the expression levels and copy number variations (CNVs) of 56 pyroptosis genes in HCC and pan-cancer. Next, we identified 614 genes related to 56 pyroptosis-associated genes at the expression, mutation, and CNVs levels. Pathway enrichment analysis of 614 genes in the Hallmark, KEGG, and Reactome databases yielded a total of 253 significant signaling pathways. The pyroptosis-regulated genes (PRGs) comprised 108 genes that were derived from the top 20 signaling pathways, of which 57 genes had prognostic value in HCC. The least absolute shrinkage and selection operator (LASSO) analysis was performed to screen for PRGs with prognostic values. Ultimately, we constructed a risk score model with seven PRGs to predict HCC prognosis and validated its predictive value in three independent HCC cohorts. Risk scores were used to illustrate receiver operating characteristic (ROC) curves predicting 1, 3, and 5-years overall survival (OS). Single-sample gene set enrichment analysis (ssGSEA), was performed to study 28 types of immune cells infiltrated in HCC. The relationship between the risk signature and six immune checkpoint genes and immunotherapy was analyzed.

**Results:** A total of seven PRGs were obtained following multiple screening steps. The risk score model containing seven PRGs was found to correlate significantly with the HCC prognosis of the training group. In addition, we validated the risk score model in two additional HCC cohorts. The risk score significantly correlated with infiltrating immune cells (i. e. CD4^+^ T cells, etc.), ICB key molecules (i. e. HAVCR2, etc.), and ICB response.

**Conclusions:** This study demonstrated a vital role of PRGs in predicting the prognosis and immunotherapy response of HCC patients. The risk model could pave the way for drugs targeting pyroptosis and immune checkpoints in HCC.

## Introduction

Liver cancer was ranked sixth in cancer incidence and fourth in tumor-related mortality globally (Bray et al., 2018). Each year, approximately 841,000 new cases of liver cancer are diagnosed, and 782,000 people succumb to it (McGlynn et al., 2021). The majority of liver cancer diagnoses and fatalities are due to Hepatocellular carcinoma (HCC), the primary histologic type. This disease remains undetected until its advanced stage. Therefore, the global prognosis for liver cancer is poor (Golabi et al., 2017). In the carcinogenesis of HCC, many immunosuppressive cells coexist and interact with one another (Lu et al., 2019). Antibodies against PD-1 and CTLA-4 can eradicate HCC cells by stimulating T cell activation (Hida, 2018). Despite its effectiveness in treating other tumors, Immunotherapy has progressed extremely slowly for HCC.

Pyroptosis, a form of regulated cell death, is triggered by inflammatory caspases and characterized by the cleavage of pore-forming effector proteins: gasdermins (Kovacs and Miao, 2017). In the classical pathway, the inflammasomes recruit their key adapter protein, an apoptosis-associated speck-like protein with a caspase recruitment domain (ASC), to bind to procaspase-1 and activate caspase-1. Caspase-1 catalyzes the proteolytic cleavage of proIL-18/1β and gasdermin D (GSDMD). The N-terminal pore-forming domain (PFD) of GSDMD creates pores in the plasma membrane, which results in the release of IL-18/1β, the swelling of cells, and ultimately the rupturing of membranes (Kovacs and Miao, 2017). In the non-canonical method, bacterial lipopolysaccharide (LPS) activates caspase-4/5/11, resulting in pyroptosis through GSDMD cleavage (Ruan et al., 2020). Recent studies have identified a novel pyroptosis pathway dependent on the activation of caspase-3, a common substrate of apoptosis. Mature caspase-3 mediates the cleavage of gasdermin E (GSDME), separating its N-terminal PFD from its C-terminal. The N-terminal PFD of GSDME creates pores in the membrane and induces pyroptosis (Kovacs and Miao, 2017).

Pyroptosis is becoming a popular topic as it is involved in all stages of tumor progression. In stomach cancer, decreased GSDMD expression enhanced tumor growth by accelerating cell proliferation (Wang W. J. et al., 2018). Another member of the Gasdermin family, GSDME, induces pyroptosis in a caspase-3-dependent manner and acts as a tumor suppressor in stomach cancer (Wang Y. et al., 2018). Moreover, decreased GSDME levels were correlated with a shorter survival time in breast cancer (Zhang et al., 2020). Here, GSDME is considered a tumor suppressor by inducing pyroptosis and strengthening the anti-tumor effect (Zhang et al., 2020). The classical inflammasome sensor, NLRP1 can induce apoptosis and pyroptosis (Gorfu et al., 2014). NALP1 mRNA and protein levels are reported to be reduced in colorectal cancer compared to adjacent normal cells. 5-aza-2-deoxycytidine (DAC) restores the expression of NALP1 and inhibits tumor proliferation by inducing NLRP1-mediated caspase-1-dependent pyroptosis (Chen et al., 2015). When compared to healthy ovaries, GSDMD and gasdermin C (GSDMC) expression increases in serous ovarian cancer, while GSDME expression decreases (Berkel and Cacan, 2021). These studies have provided a solid theoretical basis for the relationship between cancer and pyroptosis.

As previously stated, accumulating evidence suggests that pyroptosis plays an important role in predicting prognosis and regulating the immune environment across multiple cancers. Based on a risk model consist of 15 pyroptosis-related lncRNAs, researchers found that the high-risk group displayed higher immune infiltration fraction and activity in glioblastoma (Xing et al., 2022). In a pyroptosis-derived lncRNA pair prognostics signature in gastric cancer, the high-risk group possessed lower levels of macrophage, monocytes, myeloid dendritic cells, endothelial cells, and cancer-associated fibroblasts compared with the low-risk group (Guo et al., 2022). A lncRNA signature encompassing 14 pyroptosis-related lncRNAs revealed that the high-risk group characterized an immunologically “cold” profile based on the immune cell infiltration landscape in head and neck squamous cell carcinoma (Zhu et al., 2021). However, the precise effect of pyroptosis on the tumor microenvironment (TME) and immune response in HCC is unknown. This study comprehensively examined the genetic and transcriptional changes of genes on the pyroptosis-regulated signal pathway. We further established a risk model to predict the overall survival of HCC, which showed high accuracy in both the training and validation groups. Our findings established a good predictor of prognosis, immune subtype, and immunotherapy responsiveness in patients with HCC.

## Materials and methods

### Data sources

Supplementary Figure S1 shows the workflow chart for this study. Gene expression, sample CNVs, and survival data of liver hepatocellular carcinoma (LIHC), esophageal squamous carcinoma (ESCA), lung squamous cell carcinoma (LUSC), lung adenocarcinoma (LUAD), stomach adenocarcinoma (STAD), colon adenocarcinoma (COAD) and rectum adenocarcinoma (READ) were obtained from The Cancer Genome Atlas (TCGA) website (https://xenabrowser.net/datapages/). The “CGDSR” R package was used to obtain corresponding clinical information. We used the “TCGA biolinks” R package to download the mutation data. The training group comprised 370 HCC patients with survival data from the TCGA-LIHC database. For model validation group 1, we obtained 243 HCC patients’ expression matrix and clinical information from the ICGC database (https://dcc.icgc.org/releases/current/Projects). Validation group 2 consist of two cohorts of HCC patients with expression and survival data, HCCDB6 (n = 209)and HCCDB17 (n = 94), which were obtained from the HCCDB database (http://lifeome.net/database/hccdb/home.html). All survival data and clinical parameters from the training and validation groups are summarized in Supplementary Table S1_TCGA_clin.xlsx, Supplementary Table 1_ICGC_clin.xlsx, and Supplementary Table 1_B6_17_clin, respectively. Table 1 provides the sample information.

**TABLE 1:** Sample information.

Data	Sample size	Usage
TCGA-LIHC	370 T vs. 50 N	Model building
TCGA-ESCA	151 T vs. 11 N	Characteristic description
TCGA-STAD	348 T vs. 31 N	Characteristic description
TCGA-COAD	430 T vs. 39 N	Characteristic description
TCGA-READ	154 T vs. 9 N	Characteristic description
TCGA-LUAD	497 T vs. 58 N	Characteristic description
TCGA-LUSC	489 T vs. 49 N	Characteristic description
ICGC-LIHC	243 T	Model validation
HCCDB6+ HCCDB17	303T	Model validation

### Consensus analysis of pyroptosis genes

58 pyroptosis genes were obtained from the literature (Ye et al., 2021) (PMID33828074) and the REACTOME_PYROPTOSIS gene set in the Molecular Signatures Database (MSIGDB) (https://www.gsea-msigdb.org/gsea/msigdb/) (Table 2). The TCGA-LIHC dataset has no expression data for GSDME or PJVK. Finally, we used 56 pyroptosis genes for subsequent analysis.

**TABLE 2:** Fifty-eight pyroptosis genes.

Source	Number	Gene name
PMID33828074	33	AIM2 CASP1 CASP3 CASP4 CASP5 CASP6 CASP8 CASP9 ELANE GPX4 GSDMA GSDMB GSDMC GSDMD GSDME IL18 IL1B IL6 NLRC4 NLRP1 NLRP2 NLRP3 NLRP6 NLRP7 NOD1 NOD2 PJVK PLCG1 PRKACA PYCARD SCAF11 TIRAP TNF
MSIGDB	25	APIP DHX9 DFNA5 GZMA GZMB NAIP NLRP9 ZBP1 BAK1 BAX CHMP2A CHMP2B CHMP3 CHMP4A CHMP4B CHMP4C CHMP6 CHMP7 CYCS HMGB1 IL1A IRF1 IRF2 TP53 TP63

The expression patterns, genetic variations, and survival analysis of pyroptosis genes in various cancer types

The mRNA levels of 56 pyroptosis genes were compared using the Wilcoxon test between normal liver tissue and HCC samples. The expression profiles of 56 pyroptosis genes in ESCA, LUSC, LUAD, STAD, COAD, and READ were determined. The copy number variations (CNVs) of pyroptosis genes were also examined in pan-cancer. The log-rank test was used to compare the differences in overall survival (OS) between the high and low groups based on pyroptosis gene expression.

### Screening of candidate genes

Our goal was to screen genes that regulate the 56 pyroptosis genes. First, the correlations between the 56 pyroptosis genes and the rest 19,065 non-pyroptosis genes were assessed at three levels: expression, mutation, and CNVslevels. A total of 614 genes were identified as significantly correlated genes with a threshold of | Cor | > 0.5 and an adjusted *p*-value of <0.05. Secondly, we carried out pathway enrichment analysis to find out which pathways are the 614 genes involved in. Using the tools of KEGG (Kanehisa et al., 2017), HALLMARK, and REACTOME in the metascape database (https://metascape.org/) (Zhou et al., 2019), we screened 253 pathways the 614 candidate genes enriched in. The top20 out of 253 pathways were selected as key pathways. A total of 108 genes in the top20 pathways were identified as key genes for model construction.

### Functional enrichment analysis

The “ClusterProfiler” R packages were used to perform functional enrichment analysis of candidate genes (Huang et al., 2009). The Top10 significantly enriched pathways in biological process (BP), molecular function (MF), and cellular component (CC) were identified.

### Model construction and independent verification

Survival analysis revealed that 57 out of the 108 key genes have prognostic value in HCC. The 57 key genes were subjected to Lasso regression analysis (Frost and Amos, 2017) and a risk model consisting of seven genes was constructed. The risk score was calculated as follows:

Risk score = ∑(*gene*_i_*coef_i_).

Where genei is the expression level of key genes after Lasso regression, and coefi is the weight.

This formula was used to calculate the risk scores in the training and validation groups. The optimal cut-off value was used to divide HCC patients into the high- and low-risk groups. Kaplan-Meier plotter and ROC curve were used to evaluate the prediction power of the prognostic model in one training group and three validation groups.

### Quantification of immune infiltrating cells proportions

Single sample gene set enrichment analysis (ssGSEA) is an extension of the GSEA method, which allows the definition of an enrichment score that represents the absolute degree of enrichment of the gene set in each sample within a given dataset (Charoentong et al., 2017). To determine the proportions and differences in immune cells in different risk groups, we used ssGSEA in the R package GSVA to evaluate the infiltration level of 28 types of immune cells. The detailed method has been described in previous studies (Liu et al., 2021; Guo et al., 2022). The Wilcoxon test was used to compare the differences between the high- and low-risk groups. Correlations between the risk score and the immune cell infiltration were further explored.

### Evaluation of immune checkpoint profiles

The relationship between six immune checkpoints (HAVCR2, CTLA‐4, PDCD1, PDCD1LG2, IDO1, and CD274) and risk group were examined.

### Immunotherapy assessment

Immunophenoscore (IPS) could act as a good predictor of CTLA-4 and PD-1 responsiveness as well as the response to immunotherapy. The immune checkpoint inhibitor (ICI) immunophenoscore file was obtained from the Cancer Immunome Database (TCIA, https://tcia.at/home) (Van Allen et al., 2015; Hugo et al., 2017). We then compared the responses to immunotherapy between the high- and low-risk groups.

### Statistical analysis

ROC curves and the area under curves (AUC) were computed using the “pROC” and the “timeROC” packages, respectively. Analyses between the two or more groups were performed using the Wilcox test and the Kruskal Wallis test, respectively. We used the Kaplan-Meier method for survival analysis and the log-rank test to calculate the significance of differences. All statistical analyses were performed in R 4.3.0 and SPSS16 software. All statistical *p* values are two-side and *p* < 0_·_05 represents statistical significance. In the statistical graph, ns denotes *p* ≥ 0.05, * denotes *p* < 0.05, **: denotes *p* ≤ 0.01, *** denotes *p* ≤ 0.001, **** denotes *p* ≤ 0.0001.

## Results

### Changes in the transcriptional and genetic expression of 56 pyroptosis genes in HCC and pan-cancer

**FIGURE 1:**
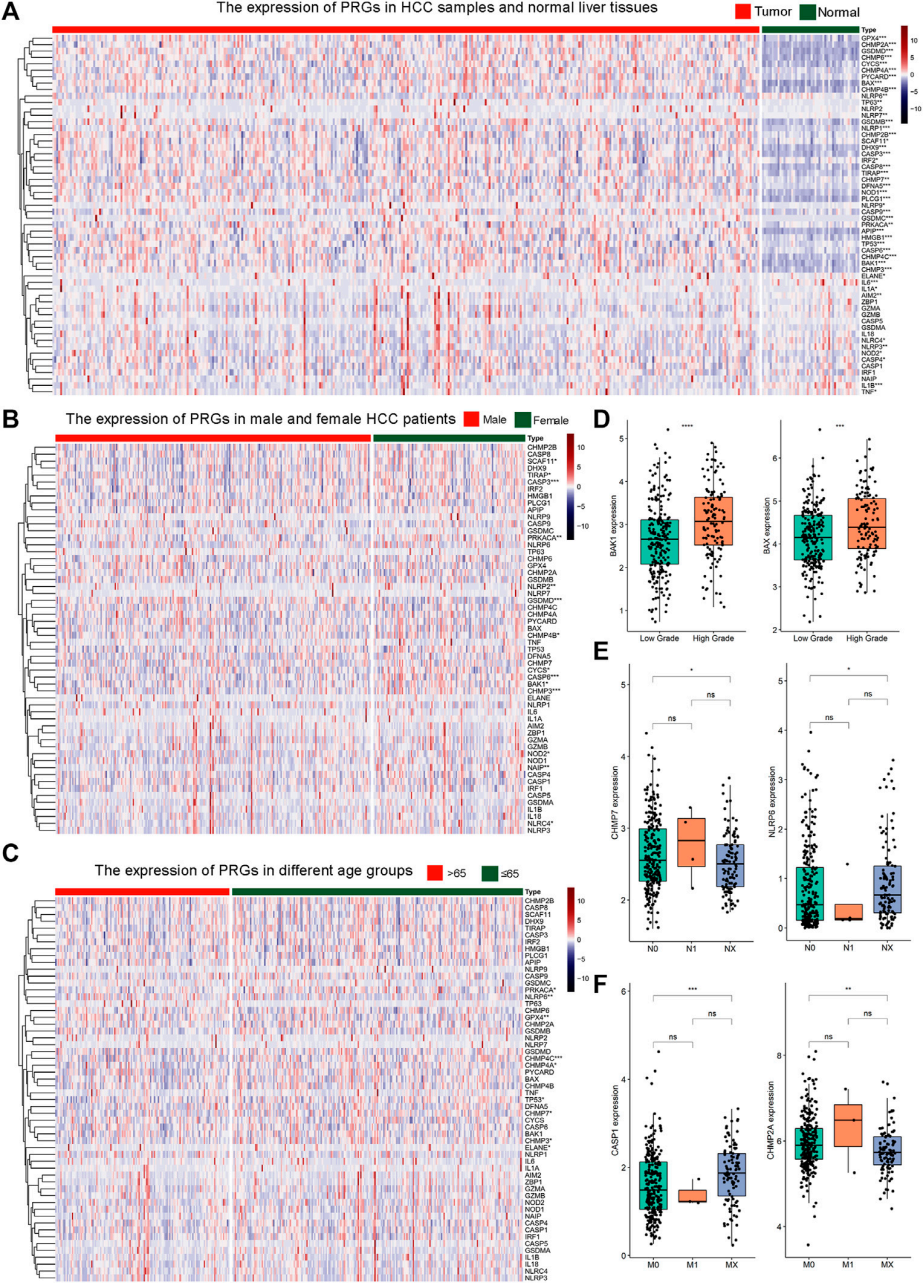
The expression profile of 56 pyroptosis genes in HCC. **(A)** Heat map illustrating the expression of 56 pyroptosis genes in HCC samples and normal liver tissues. The color from blue to red indicates a transition from low expression to high expression. * Means *p* < 0.05, ** means *p* < 0.01, ***means *p* < 0.001 (the same below).; **(B)** Heat map of the expression of 56 pyroptosis genes in male and female HCC patients; **(C)** Heat map of the expression of 56 pyroptosis genes in HCC patients of different age groups. The green color indicates patients ≤65 years old. The orange color indicates patients >65 years old. **(D)** The expression of BAK1 and BAX in HCC samples with low or high histologic grade. **(E)** The expression of CHMP7 and NLRP6 in HCC patients with N0, N1, or NX. **(F)** The expression of CASP1 and CHMP2A in HCC patients with M0, M1, or MX.

In comparison to normal controls, 46 out of the 56 (82.1%) pyroptosis genes were dysregulated in HCC. Thirty-six pyroptosis genes exhibited significantly higher transcriptional levels in HCC, including APIP, BAK1, BAX, CASP3, CASP4, CASP6, CASP8, CASP9, CHMP2A, CHMP2B, CHMP3, CHMP4A, CHMP4B, CHMP4C, CHMP6, CHMP7, CYCS, DFNA5, DHX9, GPX4, GSDMB, GSDMC, GSDMD, HMGB1, IL1A, IRF2, NLBP1, NLRP9, NOD1, NOD2, PLCG1, PRKACA, PYCARD, SCAF11, TIRAP, and TP53. In contrast, the expression levels of ten pyroptosis genes, including AIM2, ELANE, IL-1β, IL6, NLRC4, NLRP3, NLRP6, NLRP7, TNF, and TP63, were significantly lower than those of healthy controls (Figure1A). In subgroup analysis, 14 genes (SCAF11, TIRAP, CASP3, PRKACA, NLRP2, GSDMD, CHMP4B, CYCS, CASP6, BAK1, CHMP3, NOD2, NAIP, and NLRC4) exhibited significant expression differences between male and female patients (Figure1B). Nine genes (PRKACA, NLRP6, GPX4, CHMP4C, CHMP4A, TP53, CHMP7, CHMP3, and ELANE) exhibited expression differences between patients under or over 65 years of age (Figure1C). The expressions of BAK1 and BAX were significantly higher in high histologic grade HCC samples than that in low-grade HCC samples (Figure 1D, see Supplementary Figure S2 for all genes). Patients with N0 or NX had significantly different expressions of CHMP7 and NLRP6 (Figure1E, see Supplementary Figure S3 for all genes). CASP1 and CHMP2A mRNA levels were significantly different between M0 or MX patients (Figure 1F, see Supplementary Figure S4 for all genes). Our study demonstrated that the expression patterns of pyroptosis genes may be indicative of distinct traits in HCC patients.

**FIGURE 2:**
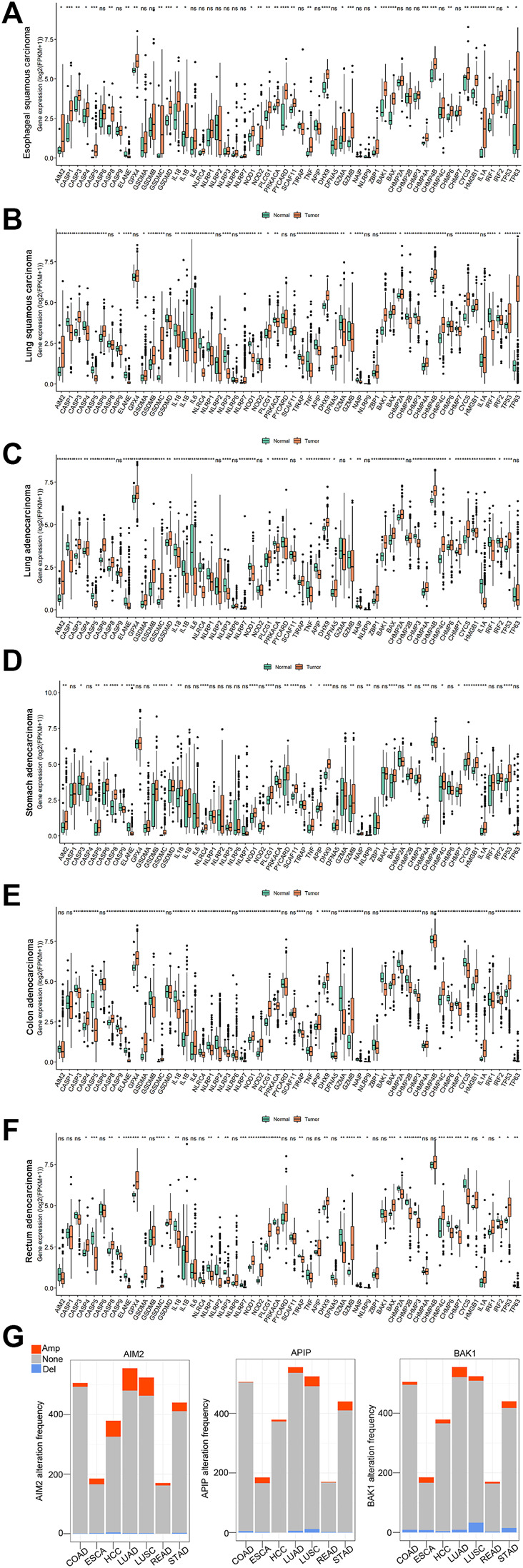
The expression and copy number variations (CNVs) of 56 pyroptosis genes in pan-cancer. **(A–F)**: The expression of 56 pyroptosis genes in esophageal squamous carcinoma (ESCA), lung squamous carcinoma (LUSC), lung adenocarcinoma (LUAD), stomach adenocarcinoma (STAD), colon adenocarcinoma (COAD) and rectum adenocarcinoma (READ). *Indicates *p* < 0.05, ** indicates *p* < 0.01, ***indicates *p* < 0.001 (the same below). **(G)**: The copy number variations of AIM2, APIP, and BAK1 in pan-cancer. The orange color indicates amplification (Amp). The blue color indicates deletion (Del). The grey color indicates no copy number variations (None).

Subsequently, we examine the expression levels of 56 pyroptosis genes between pan-cancer and their corresponding normal controls, including esophageal squamous carcinoma (ESCA), lung squamous cell carcinoma (LUSC), lung adenocarcinoma (LUAD), stomach adenocarcinoma (STAD), colon adenocarcinoma (COAD), and rectum adenocarcinoma (READ). The majority of pyroptosis genes exhibited significant dysregulation in tumor tissues compared to normal controls (Figure 2A-2F, single-gene comparison of single cancer was shown in Supplementary Figure S5–S10).

Meanwhile, we explored the copy number variations (CNVs) of pyroptosis genes in pan-cancer. The result showed the 56 pyroptosis genes had multiple CNVs in these cancers. Here, we used the CNVs modification of four genes (AIM2, APIP, BAK1, and CASP1) in pan-cancer as an example (Figure 2G, see all genes in Supplementary Figure S11).

### Prognostic value of pyroptosis genes in HCC and pan-cancer

**FIGURE 3:**
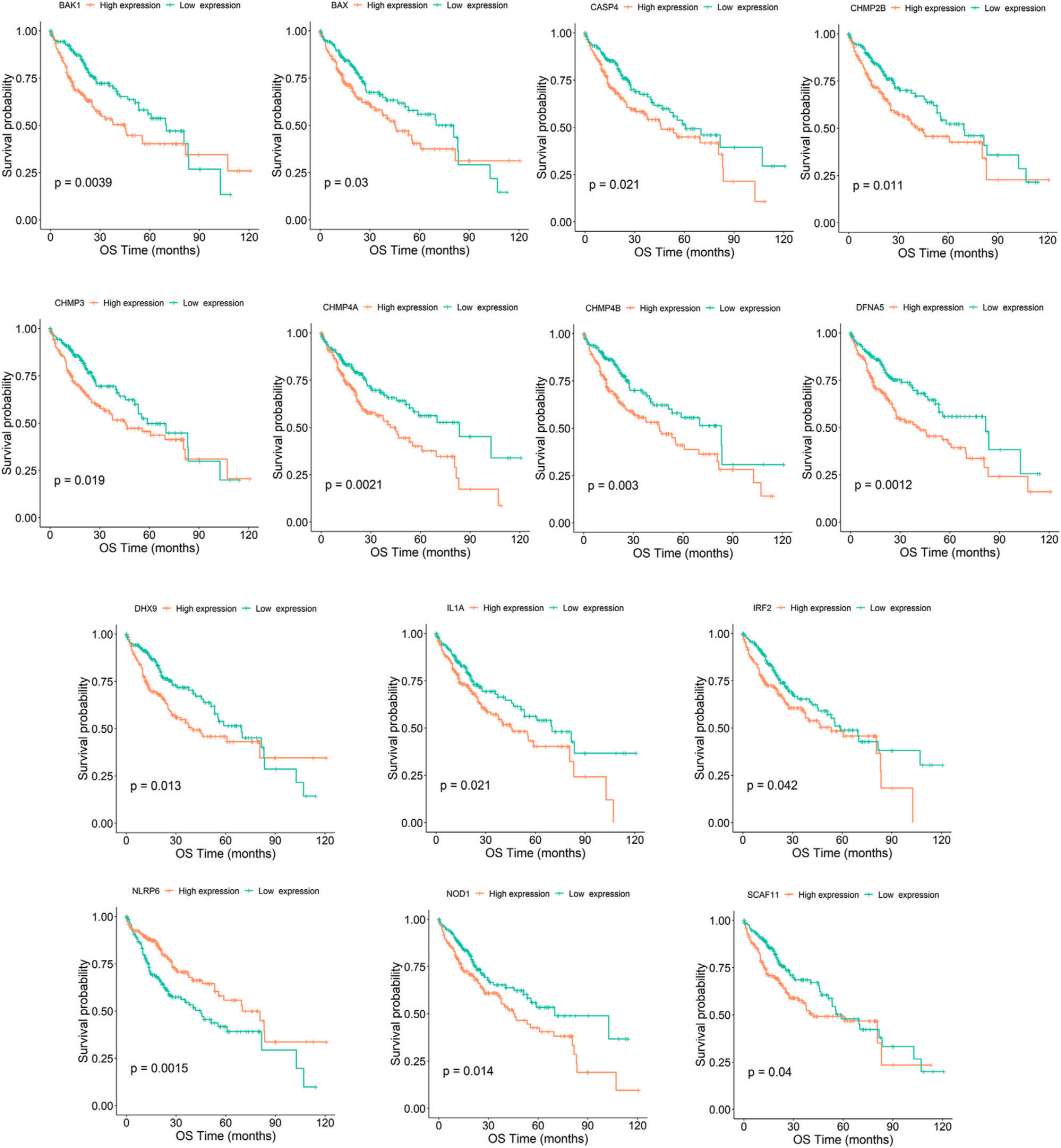
Kaplan–Meier survival curve of HCC samples with high- and low-expression of the 14 pyroptosis genes (BAK1, BAX, CASP4, CHMP2B, CHMP3, CHMP4A, CHMP4B, DFNA5, DHX9, IL-1A, IRF2, NLRP6, NOD1, and SCAF11). OS denotes overall survival.

Tumor samples were divided into high and low expression groups based on the median value of gene expression. According to the results of the survival analysis, 14 out of the 56 Pyroptosis genes were significantly associated with HCC prognosis. The high expression of BAK1, BAX, CASP4, CHMP2B, CHMP3, CHMP4A, CHMP4B, DFNA5, DHX9, IL-1A, IRF2, NLRP6, NOD1, and SCAF11 predicted poor prognosis in HCC (Figure 3, see the rest genes in Supplementary Figure S12).

### Screening of candidate genes

**FIGURE 4:**
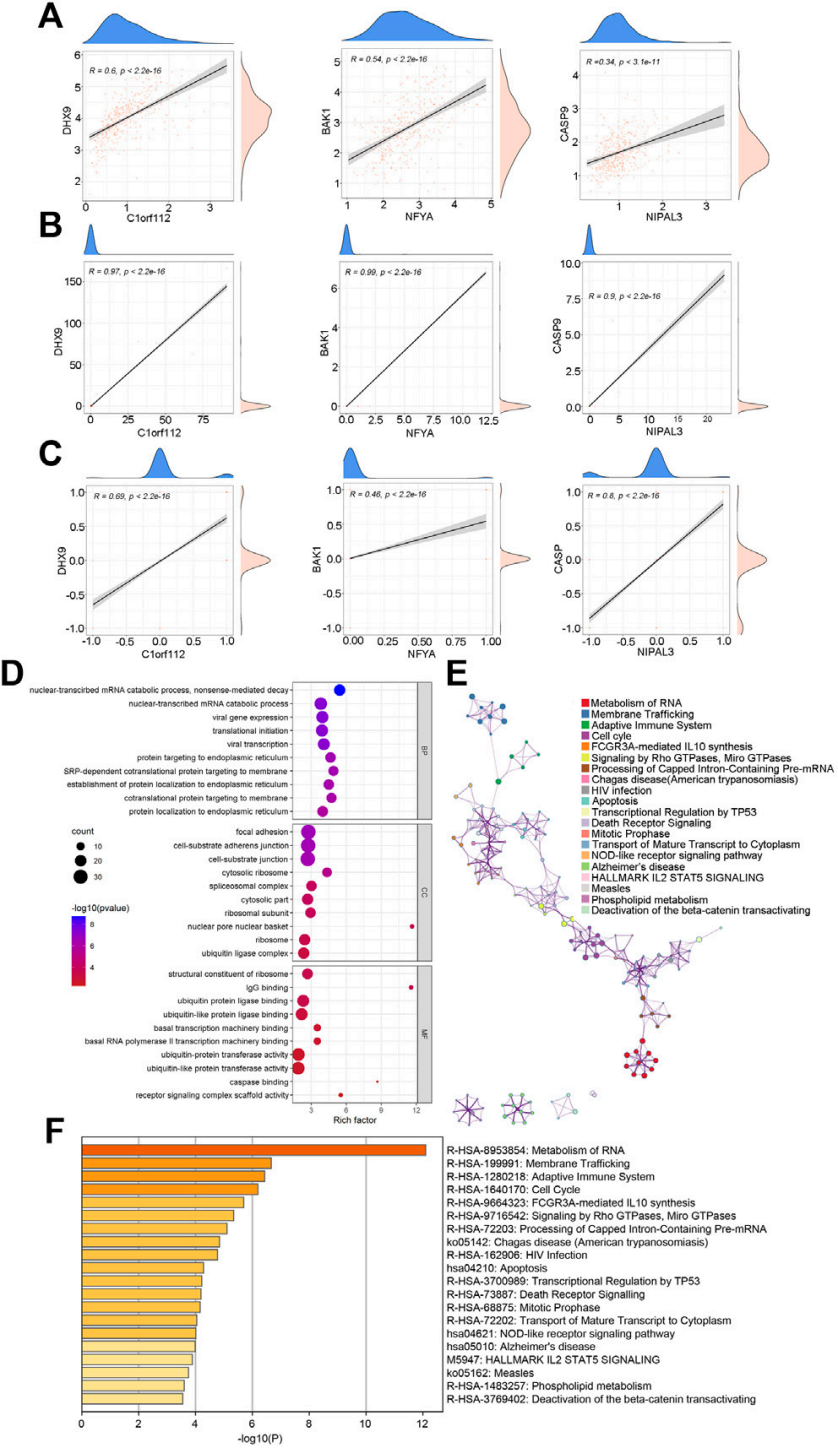
The characterization of pyroptosis-regulated genes (PRGs). **(A–C)**: Three representative genes (C1orf112, NFYA and NIPAL3) significantly associated with three pyroptosis genes (DHX9, BAK1 and CASP9) at the expression level **(A)**, mutation level **(B)**, and copy number variation level **(C)**. **(D)** Gene ontology (GO) functional classification of candidate genes. BP: biological process; CC: cellular component; MF: molecular function. **(E)** The interaction network of top20 enriched pathways. **(F)** The top20 enriched pathways of candidate genes. The vertical axis represents enriched pathways, and the horizontal axis represents the *p*-value of enrich analysis of each pathway.

To comprehensively study the genes regulated by the pyroptosis gene, correlation analysis was performed between the 56 pyroptosis genes and the remaining genes at the expression, mutation, and CNV levels. By setting a threshold of |correlation coefficient| (| Cor |) > 0.5 and adjusted *p*-value (padj) < 0.05, we obtained 9,759, 18,063, and 7,509 pyroptosis-related genes at the expression level, mutation level, and CNV level, respectively. Using the intersection set, 614 candidate genes associated with pyroptosis genes at all three levels were obtained. Figures 4A–C show the one-to-one correlation between three candidate genes (C1orf112, NFYA, and NIPAL3)and three pyroptosis genes (DHX9, BAK1, and CASP9) at the transcription level, mutation level, and CNV level, respectively (See all the 614 genes in Supplementary Table 4_cor_genes.xlsx).

We used the “ClusterProfiler” R packages for functional enrichment analysis of 614 candidate genes. The result showed that these candidate genes were strongly associated with nuclear-transcribed mRNA catabolic process, translational initiation, and other biological processes, suggesting were involved in gene expression and protein synthesis. The top10 entries for biological process (BP), molecular function (MF), and cellular component (CC) are shown (Figure 4D, *p* < 0.05, Supplementary Table 5_corsigs_GOBP.xlsx, Supplementary Table 5_corsigs_GOCC.xlsx, Supplementary Table 5_corsigs_GOMF.xlsx).

Using the metascape database, we performed pathway enrichment analysis against the Hallmark, KEGG, and Reactome pathways to investigate the signaling pathways in which 614 candidate genes are involved. 253 pathways were identified using a *p*-value <0.05 threshold, and the top 20 pathways were retrieved as major pyroptosis-regulated pathways (Supplementary Table 6_sig_pathway_metascape. xlsx). Figures 4E,F show the net network and quantitative analysis of the top 20 key pathways, respectively.

**FIGURE 5:**
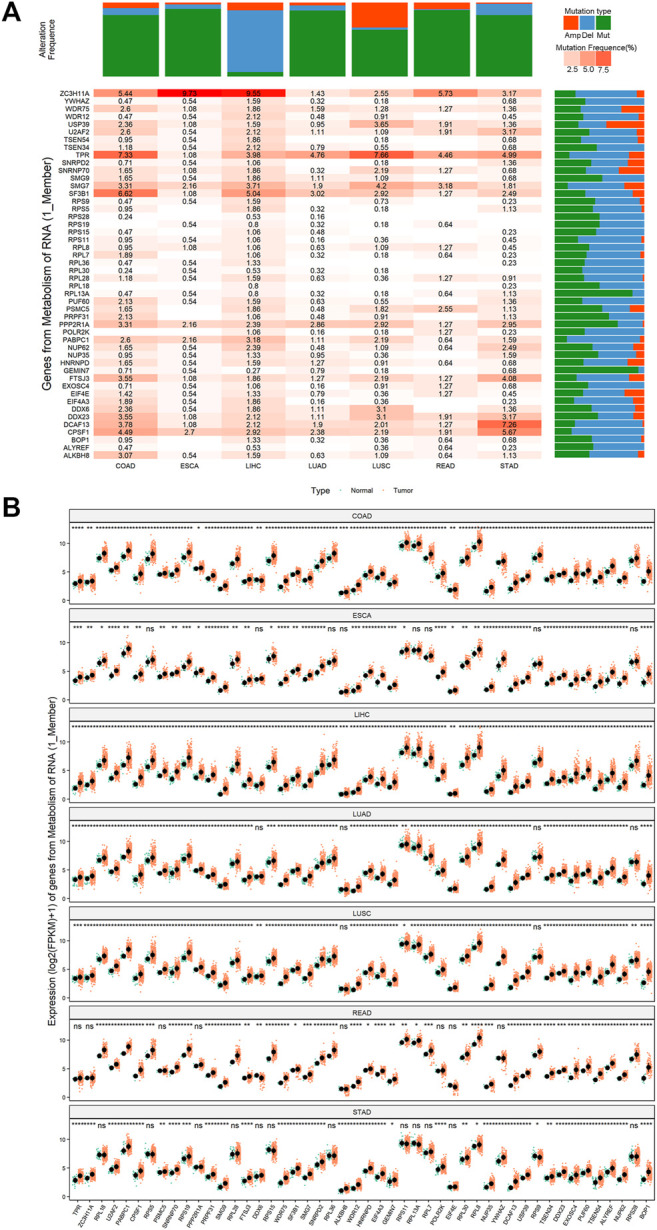
The mutation and expression profile of key genes of the key pathway “Genes from Metabolism of RNA”. **(A)** The mutation status of key genes of the key pathway “Genes from Metabolism of RNA” in pan-cancer. Mutation type is classified as: amplification (Amp, the orange color), deletion (del, the blue color), point mutation (mut, the green color). The color transition from light pink to orange indicates a trend from low mutation frequency to high mutation frequency. **(B)** The expression patterns of key genes of the key pathway “Genes from Metabolism of RNA” in pan-cancer. *Means *p* < 0.05, ** means *p* < 0.01, ***means *p* < 0.001, ****means *p* < 0.0001.

We designated 108 genes that participated in the pyroptosis-regulated pathways as pyroptosis-regulated genes (PRGs) (Supplementary Table 7_sig_gene. xlsx). The CNVs, mutations, and expression of 48 PRGs in the key pathway “Genes from Metabolism of RNA” were systematically studied. The results showed that 48 PRGs exhibited varying degrees of CNV alterations and mutation levels in pan-cancer (Figure 5A, see the rest of the pyroptosis-regulated pathways in Supplementary Figure S13 (1)–(19)). In terms of expression level, the vast majority of the 48 PRGs showed a higher level in cancer tissues compared to normal controls in pan-cancer (Figure 5B, see the rest of the pyroptosis-regulated pathways in Supplementary Figure S14 (1)–(19)).

### Generation of risk score

**FIGURE 6:**
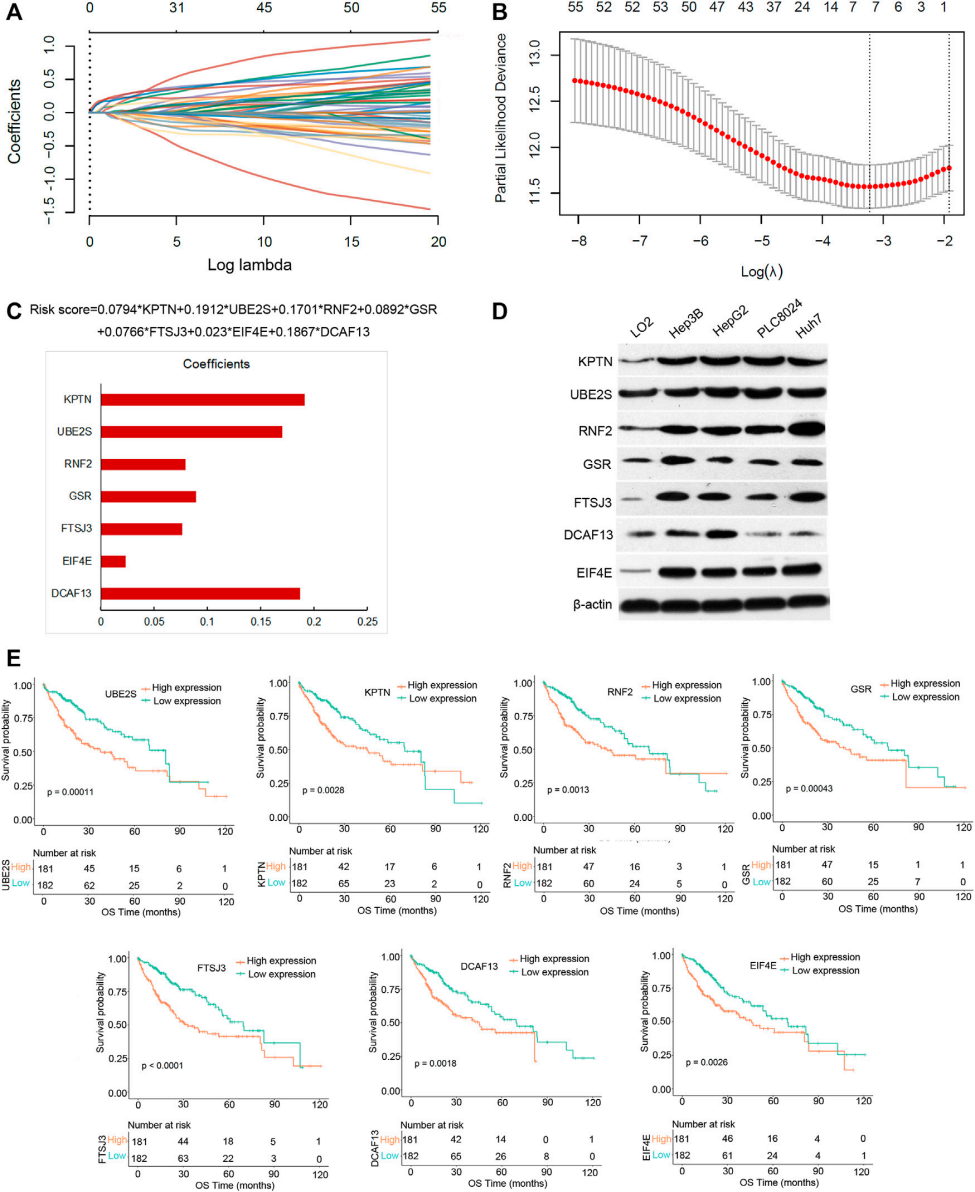
Seven pyroptosis-regulated genes with prognostic value in the training group. **(A)** The LASSO coefficient profiles of the 57 pyroptosis-regulated genes. **(B)** Cross-validation plot for the penalty term. **(C)** The risk score formula and the coefficients of seven pyroptosis-regulated genes. **(D)** Western blotting results showing the expression level of the seven pyroptosis-regulated genes in four HCC cell lines (Hep3B, HepG2, PLC8024, and Huh7) and one normal hepatocyte cell line (LO2). **(E)** Survival analysis of seven pyroptosis-regulated genes by the Kaplan-Meier Plotter method.

By performing survival analysis, we identified 57 out of 108 PRGs that showed prognostic value in HCC (Supplementary Table 8_keggsiggenes_cox.xlsx). To exclude the overfitting, we performed the LASSO-Cox regression on 57 PRGs and identified a total of seven PRGs in the training group. The screening process and results are displayed in Figures 6A,B. We then obtained a risk model consisting of seven key genes (UBE2S, KPTN, RNF2, GSR, FTSJ3, DCAF13, and EIF4E) (Figures 6A,B, Supplementary Table 9_lasso_Coefficients. xlsx). The risk score formula is: Risk score = 0.0794*KPTN+0.1912*UBE2S+0.1701*RNF2+0.0892*GSR+0.0766*FTSJ3+0.023*EIF4E+0.1867*DCAF13. Western blot was carried out to examine the protein expression of the seven PRGs in one normal liver cell line (LO2) and four HCC cell lines (Figure 6D). Single survival analyses of the seven genes were listed in Figure 6E. We observed that the high expression of the seven PRGs conferred a disadvantage over overall survival.

**FIGURE 7:**
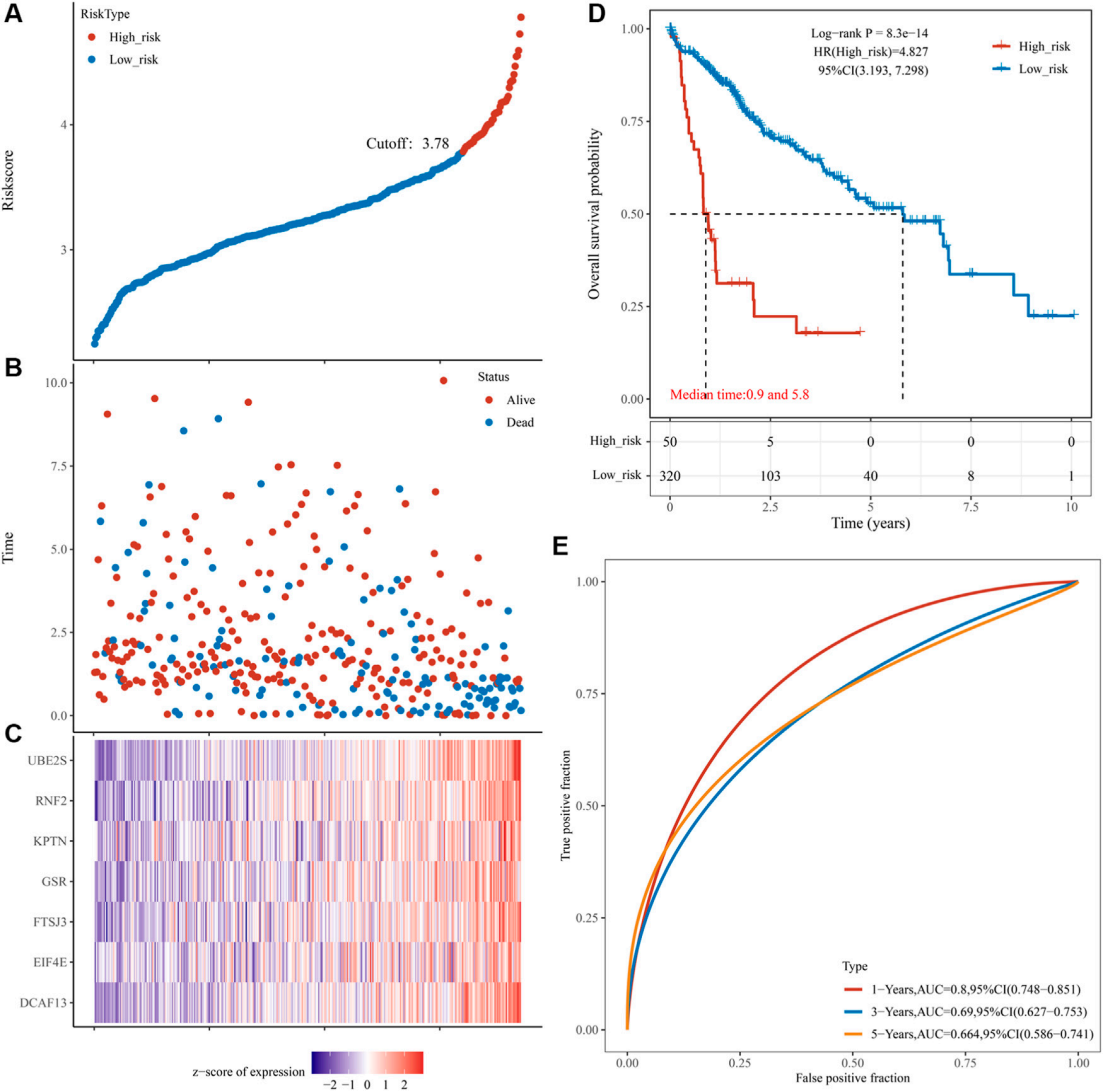
Prognostic risk score features of pyroptosis-regulated genes in the training group and validation group 1. **(A)** The scatterplot showing the riskscore from low to high in the training set. The blue color indicates low risk group. The yellow color indicates high risk group (the same below). **(B)** The scatter plot reveals the distribution of the risk score of each sample corresponding to the survival time and survival status in the training set. **(C)** The heatmap showing the gene expression patterns of the pyroptosis-regulated signature. **(D)** Kaplan-Meier Plotter of the risk model of the training set. Difference groups were compared using the log-rank test. **(E)** The ROC curve at 1, 3 and 5 years and AUC of the pyroptosis-regulated signature.

To determine the power of the risk score model in predicting HCC prognosis, we separated training group patients into high- and low-risk groups according to the best cut-off value. The risk scores distribution of the two groups is shown in Figure 7A. A scatterplot displayed the corresponding survival status of the patient with different risk scores in Figure 7B. Figure 7C is a heatmap created by expressions of UBE2S, KPTN, RNF2, GSR, FTSJ3, DCAF13, and EIF4E between the two groups. Survival analysis demonstrated that the low-risk group had an obvious survival advantage over the high-risk group (Figure 7D). We further established that the risk score was a good indicator for the 1-year survival, 3-years survival, and 5-years survival of HCC patients (Figure 6E, AUCmax = 0.8, Supplementary Table 10_TCGA_riskscore.xlsx).

**FIGURE 8:**
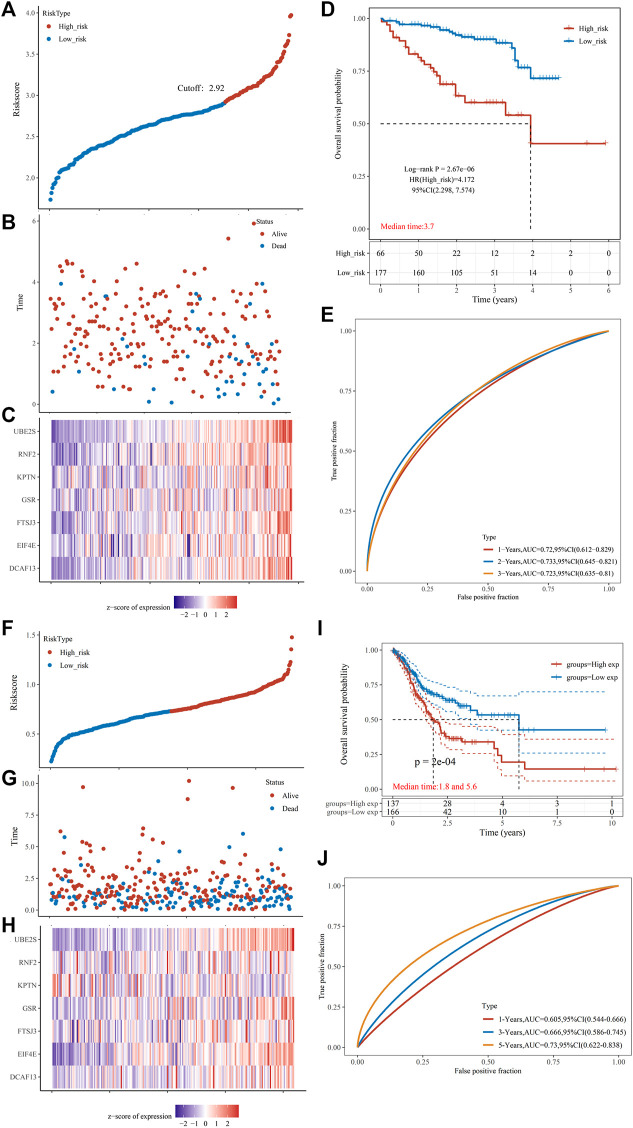
Prognostic risk score features of pyroptosis-regulated genes in the validation groups **(A)** The scatterplot displaying the risk score from low to high in the validation group 1 (ICGC). The blue color indicates low risk group. The yellow color indicates high risk group. **(B)** The scatter plot showing the distribution of the risk scores of each sample corresponding to the survival time and survival status in the validation group 1. **(C)** The heatmap illustrating the gene expression pattern of the pyroptosis-regulated signature. **(D)** Kaplan-Meier Plotter of the risk model validation group 1. Different groups were compared using the log-rank test. **(E)** The ROC curve at 1, 2 and 3 years and AUC of the pyroptosis-regulated signature. **(F)** The scatterplot showing the risk scores from low to high in the validation group 2 (HCCDB6and HCCDB17). The blue color indicates low risk group. The yellow color indicates high risk group. **(G)** The scatter plot demonstrating the distribution pattern of the risk scores of each sample corresponding to the survival time and survival status in the validation group 2. **(H)** The heatmap showing the gene expression of the pyroptosis-regulated signature. **(I)** Kaplan-Meier Plotter of the risk model validation group 2. Different groups were compared using the log-rank test. **(J)** The ROC curve at 1, 2 and 3 years and AUC of the pyroptosis-regulated signature.

To further establish the predictive power of the risk score model, we validated it in an independent dataset ICGC. Patients were divided into the low-risk and high-risk groups based on the optimal cut-off value. Figure 8A shows a distribution of risk scores in the two groups. Figure 8B shows a scatterplot of the survival status. Figure 8C shows the distribution of seven PRGs using a heat map. Survival analysis revealed that the high-risk group had poorer survival than the low-risk group (Figure 8D). The risk model exhibited excellent predictive power for 1-year, 3-years, and 5-years survival (AUCmax = 0.733, Figure 8E, Supplementary Table 10_ICGC_risk.xlsx). Another independent cohort (HCCDB6 and HCCDB17) was used as the second validation group. Patients were also divided into the low-risk and high-risk groups using the best cut-off value. Figure 8F shows a distribution of risk scores in the two groups. Figure 8G shows a scatterplot of the survival status. Figure 8H shows the distribution of seven PRGs using a heat map. Survival analysis revealed that the high-risk group had poorer survival than the low-risk group (Figure 8I). The risk model exhibited excellent predictive power for 1-year, 3-years, and 5-years survival (AUCmax = 0.730, Figure 8J, Supplementary Table 10_B6_17_Risk.xlsx).

### Clinical features, tumor microenvironment characteristics, and immunotherapy of the risk score model in HCC

**FIGURE 9:**
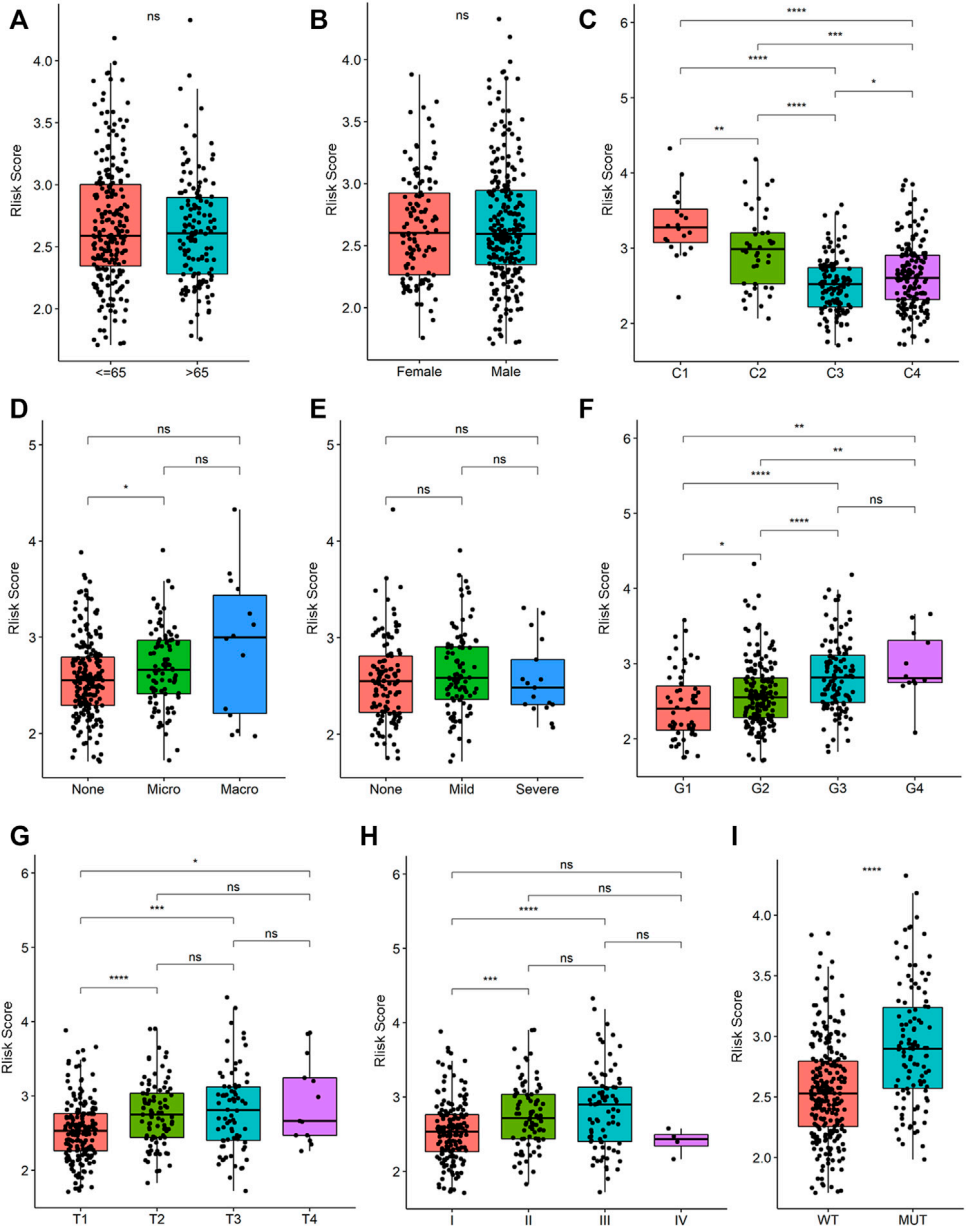
The risk score in HCC samples with different clinical traits. **(A)** Relationships between risk score and age groups (≤65 years old vs .> 65 years old). ns means no significance (the same below). **(B)** Relationships between risk score and gender (female vs. male). **(C)** Relationships between risk score and collagen proportional area (CPA) grade (C1 vs. C2 vs. C3 vs. C4). **(D)** Relationships between risk score and vascular invasion status (micro vascular invasion vs. macro vascular invasion). **(E)** Relationships between risk score and degree of hepatitis (mild hepatitis vs. severe hepatitis). **(F)** Relationships between risk score and tumor grade (G1 vs. G2 vs. G3 vs. G4). **(G)** Relationships between risk score and tumor T stage (T1 vs. T2 vs. T3 vs. T4). **(H)** Relationships between risk score and tumor TNM stage (I vs. II vs. III vs. IV). **(I)** Relationships between risk score and TP53 mutation status (wild vs. mutation).

In the training cohort, we analyzed the relationship between clinical manifestations and the risk score. Figures 9A,B show that there were no significant differences between age groups (≤65 and >65 years old) and gender. A previous study reported that the collagen proportional area (CPA) measurement may be a useful prognostic indicator for HCC (Guo et al., 2022). Our study demonstrated that there were statistically significant differences among all CPA stages (Figure 9C, C1: 0%–5%, C2: 5%–10%, C3: 10%–20%, C4: >20%). A significant difference was observed between patients with and without microvascular invasion (Figure 9D). Interestingly, there was no correlation between the risk score and the severity of HCC (Figure 9E). Figures 9F–H shows the significant increase in the risk scores when the tumor grade (G1-G3), T stage (T1-T3), and TNM stage (I-III) increased. In addition, patients with a p53 mutation had a significantly higher risk score than those without the p53 mutation (Figure 9I). To our knowledge, no direct evidence demonstrated correlation between p53 and pyroptosis. Normal but not mutant p53 transcriptionally upregulates caspase-1, which acts as a tumor suppressor in breast cancer (Celardo et al., 2013). The mutant p53 is related with chronic inflammation, promoting tumor growth and immune dysfunction across different types of human cancers, including HCC (Agupitan et al., 2020). Inflammation induced by cancer cell pyroptosis leads to attenuated antitumor immunity due to the differential duration and released cellular contents (Hou et al., 2021). However, further solid exploitations are needed to find the effect of normal or mutant p53 on pyroptosis in HCC.

**FIGURE 10:**
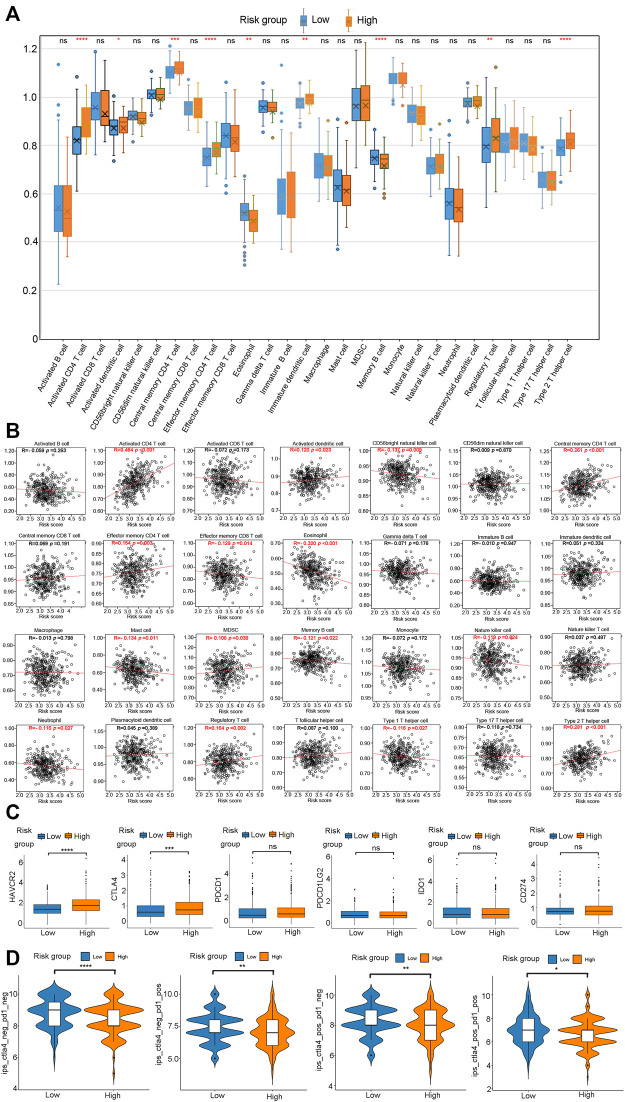
Evaluation of the infiltrating immune cells, checkpoints, and immune checkpoint blockade response between the high- and low-risk group. **(A)** Comparasion of each TME-infiltrating cell between high- and low-risk groups. **(B)** Correlations between risk score and immune cell types. **(C)** The expression of six immune checkpoints (HAVCR2, CTLA4, PDCD1, PDCD1LG2, IDO1, CD274) in the high - and low-risk groups. **(D)** Immunotherapy response between high- and low-risk groups.

Next, we determined the pattern of immune cell infiltration in 370 HCC patients using the ssGSEA (Xiao et al., 2020). Results showed that the high-risk group possessed higher levels of activated CD4^+^ T cells, activated dendritic cells, central memory CD4^+^ T cells, effector memory CD4^+^ T cells, immature dendritic cells, regulatory T cells, type 2 T helper cells compared with the low-risk group. Meanwhile, high-risk group displayed lower infiltration levels of eosinophils and memory B cells (Figure 10A, *p* < 0.05, Supplementary Table 11_ssgsea-xlsx, Supplementary Table 12_SSgsea_cluster.xlsx). Pearson correlation analysis was performed to assess the association between risk score and the abundance of immune cells. As shown in Figure 10B, the risk score was positively correlated with activated CD4^+^ T cells, activated dendritic cells, central memory CD4^+^ T cells, effector memory CD4^+^ T cells, MDSC, regulatory T cells, and type 2 T helper cells and negatively correlated with CD56bright natural killer cells, effector memory CD8^+^ T cells, eosinophils, mast cells, memory B cells, nature killer cells, neutrophils, and type 1 T helper cells.

Immunotherapy, particularly immune checkpoint blocking (ICB) therapy, is currently at the forefront of anticancer drugs. The immunoreaction of HCC has emerged as a promising topic worthy of further investigation. Therefore, we analyzed six key immune checkpoint inhibitor genes (HAVCR2, CTLA‐4, PDCD1, PDCD1LG2, IDO1, and CD274) in an HCC cohort (Kanehisa et al., 2017; Ye et al., 2021; Zhu et al., 2021). The correlation analysis revealed that HAVCR2 (*p* < 0.0001) and CTLA‐4 (*p* < 0.001) expression was significantly up-regulated in the high-risk group than in the low-risk group (Figure 10C). There was no significant difference between the risk groups and the transcriptional levels of PDCD1, PDCD1LG2, IDO1, and CD274 (Figure 10C). These results suggested that the risk model could distinguish different responses to current popular ICB treatments. Since PD-L1 and PD-1 antibodies show significant efficacy in the treatment of various cancers, we determined the applicability of risk score in HCC. We obtained a profile of immunotherapy-undergoing TCGA-LIHC patients from the TCIA database. The result demonstrated that the high-risk group was associated with a lower immune checkpoint inhibitor (ICI) score and immunotherapy sensitivity than the low-risk group (Figure 10D). In a nutshell, HCC patients in the low-risk group are distinguished by a high level of immune infiltration, a better prognosis, and a favorable response to ICI therapy.

## Discussion

Due to its high morbidity and mortality rate, HCCHCC continues to pose a serious threat to human health. Currently, the prognosis and treatment of HCC patients largely rely on the pathological examination, AJCC TNM, and BCLC stage (Bruix et al., 2016). The existing HCC diagnosis and prognosis approaches are insufficiently sensitive. Therefore, a large number of novel and precise diagnostic and prognostic biomarkers are required. A growing number of researchers have demonstrated that pyroptosis has both tumor-promoting and anti-tumor effects. However, the majority of researchers concentrated on only one or a few pyroptosis genes; hence, little attention has been paid to the study of signal pathways controlled by the pyroptosis genes.

This study revealed extensive alterations in pyroptosis genes at the transcriptional, mutation, and CNV levels in HCC. The correlation between pyroptosis genes and HCC prognosis was investigated, and 614 candidate genes were found to be significantly associated with pyroptosis genes at mutation, amplification, and expression levels. Using pathway enrichment analysis, we identified 108 PRGs that were significantly enriched in Hallmark, KEGG, and Reactome pathways. Fifty-seven out of the 108 PRGs exhibited a significant relationship with the prognosis of HCC. Using the LASSO Cox analysis, we constructed a risk score model based on pyroptosis-regulated signaling pathways using seven PRGs, namely UBE2S, KPTN, RNF2, GSR, FTSJ3, DCAF13, and EIF4E. This risk model was evaluated in an external validation cohort LIRI-JP, where it demonstrated a high prediction accuracy. We constructed a nomogram to estimate the survival of patients who were diagnosed with HCC using the risk score model. According to the findings of the ssGSEA analysis, the high-risk group possessed higher levels of activated CD4^+^ T cells, activated dendritic cells, central memory CD4^+^ T cells, effector memory CD4^+^ T cells, immature dendritic cells, regulatory T cells, type 2 T helper cells Furthermore, the high-risk group also had higher transcriptional levels of HAVCR2 and CTLA‐4 than the low-risk group, indicating that they may have a distinct immune pattern. We also found that the high-risk group had a lower ICI score and a decreased sensitivity to immunotherapy than the low-risk group.

In 2001, D'Souza et al. proposed the term “pyroptosis”. Since then, many researchers have investigated this novel pro-inflammatory programmed cell death (Fink and Cookson, 2005). Due to its pro-tumor and anti-tumor effects, pyroptosis has been an intriguing topic in cancer research in recent years (Xia et al., 2019). On the one hand, pyroptosis exerts beneficial effects on skin cancer, colorectal cancer, and liver cancer (Shi et al., 2015; Zhao et al., 2018; Sharma et al., 2019; Chen et al., 2020; Fenini et al., 2020). On the other hand, tumor cells undergoing pyroptosis may secrete inflammatory molecules that provide a survival benefit to their companion cells. Fu et al. constructed a prognostic model for predicting HCC prognosis using three pyroptosis genes, namely GSDME, GPX4, and SCAF11. (Fu and Song, 2021). However, signal pathways and genes that regulate pyroptosis in HCC have been the subject of few studies.

In the present study, seven genes regulated by pyroptosis, namely UBE2S, KPTN, RNF2, GSR, FTSJ3, DCAF13, and EIF4E were used to establish an independent risk score model. UBE2S or ubiquitin-conjugating enzyme E2 S was found to be up-regulated in HCC (Ma et al., 2018; Pan et al., 2018). In a total of 845 HCC patients, the elevated expression of UBE2S was significantly associated with higher tumor grade, larger tumor volume, vascular invasion, higher serum AFP level, advanced TNM stage, recurrence, and poorer outcomes (Pan et al., 2018). UBE2S exerts its oncogenic effects by enhancing the ubiquitination and degradation of p53, p27, and PTEN (Pan et al., 2018; Gui et al., 2021; Zhang et al., 2021). Mutations in KPTN mutation are closely related to a syndrome characterized by macrocephaly, neurodevelopmental delay, and seizures (Baple et al., 2014). To the best of our knowledge, however, no studies have elucidated its role in cancer. This study revealed that high expression of KPTN is associated with shorter survival in HCC patients, implying that suppressing KPTN may be an effective treatment strategy for HCC. High RNF2 expression is associated with poor overall survival and promotes tumor cell growth and metastasis in HCC (Qu and Qu, 2017). GSR, or glutathione reductase, is one of the major determinants of HCC in a complicated and context-dependent manner (McLoughlin et al., 2019). FTSJ3 is one of the RNA methyltransferases (RNMTs) which regulates RNA structures, properties, and biological functions. Kaplan-Meier analysis revealed that copy number amplification of FTSJ3 is associated with a shorter overall survival time in breast cancer (Manning et al., 2020). DCAF13, an estrogen receptor-binding protein, is overexpressed and associated with a poor prognosis in HCC (Qiao et al., 2019; Luo et al., 2020). The staining intensity of the EIF4E protein is significantly and positively correlated with high serum AFP level, high gamma-glutamyl transferase level, and vascular invasion of HCC (Cao et al., 2019).

Pyroptosis is closely associated with inflammation. However, chronic inflammation exerts tumor-promoting effects by inhibiting specific immune cells’ anti-tumor function, including activated CD8^+^ T cells and Natural Killer cells (NK) (Raposo et al., 2015). Our study revealed that the high-risk group lacks immune cells, such as the activated CD8^+^ T cell, CD56 bright Natural killer cell, effector memory CD8^+^ T cell, eosinophil, natural killer cell, and type I T helper cell. In CTLA-4/PD-1 immunotherapy, the high-risk group had a lower ICI score and was less sensitive to immunotherapy than the low-risk group. Therefore, immune-promoting and immunosuppressive environments are essential for effective clinical treatment. Our findings confirmed that pyroptosis can be used to classify the subtypes and landscapes of TME, as well as influence the clinical response to ICB. The established risk score model can help to predict the survival, TME status, and response to immunotherapy in HCC.

## Conclusion

In summary, our risk score model based on pyroptosis-regulated genes can be used to predict the prognosis of patients, immune cell infiltration characteristics, and response to immunotherapy in patients with HCC.

## Data Availability

The datasets presented in this study can be found in online repositories. The names of the repository/repositories and accession number(s) can be found in the article/Supplementary Material.

## Abbreviations

HCC, hepatocellular carcinoma; LIHC, liver hepatocellular carcinoma; ASC, apoptosis-associated speck-like protein containing a caspase recruitment domain; GSDMD, gasdermin D; PFD, pore-forming domain; LPS, lipopolysaccharide; GSDME, gasdermin E; DAC, 5-aza-2-deoxycytidine; GSDMC, gasdermin C; TME, tumor microenvironment; TCGA, The Cancer Genome Atlas; LIRI-JP, liver cancer-RIKEN JP project; CNV, copy number variation; PRGs, pyroptosis-regulated genes; MSIGDB, the Molecular Signatures Database; OS, overall survival; BP, biological process; MF, molecular function; CC, cellular component; PPI, protein-protein interaction; ESCA, esophageal cancer; LUSC, lung squamous cell carcinoma; LUAD, lung adenocarcinoma; STAD, stomach cancer; COAD, colon cancer; READ, rectum cancer; CPA, collagen proportional area; ICI, immune checkpoint inhibitor; AFP, Alpha-fetoprotein.
