# Supplementary material for: A novel pyroptosis-regulated gene signature for predicting prognosis and immunotherapy response in hepatocellular carcinoma
Source: Front Mol Biosci. 2022 Sep 5;9:890215. doi: 10.3389/fmolb.2022.890215 (PMC9575690; doi:10.3389/fmolb.2022.890215)
Supplement: Supplementary file 2 [file Table1.docx]

**Supplementary Figures**


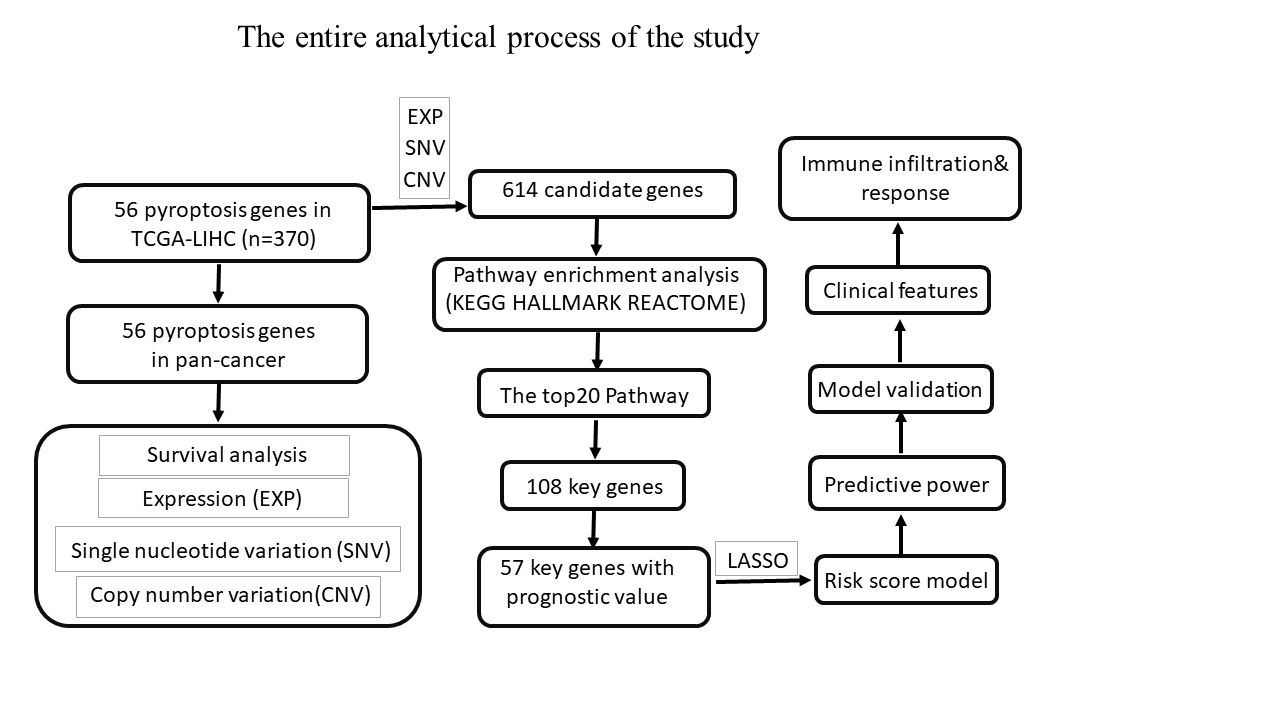


**Figure S1** The entire analytical process of the study.


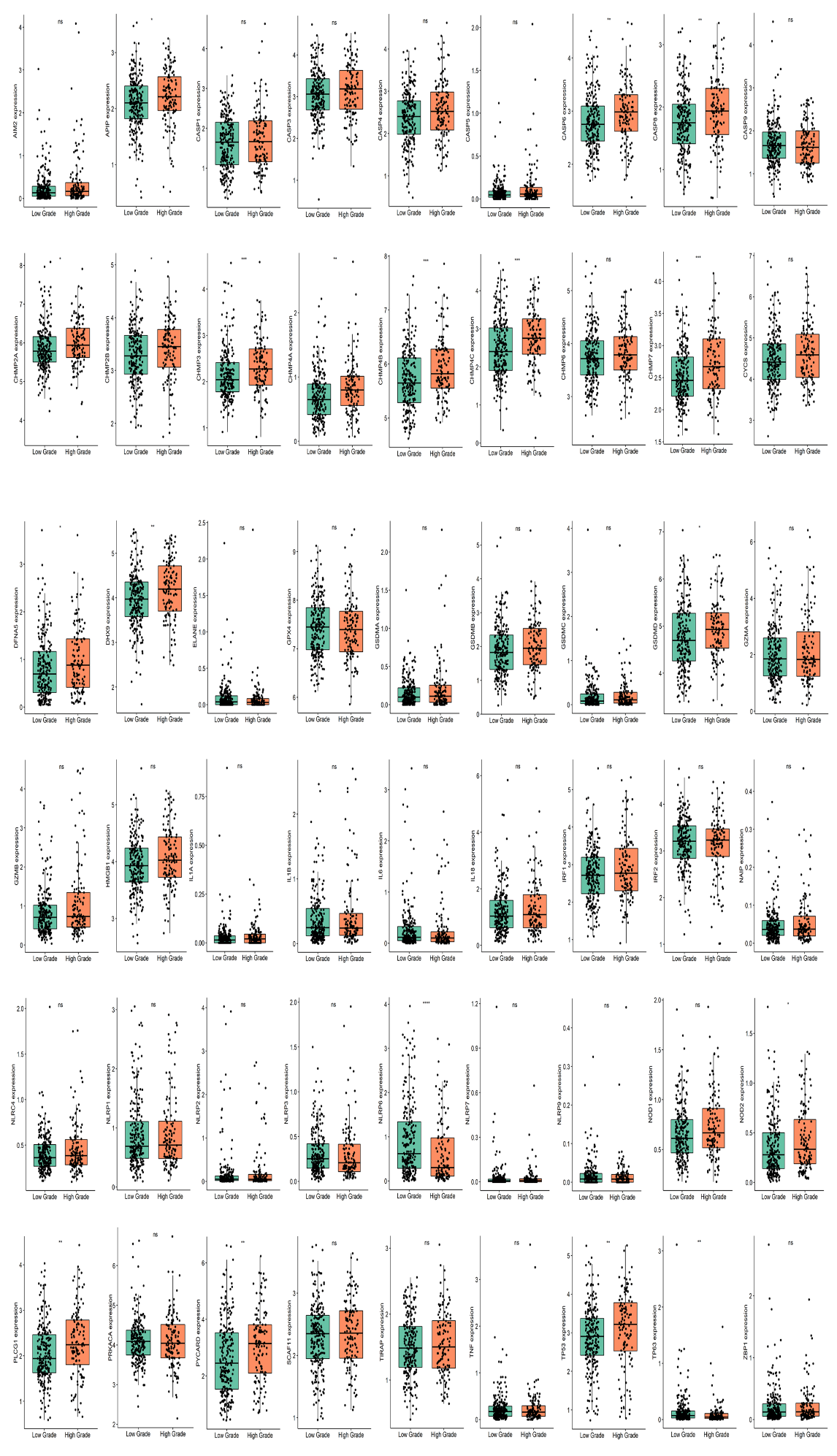


**Figure S2** The relationship between tumor grade and expression level of 56 pyroptosis genes in HCC.


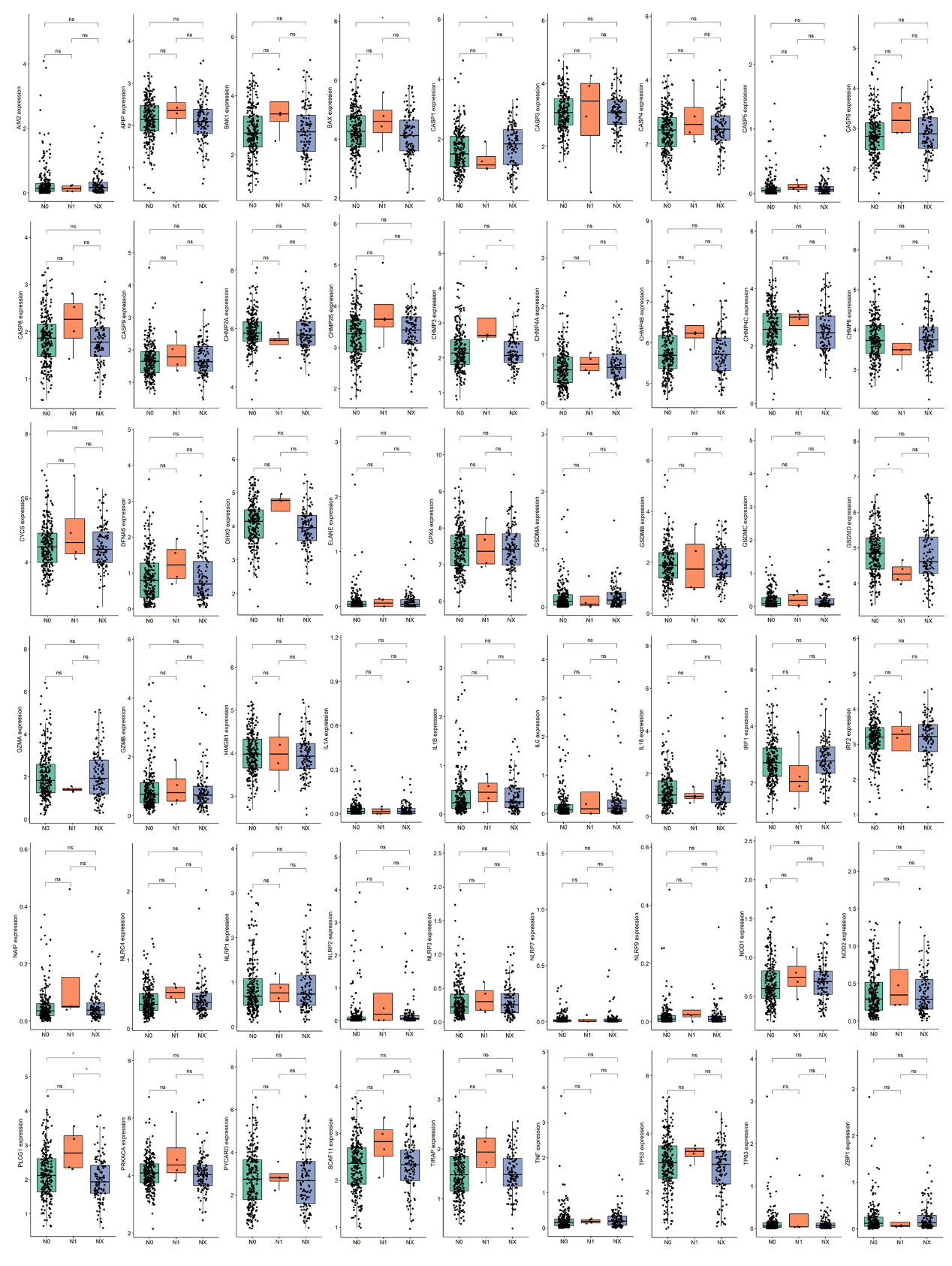


**Figure S3** The relationship between tumor node metastasis (N0, N1, NX) and expression level of 56 pyroptosis genes in HCC.


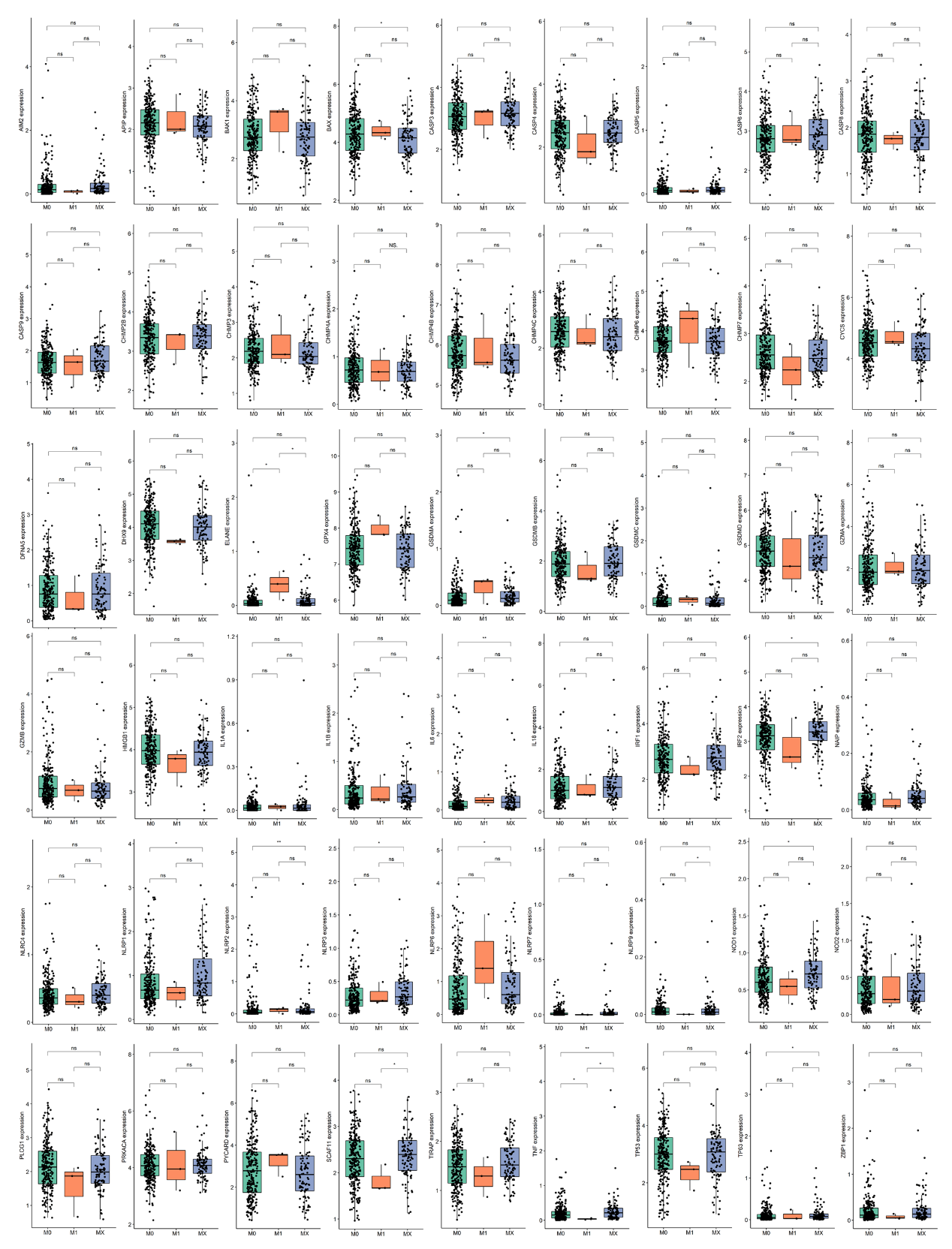


**Figure S4** The relationship between tumor distant metastasis (M0, M1, MX) and expression level of 56 pyroptosis genes in HCC.


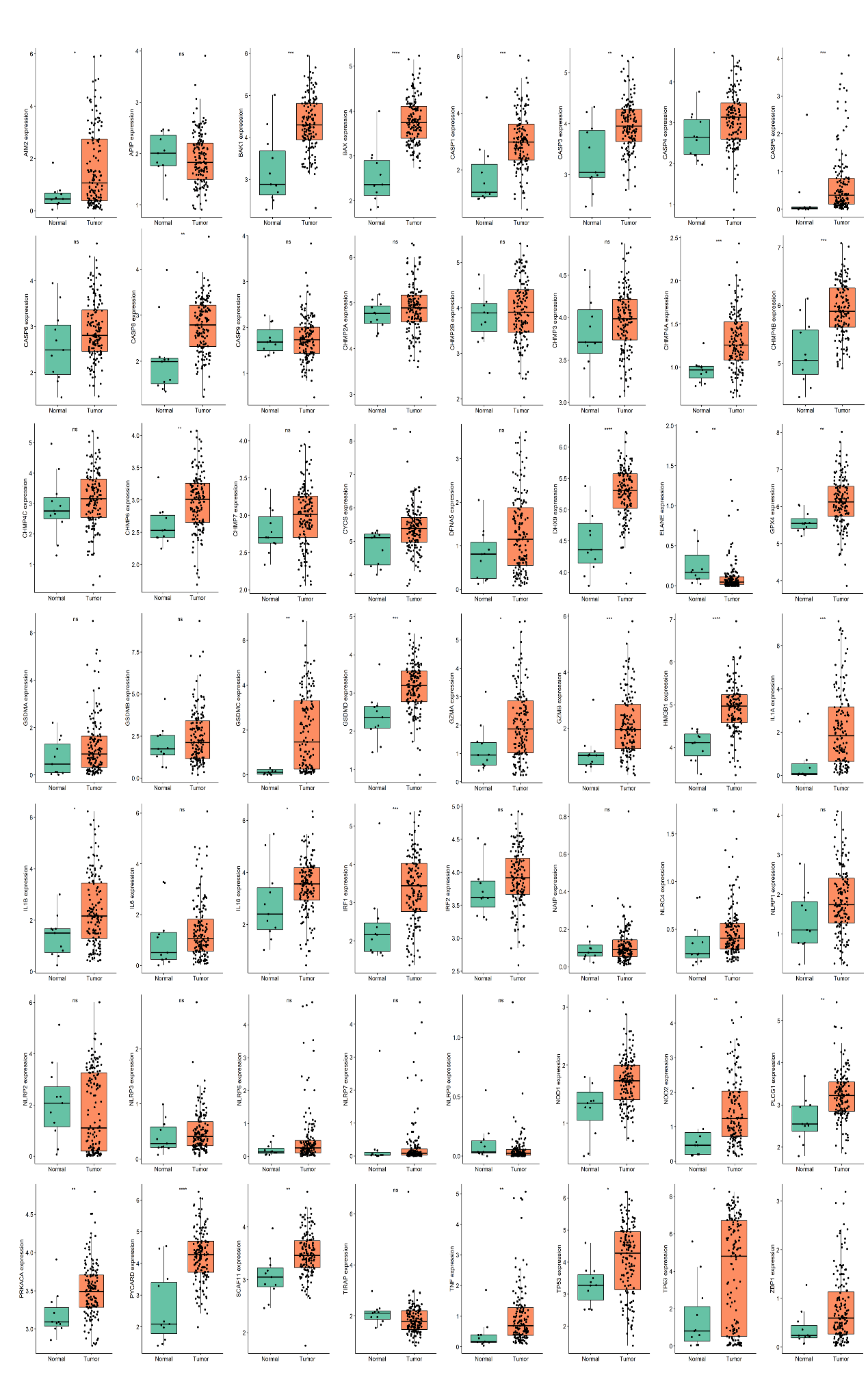


**Figure S5** Expression levels of 56 pyroptosis genes in ESCA compared to normal tissue.


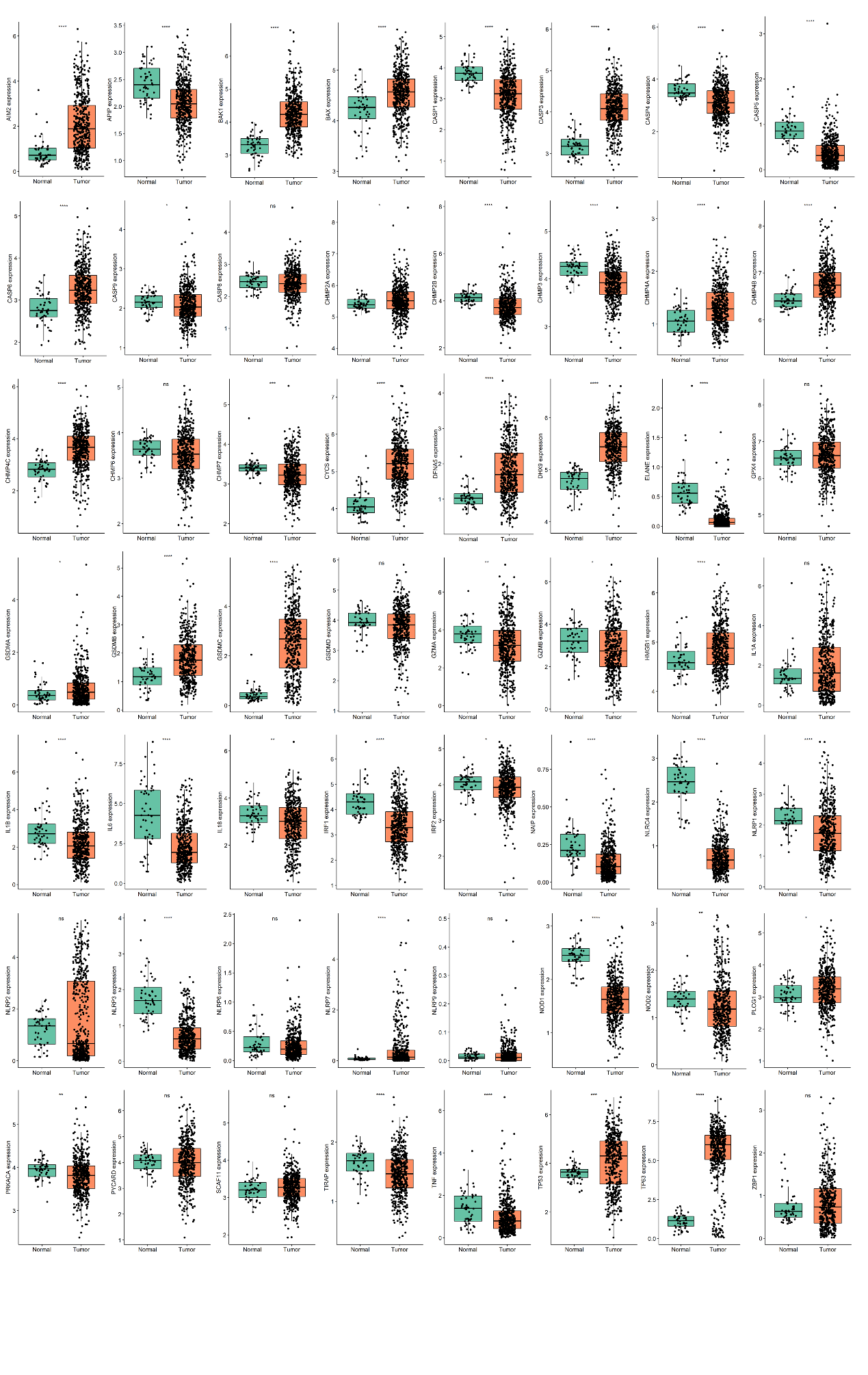


**Figure S6** Expression levels of 56 pyroptosis genes in LUSC compared to normal tissue.


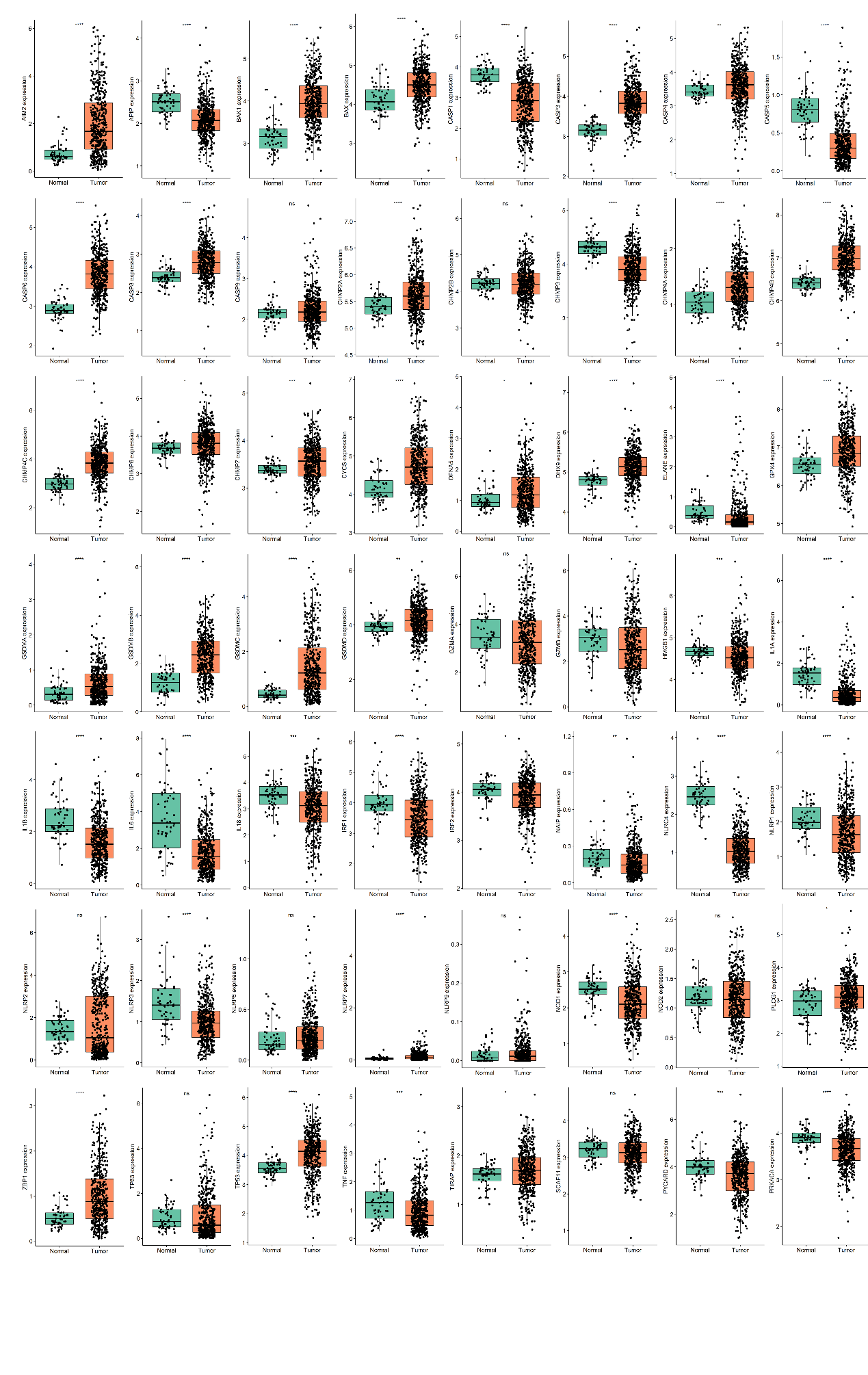


**Figure S7** Expression levels of 56 pyroptosis genes in LUAD compared to normal tissue.


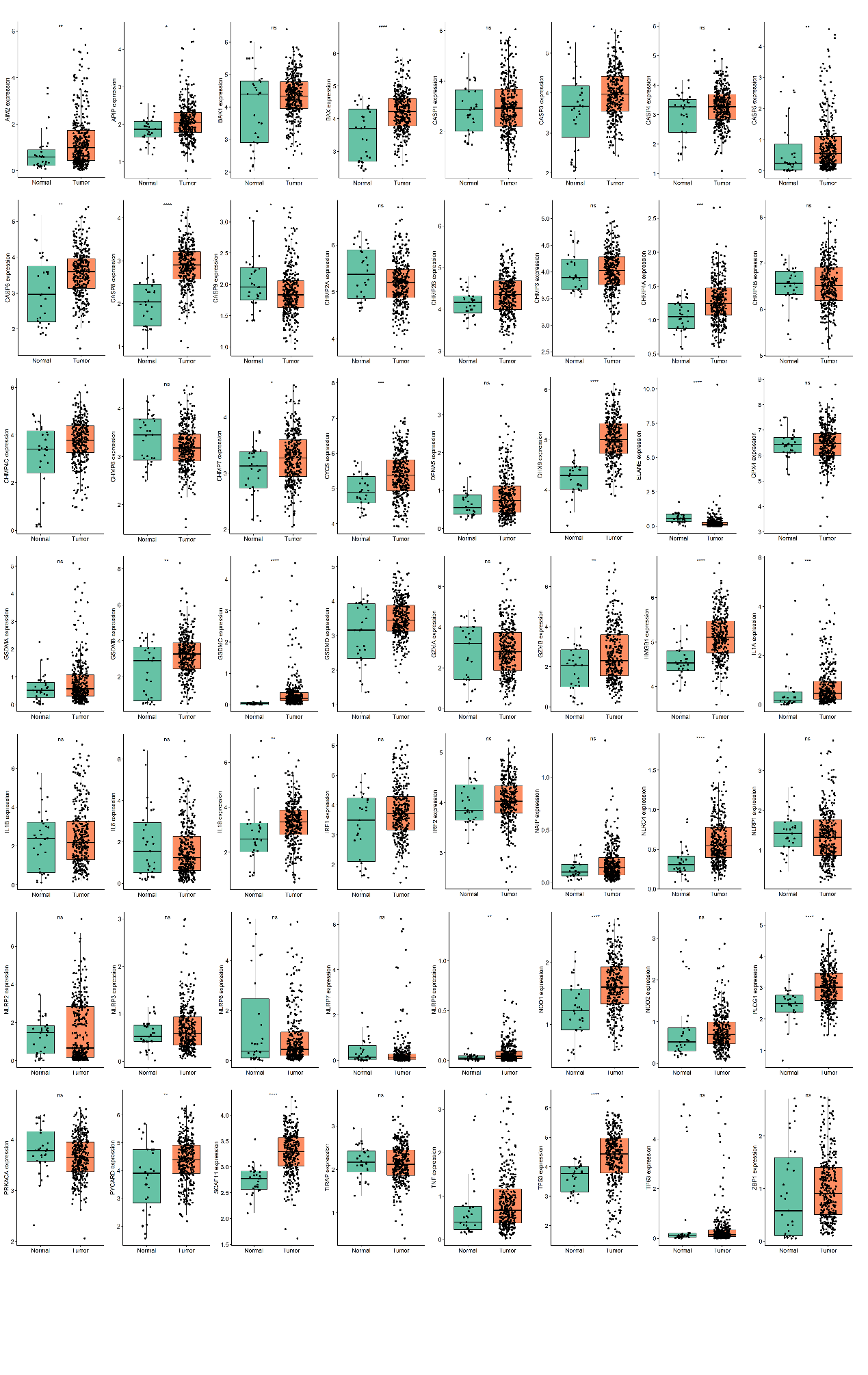


**Figure S8** Expression levels of 56 pyroptosis genes in STAD compared to normal tissue.


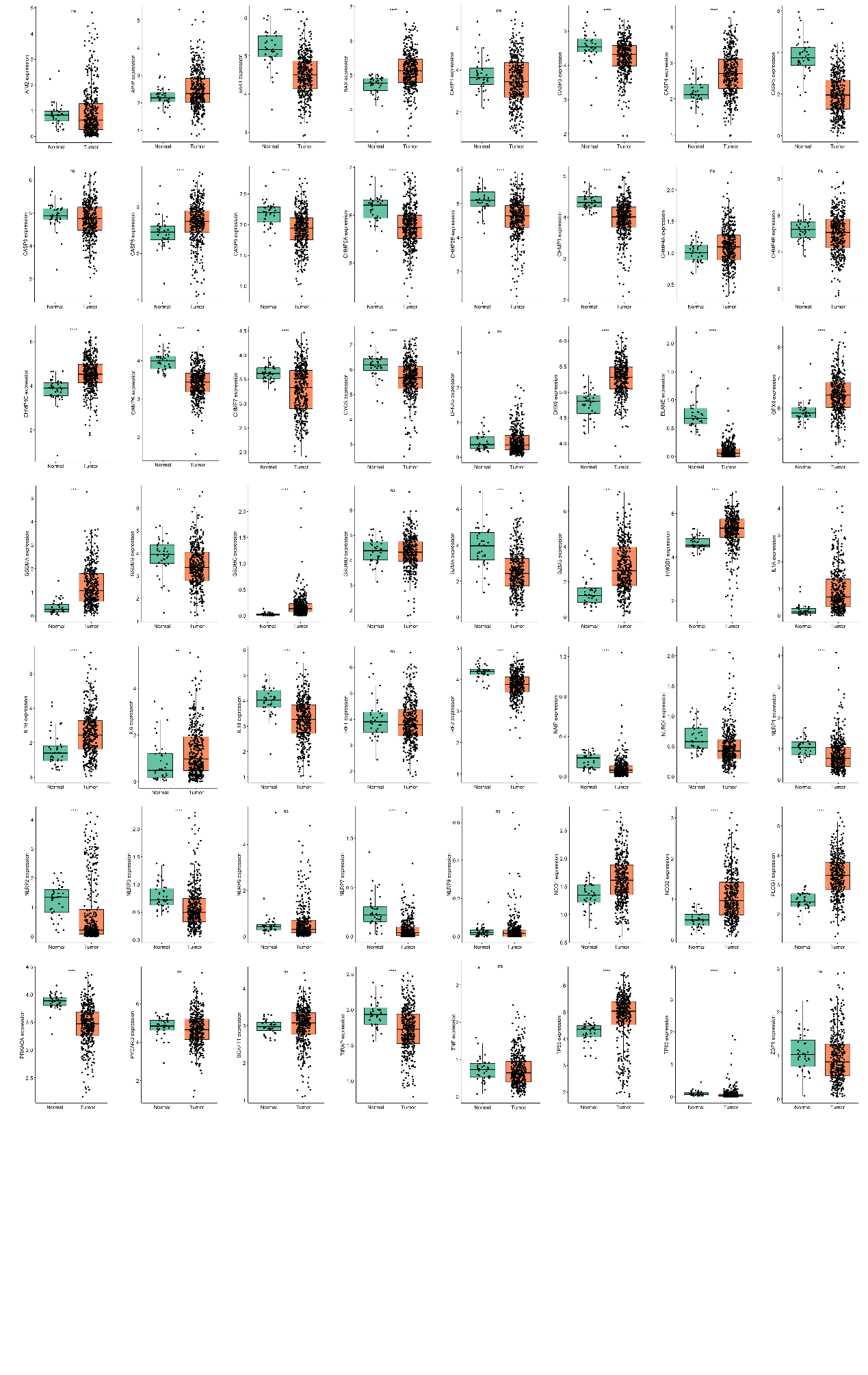


**Figure S9** Expression levels of 56 pyroptosis genes in COAD compared to normal tissue.


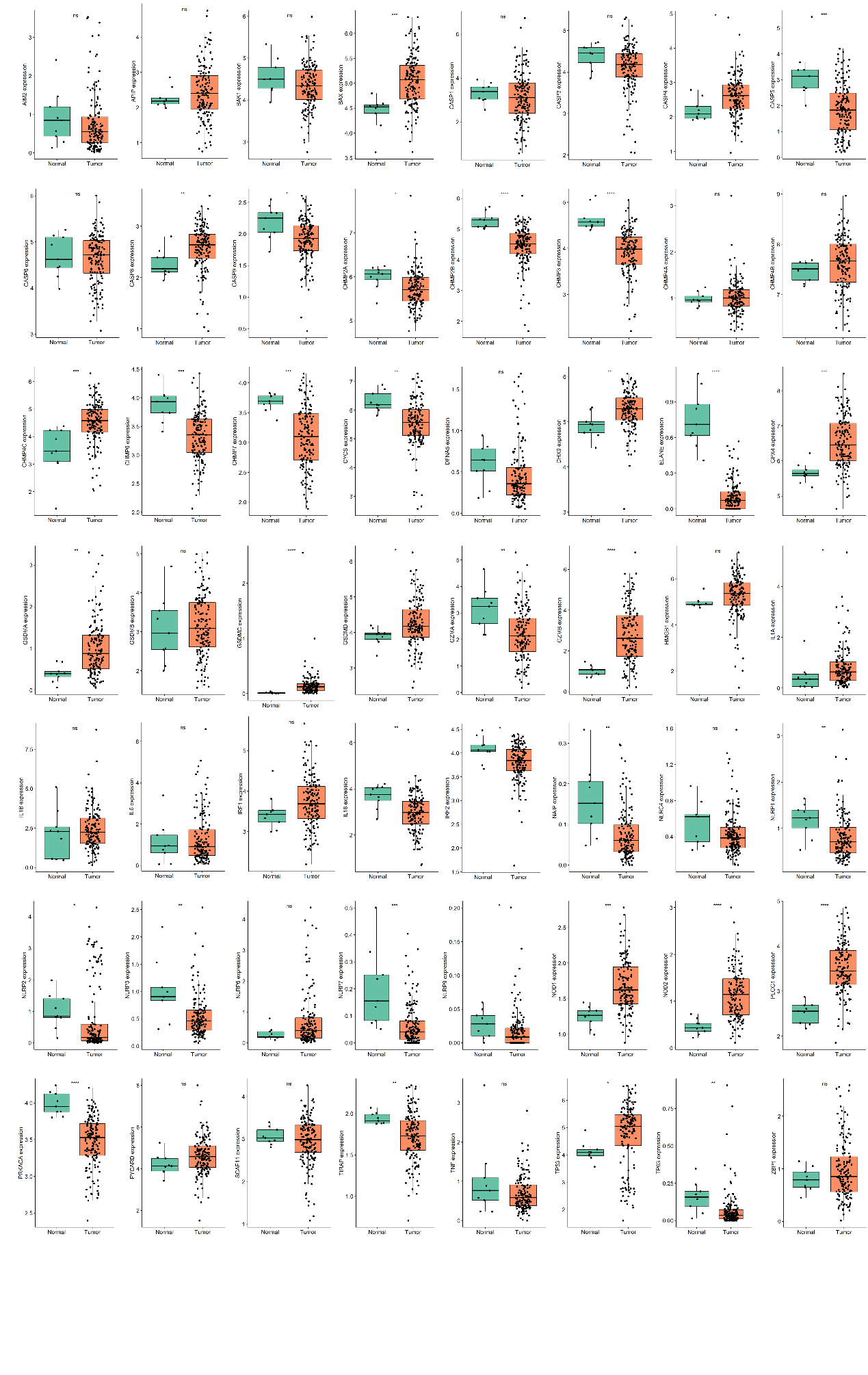


**Figure S10** Expression levels of 56 pyroptosis genes in READ compared to normal tissue.


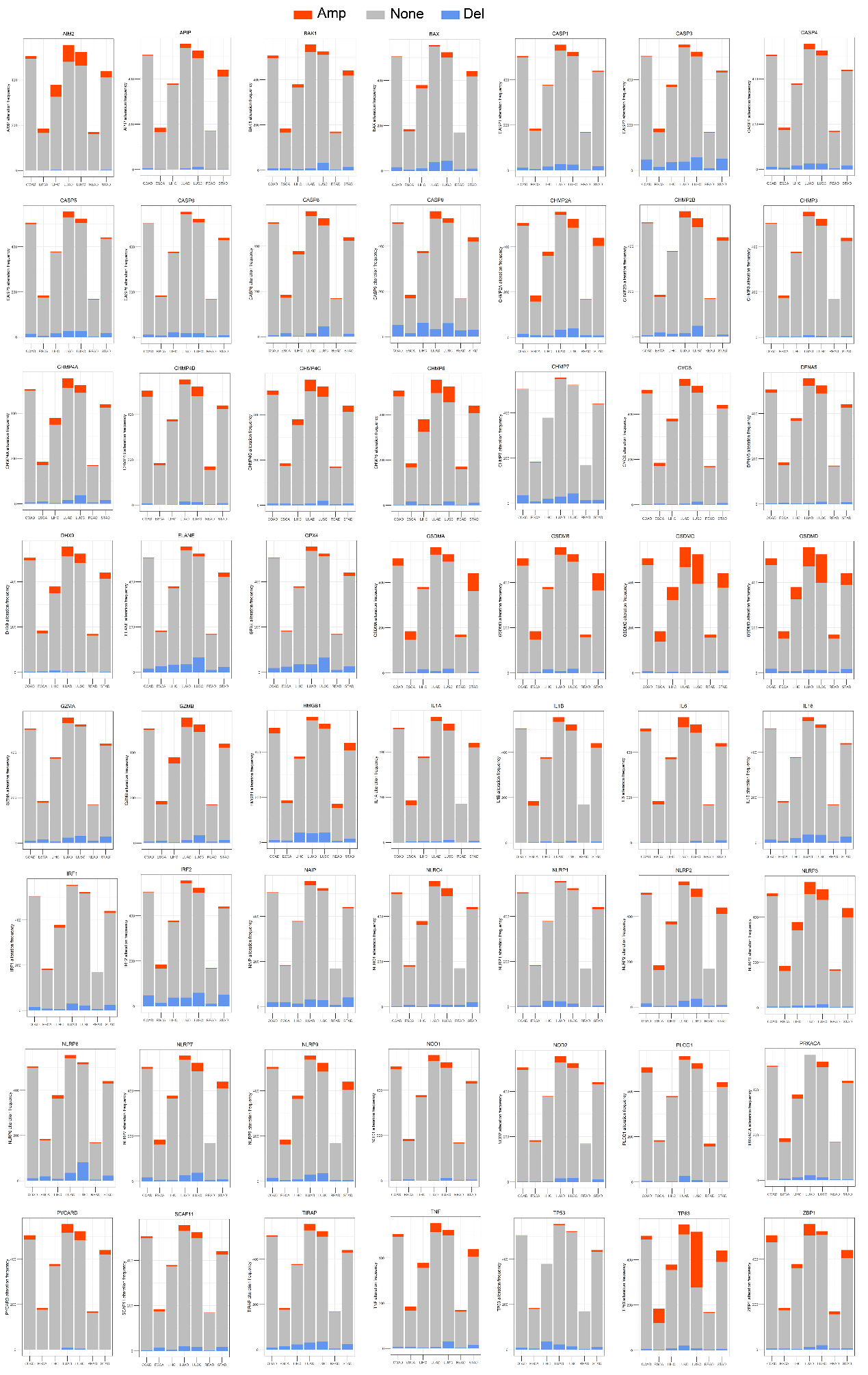


**Figure S11** CNV alterations of 56 pyroptosis genes in pan-cancer


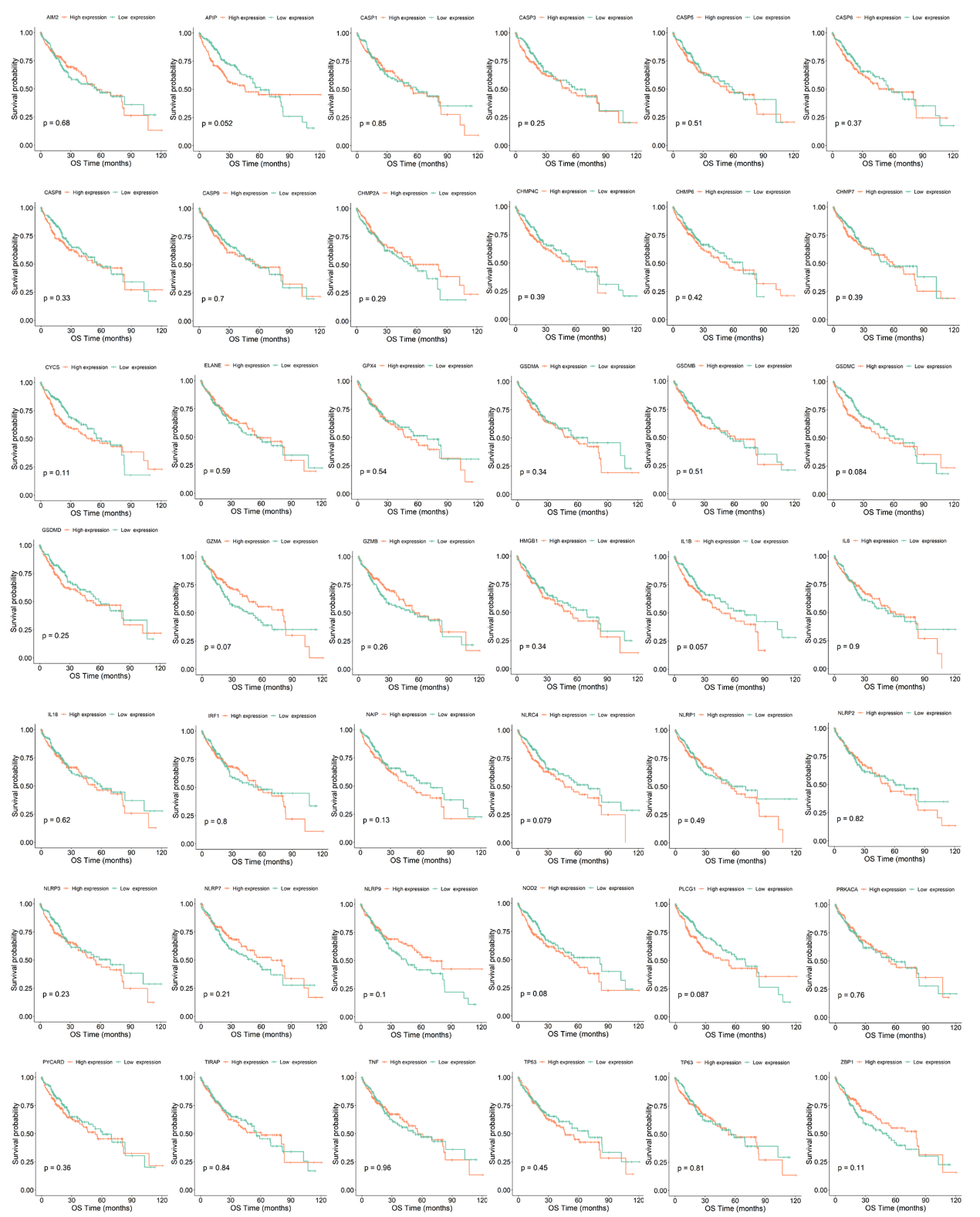


**Figure S12** Survival analysis of the rest 42 pyroptosis genes in HCC.


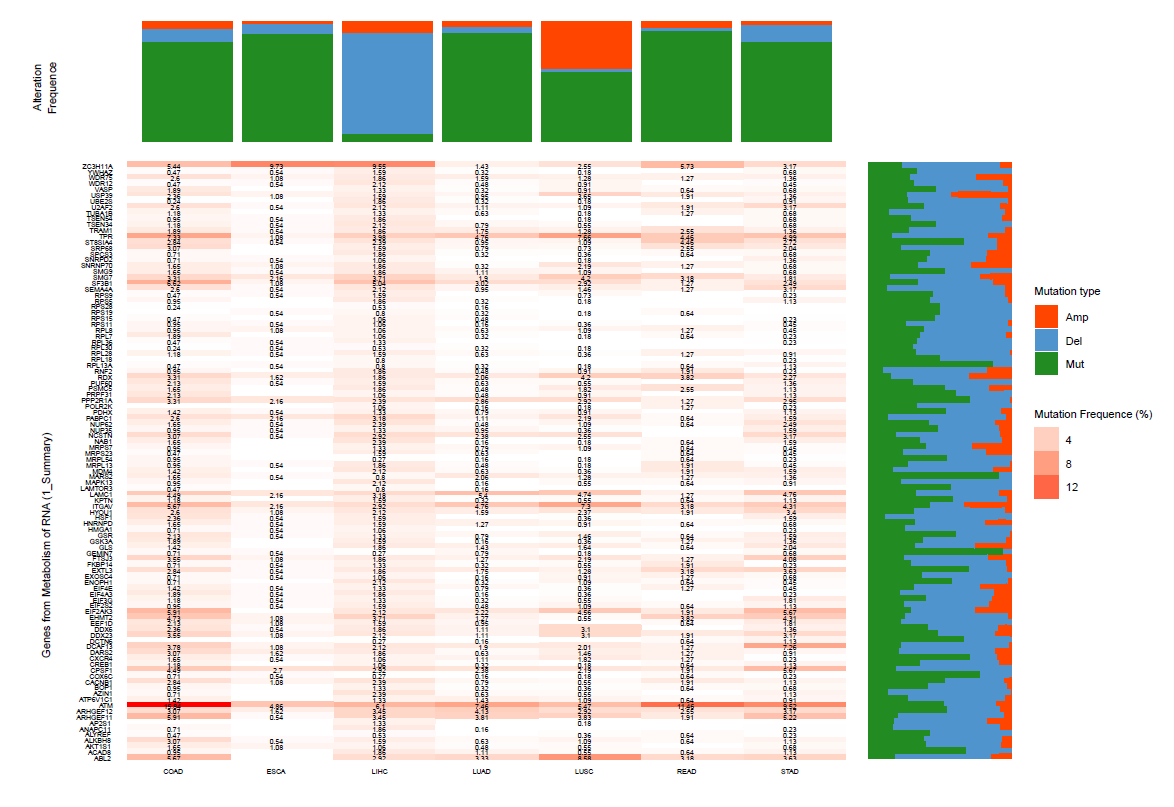


**Figure S13** (1) The copy number variances (CNVs) and mutation profile of genes in the key pathways
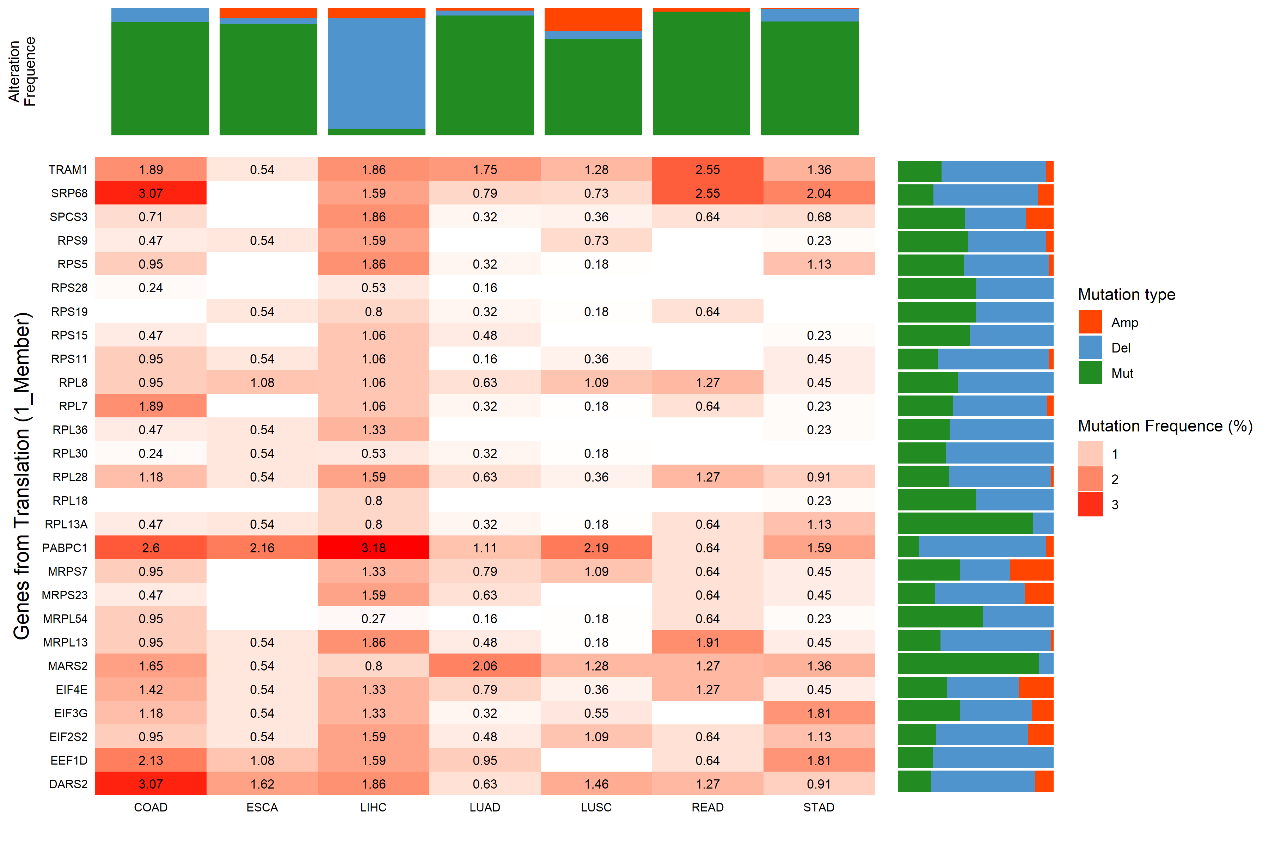


**Figure S13** (2) The copy number variances (CNVs) and mutation profile of genes in the key pathways


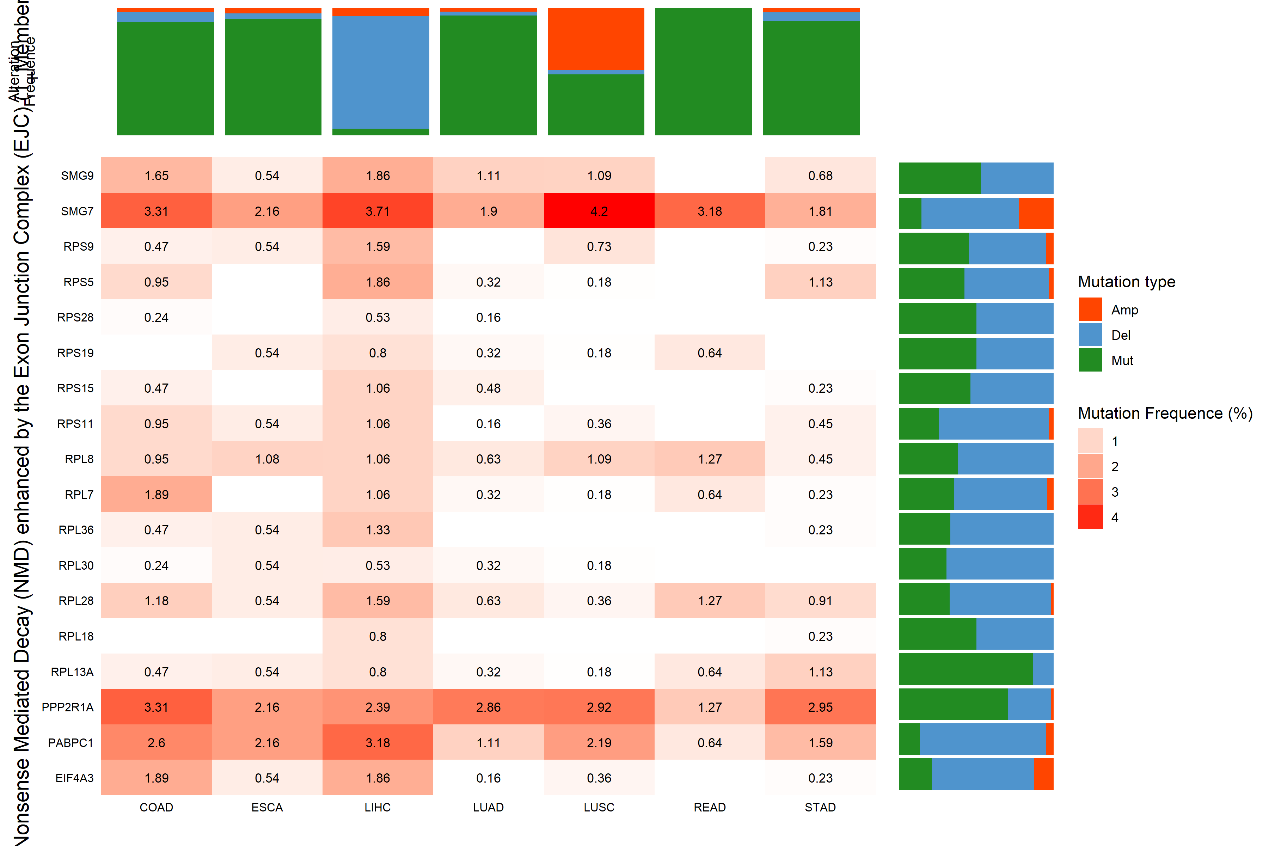


**Figure S13** (3) The copy number variances (CNVs) and mutation profile of genes in the key pathways


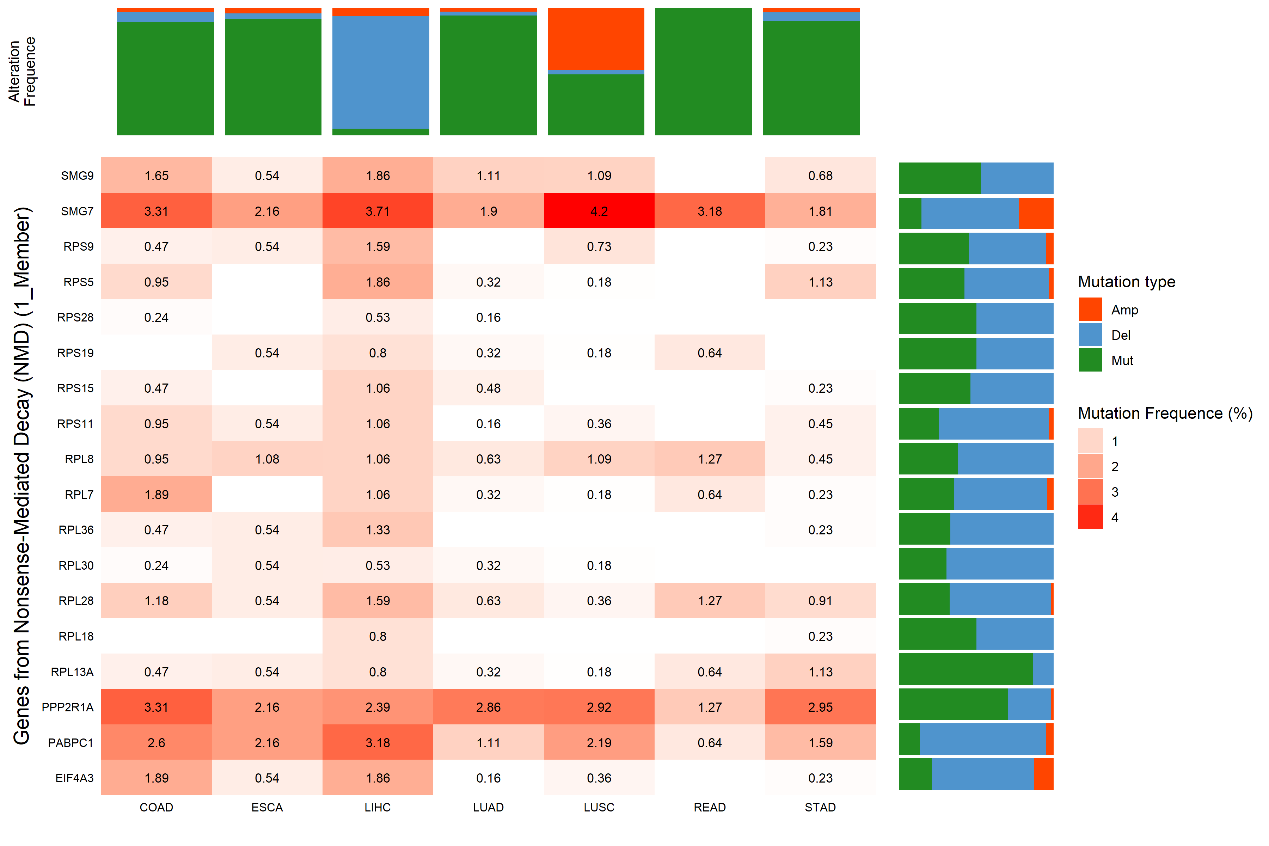


**Figure S13** (4) The copy number variances (CNVs) and mutation profile of genes in the key pathways


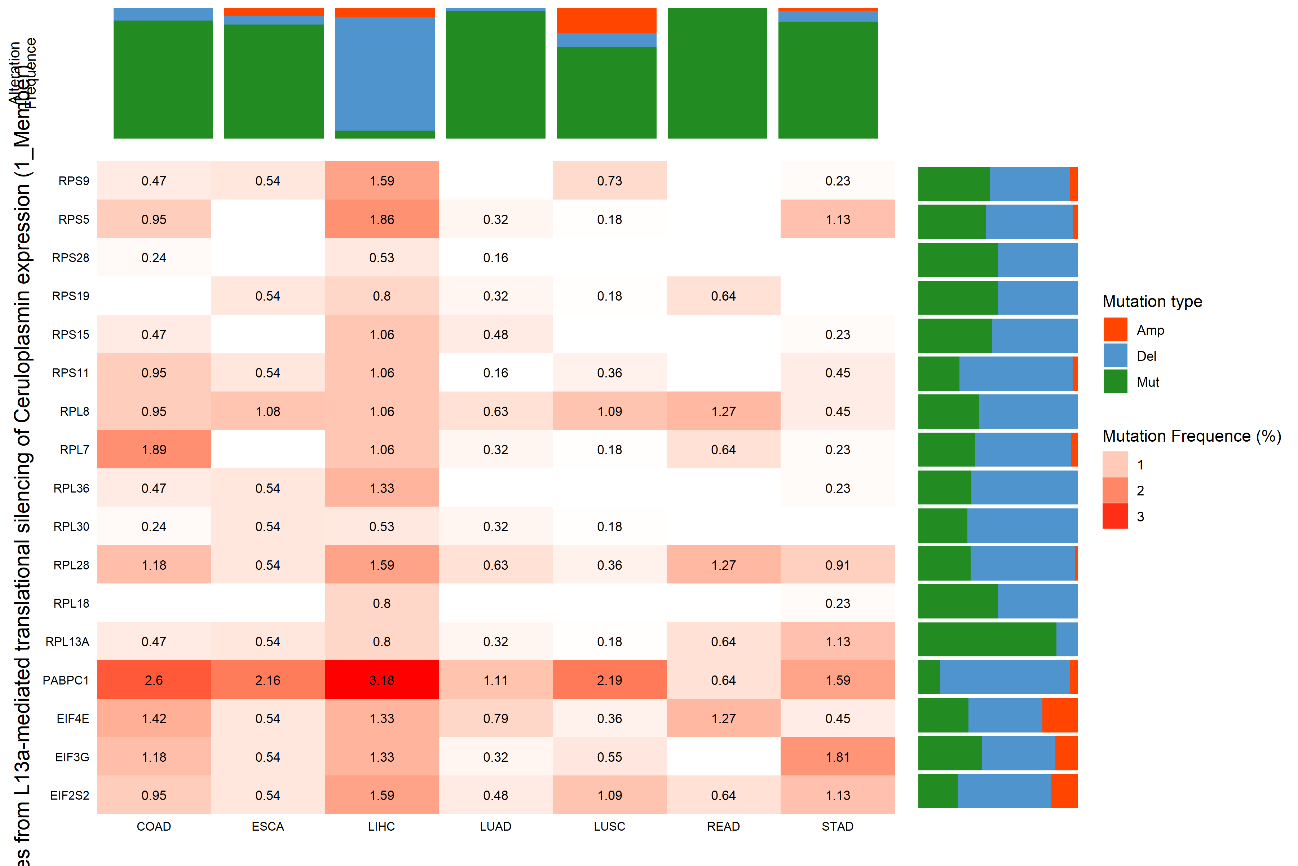


**Figure S13** (5) The copy number variances (CNVs) and mutation profile of genes in the key pathways
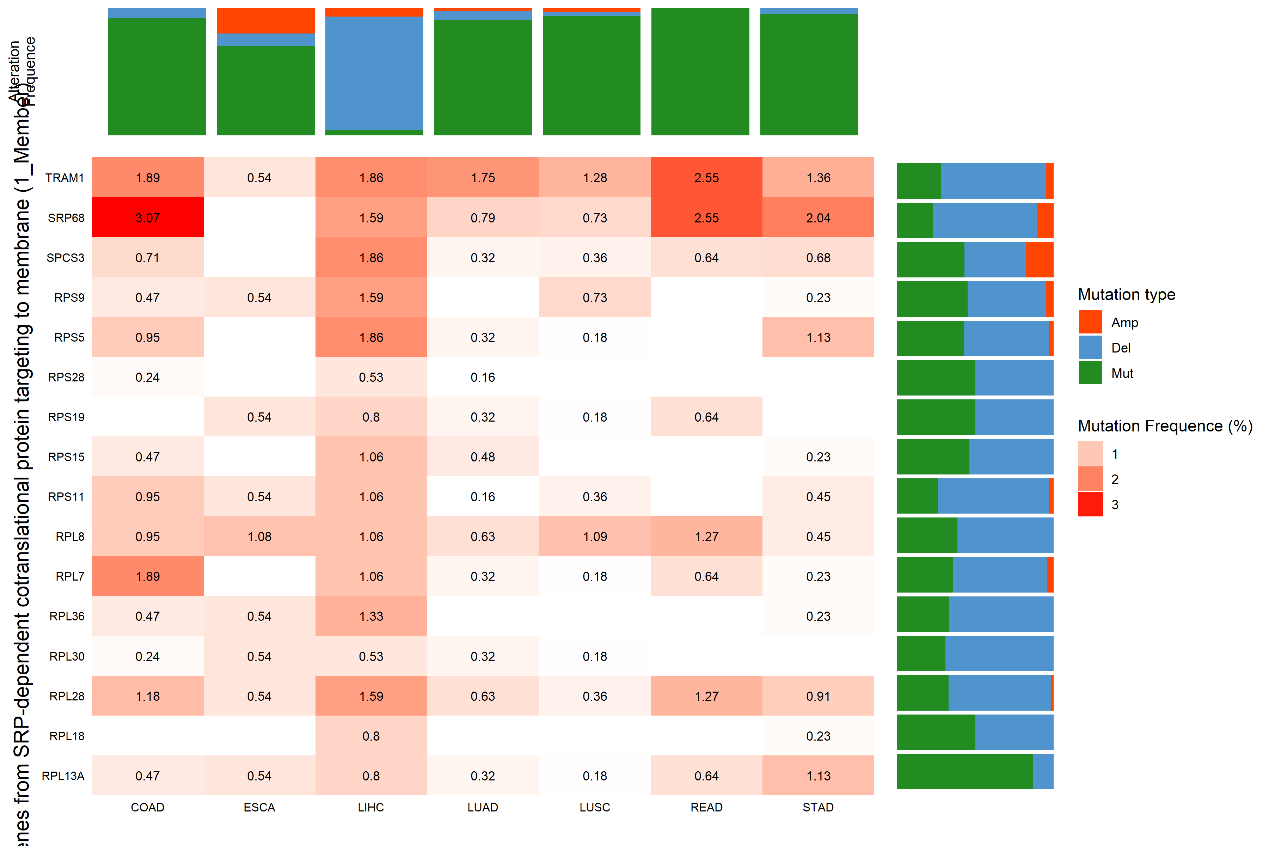
**Figure S13** (6) The copy number variances (CNVs) and mutation profile of genes in the key pathways


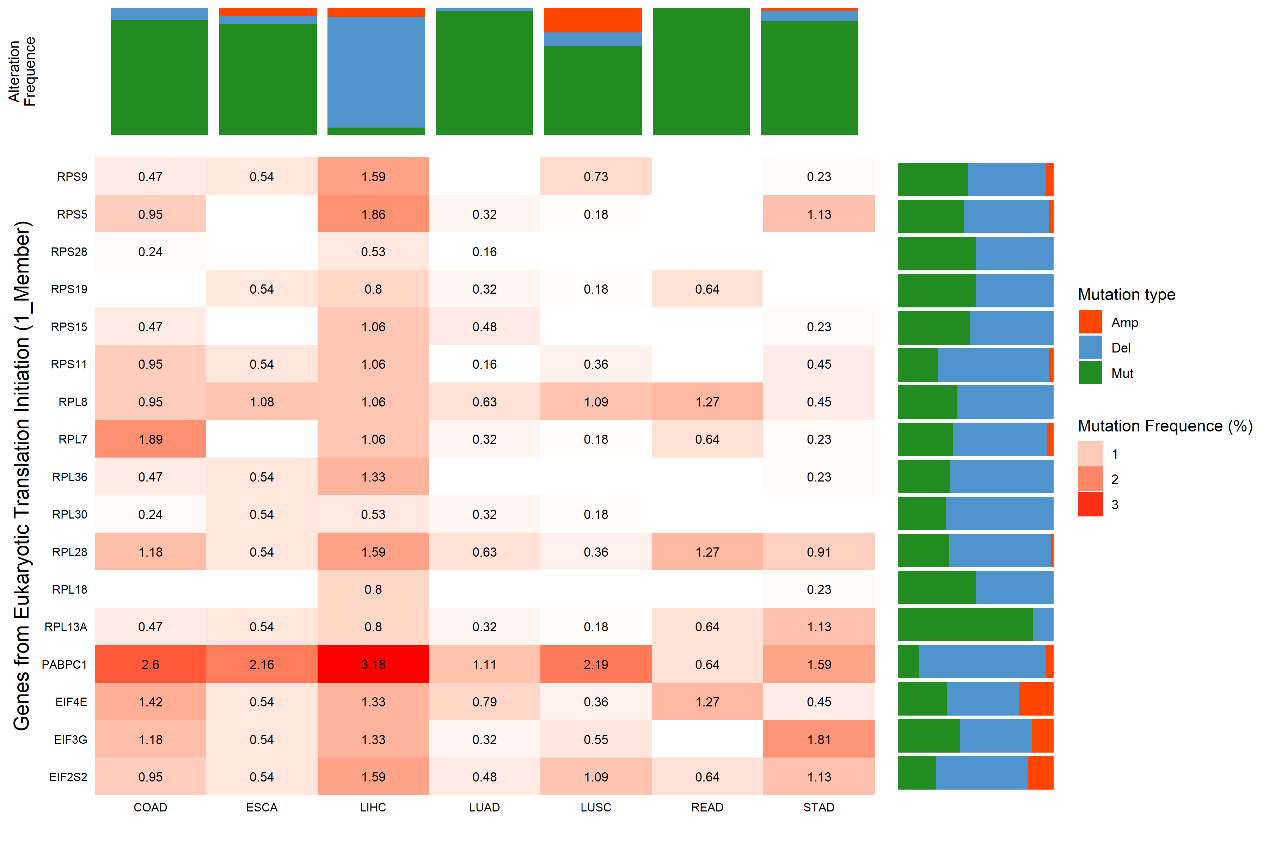


**Figure S13** (7) The copy number variances (CNVs) and mutation profile of genes in the key pathways


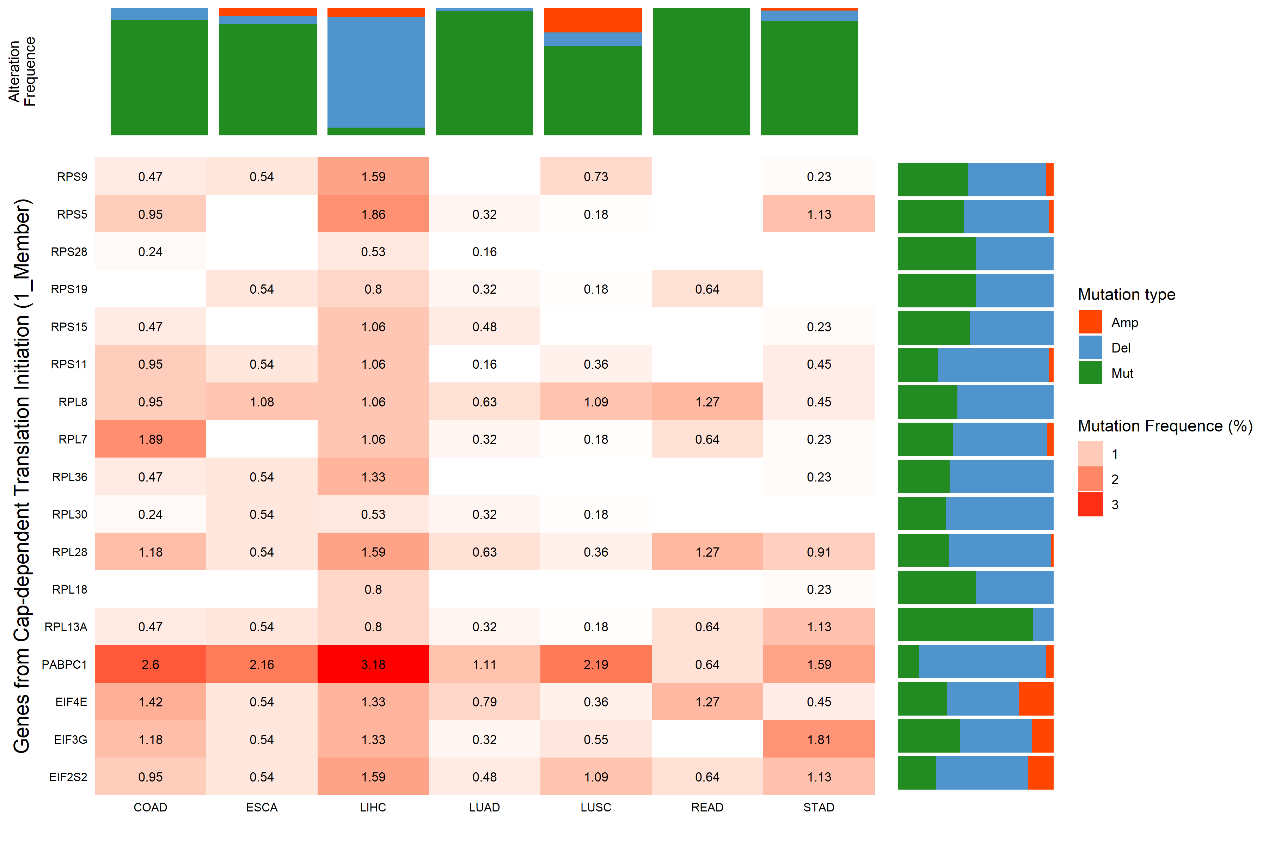


**Figure S13** (8) The copy number variances (CNVs) and mutation profile of genes in the key pathways


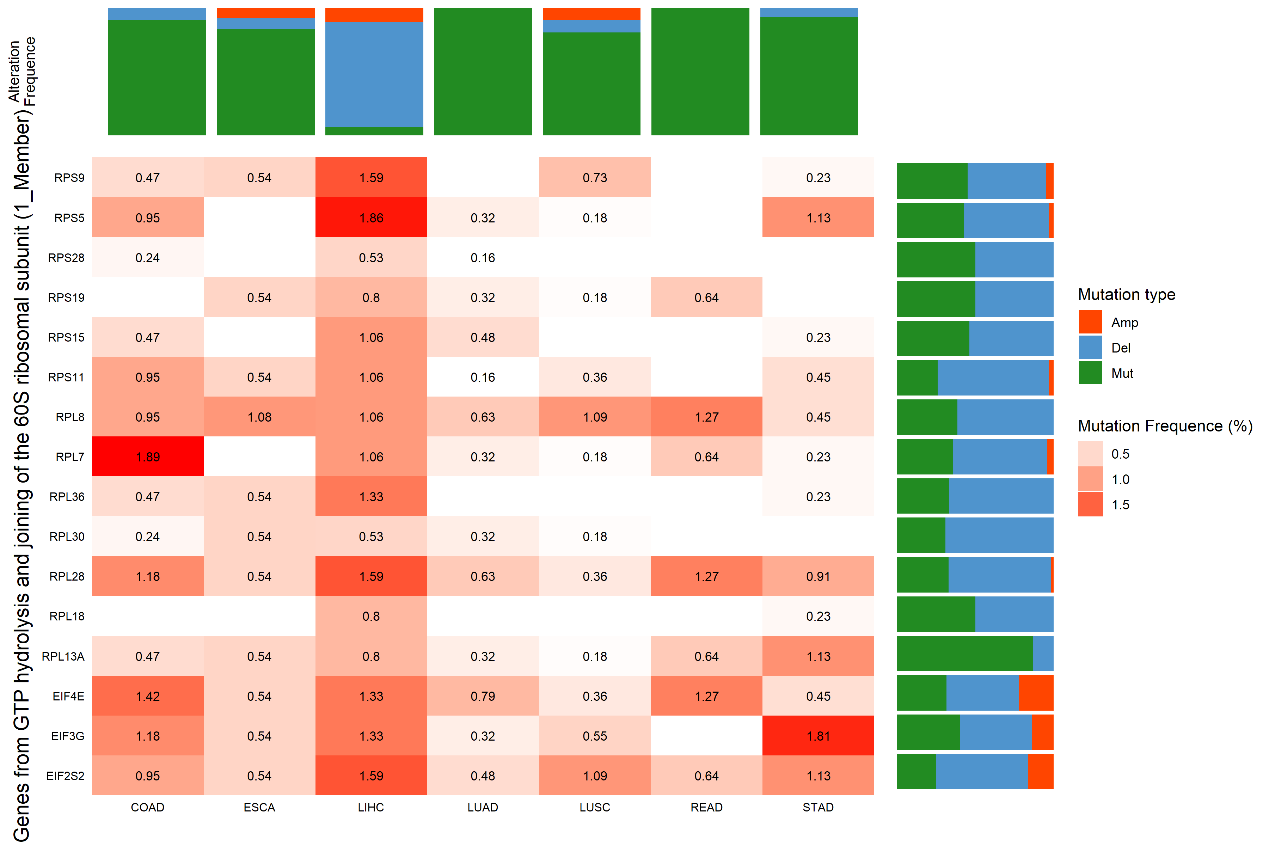


**Figure S13** (9) The copy number variances (CNVs) and mutation profile of genes in the key pathways


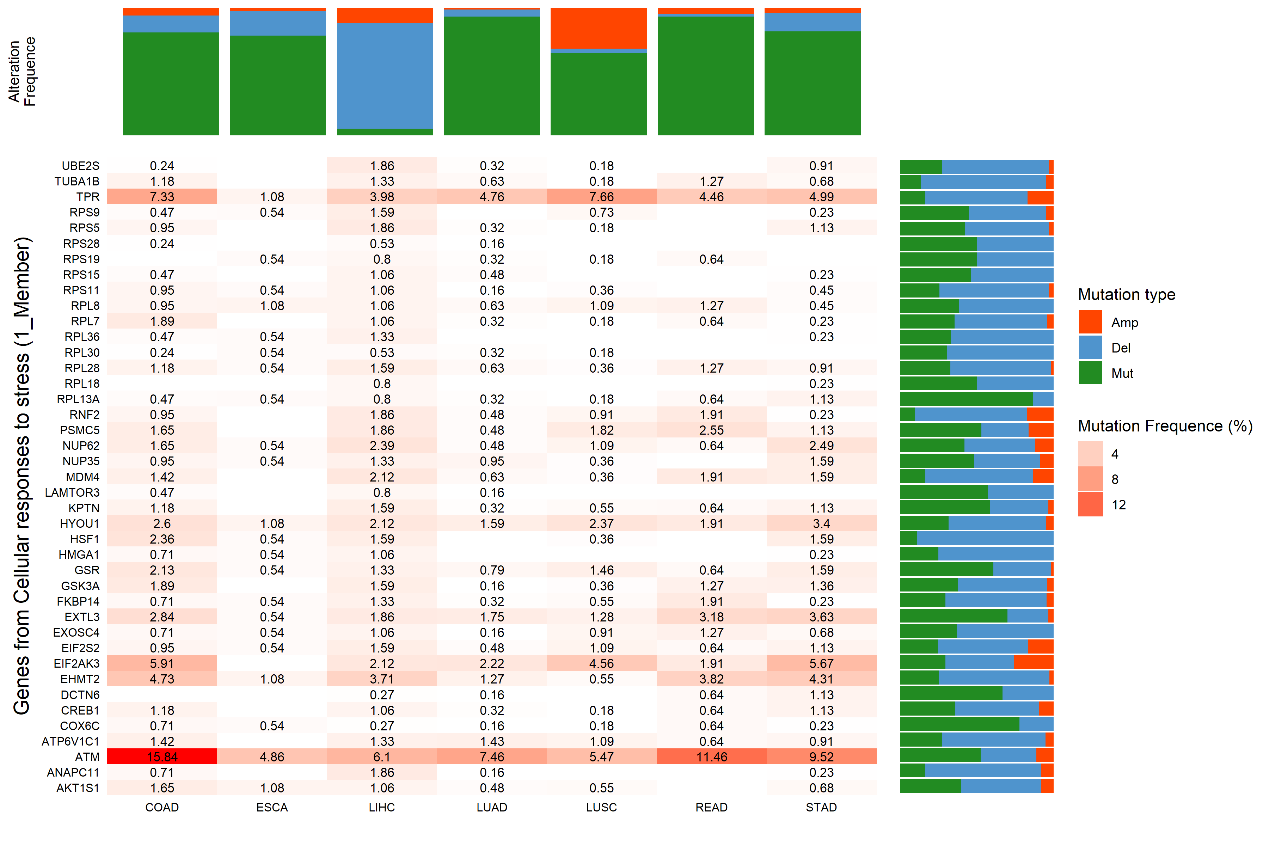


**Figure S13** (10) The copy number variances (CNVs) and mutation profile of genes in the key pathways


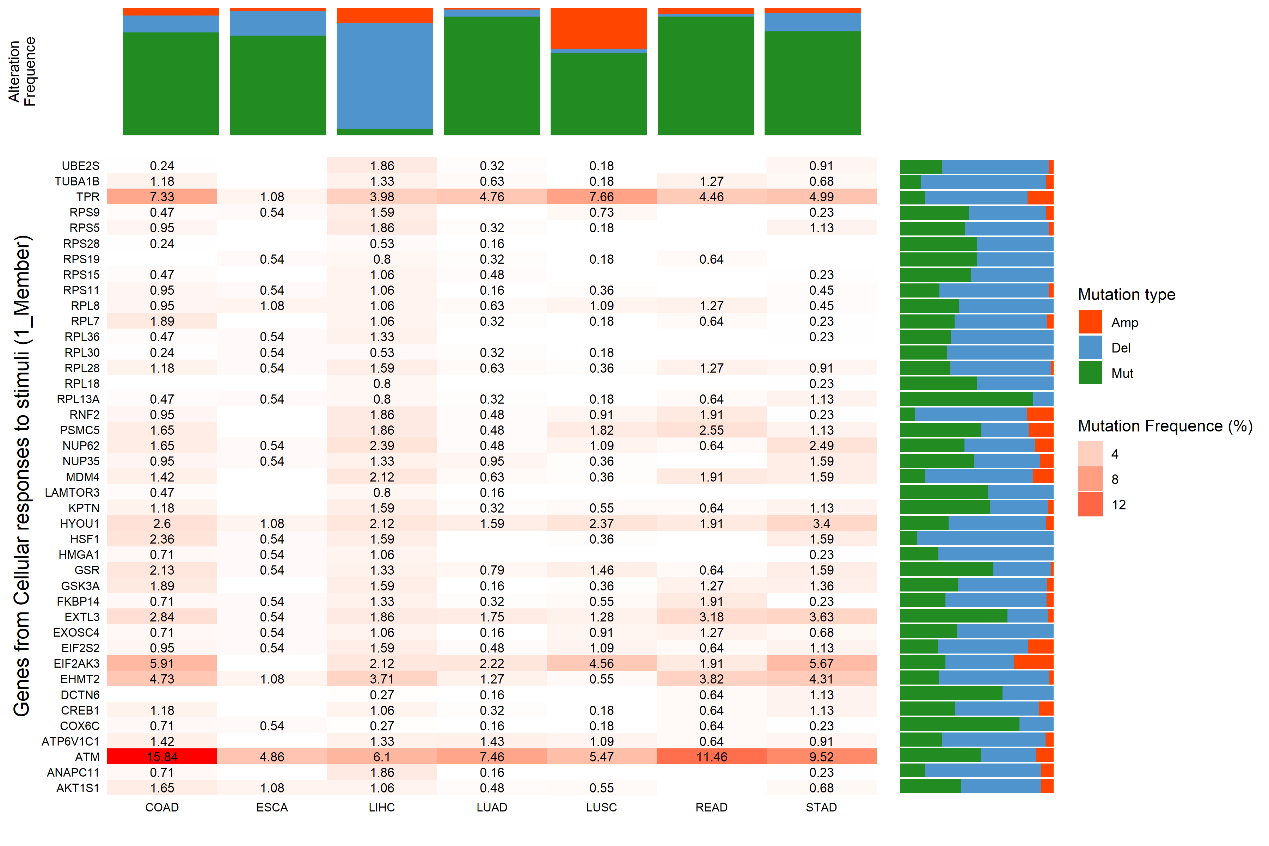


**Figure S13** (11) The copy number variances (CNVs) and mutation profile of genes in the key pathways


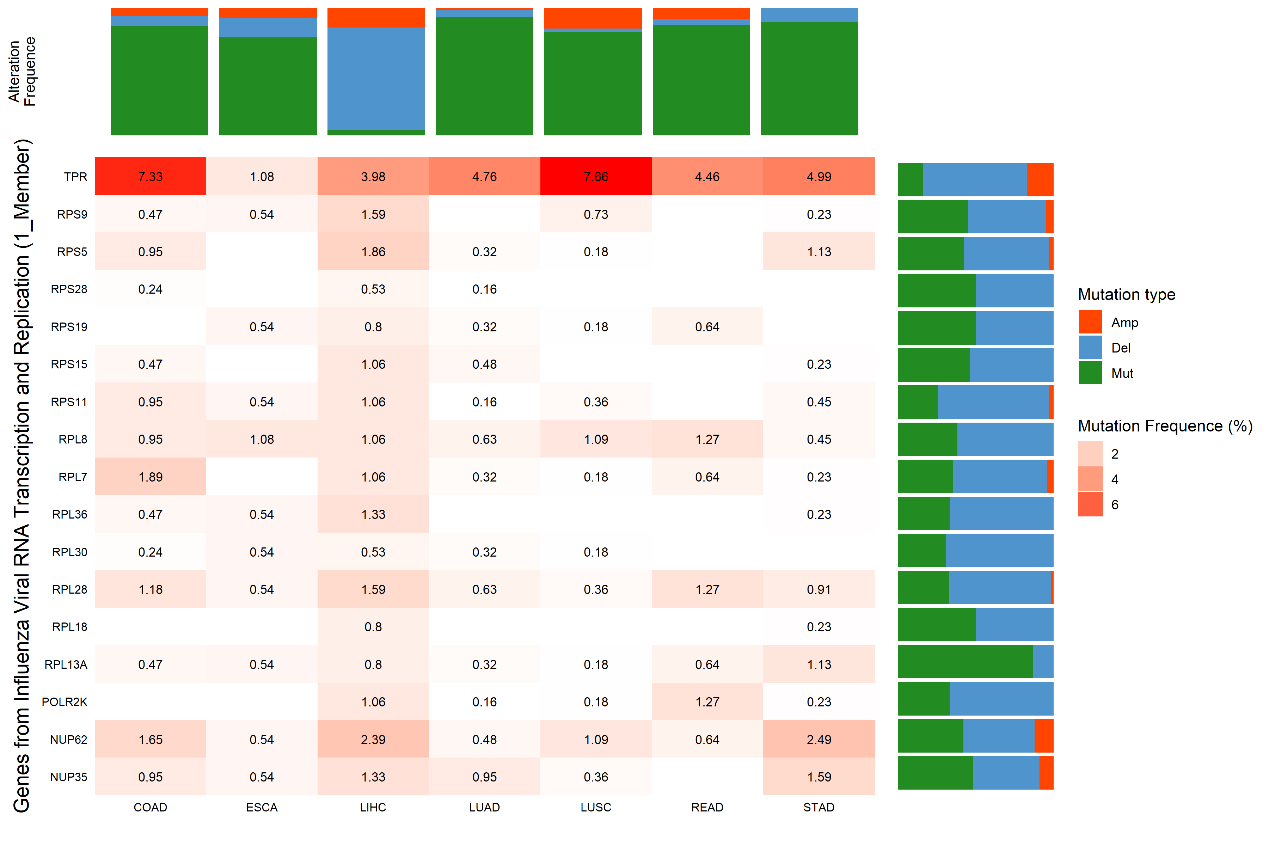


**Figure S13** (12) The copy number variances (CNVs) and mutation profile of genes in the key pathways


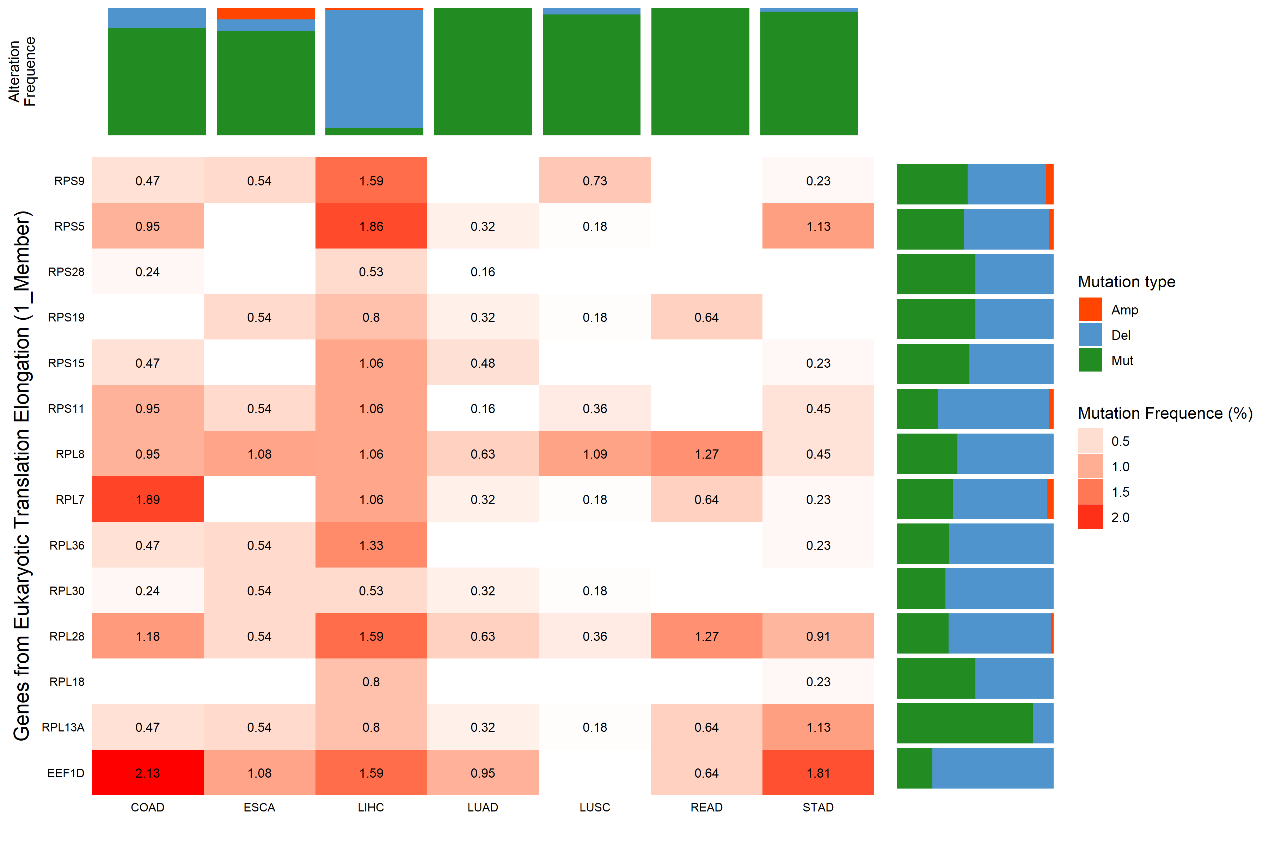


**Figure S13** (13) The copy number variances (CNVs) and mutation profile of genes in the key pathways


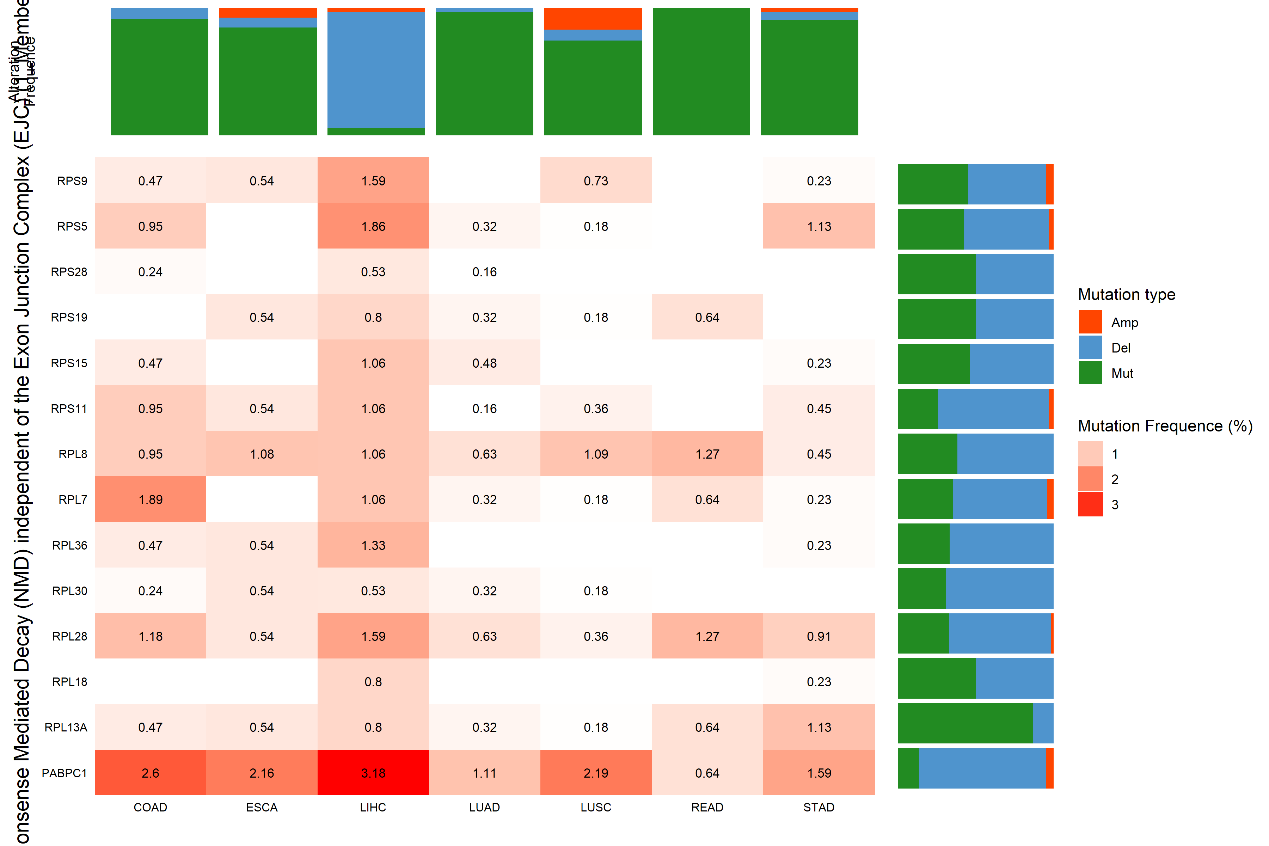


**Figure S13** (14) The copy number variances (CNVs) and mutation profile of genes in the key pathways


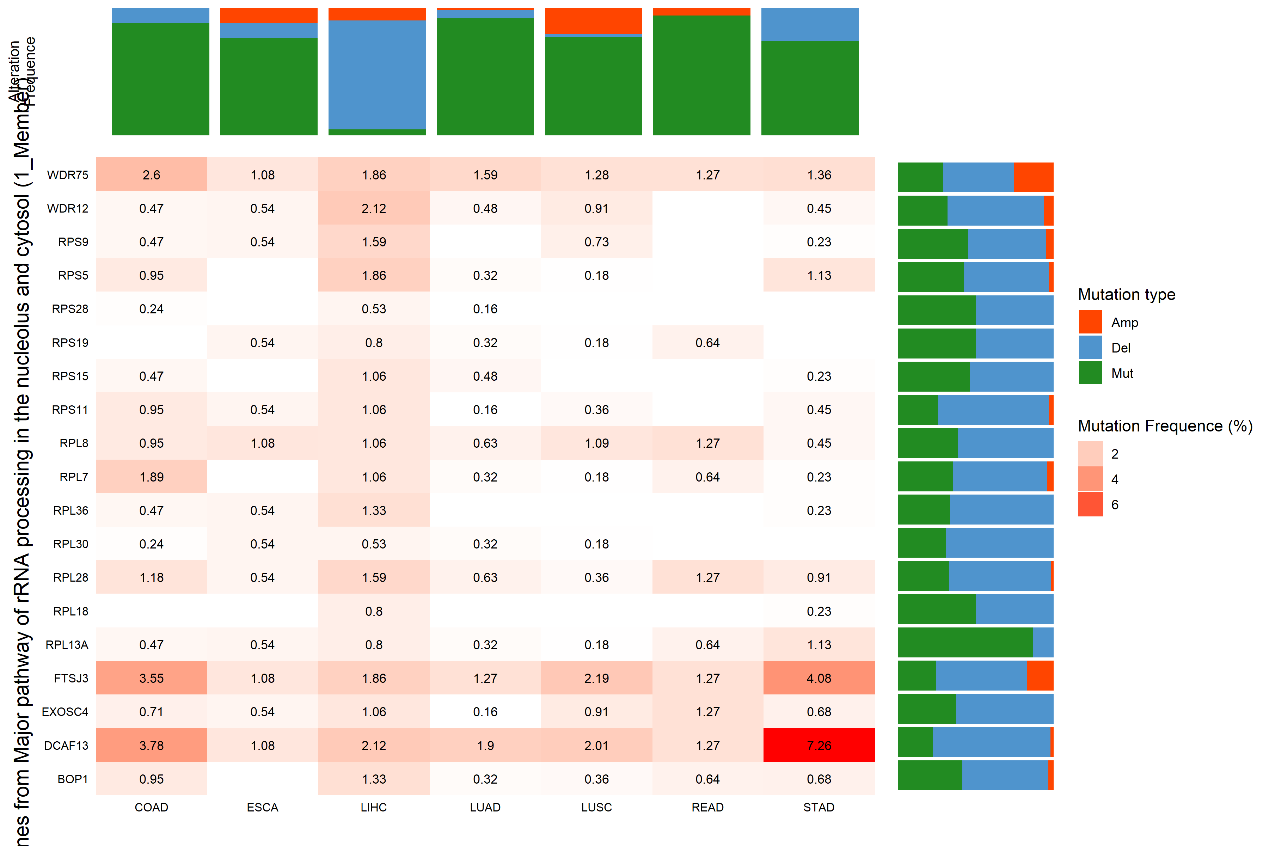


**Figure S13** (15) The copy number variances (CNVs) and mutation profile of genes in the key pathways


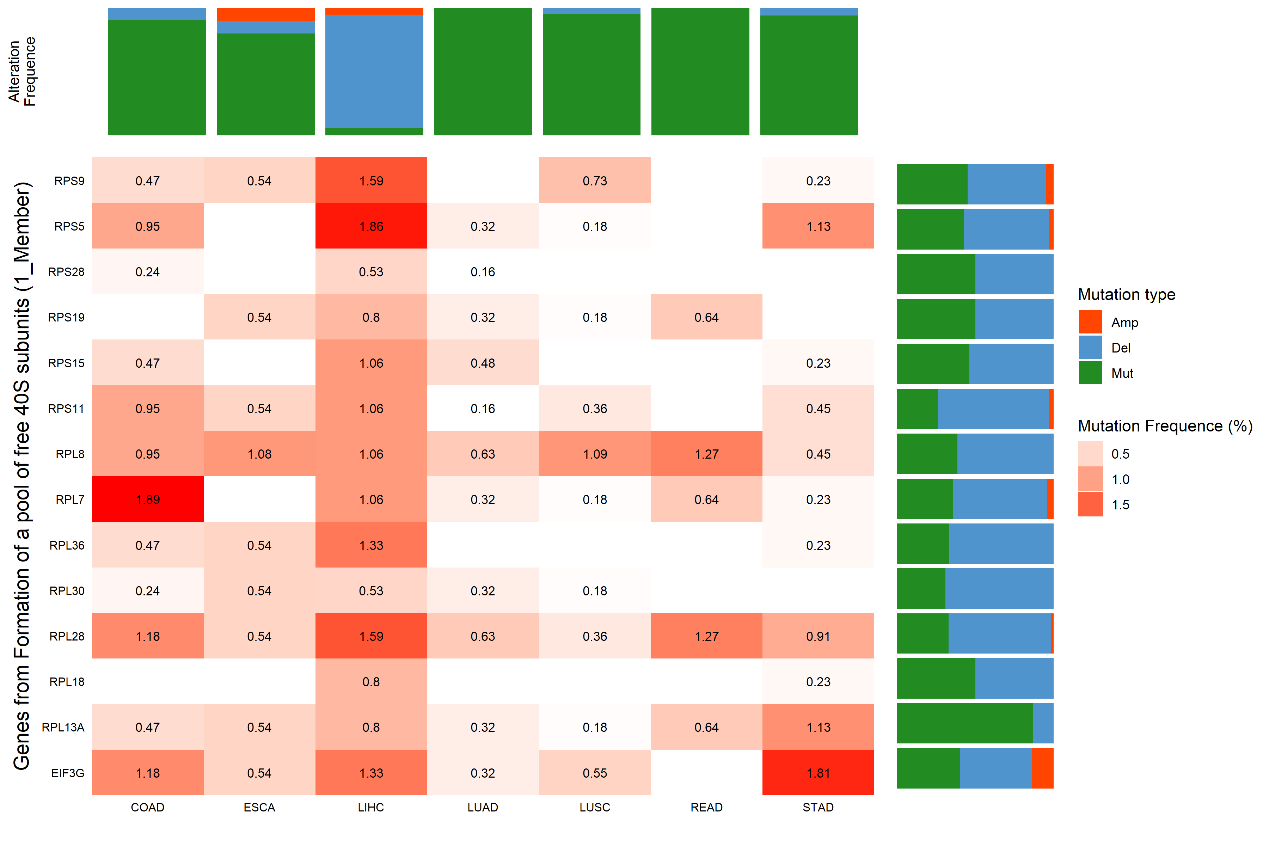


**Figure S13** (16) The copy number variances (CNVs) and mutation profile of genes in the key pathways


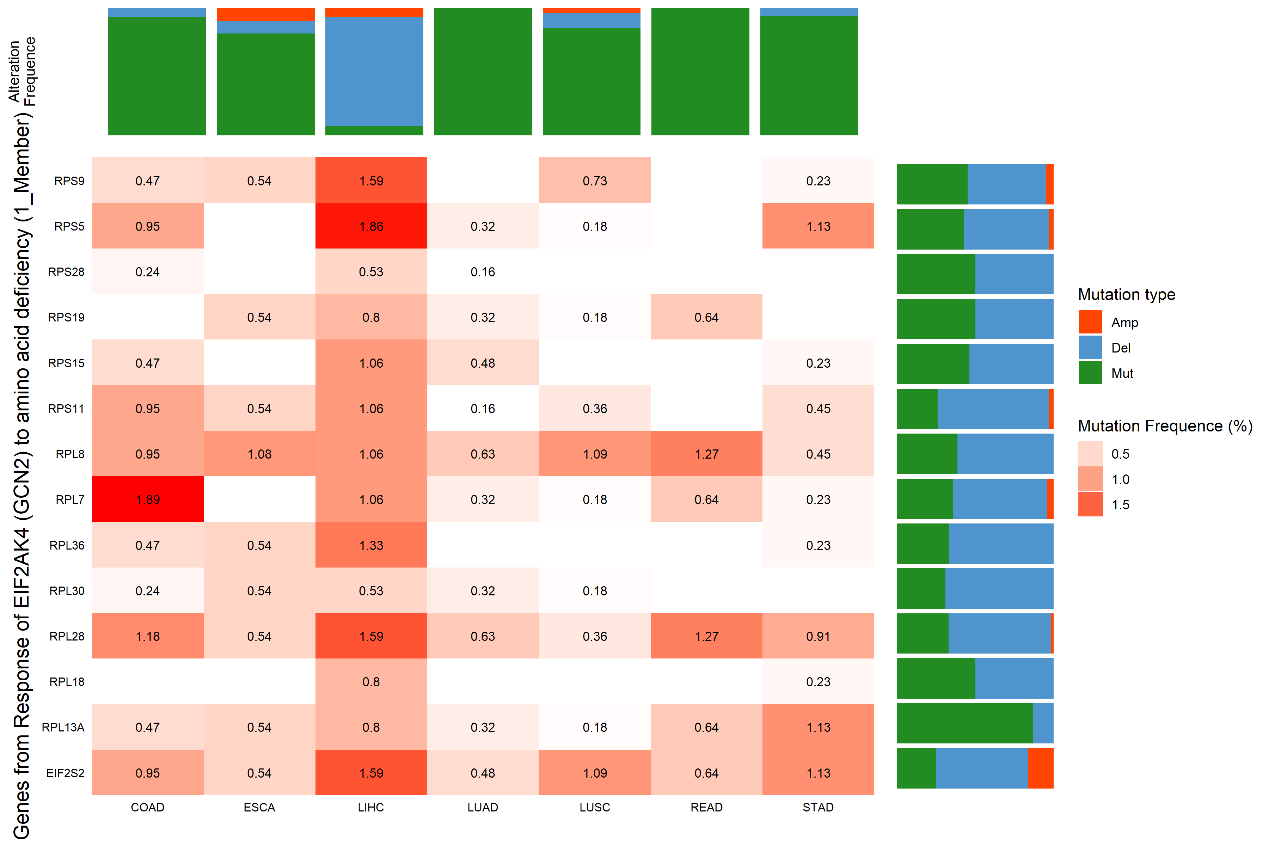


**Figure S13** (17) The copy number variances (CNVs) and mutation profile of genes in the key pathways
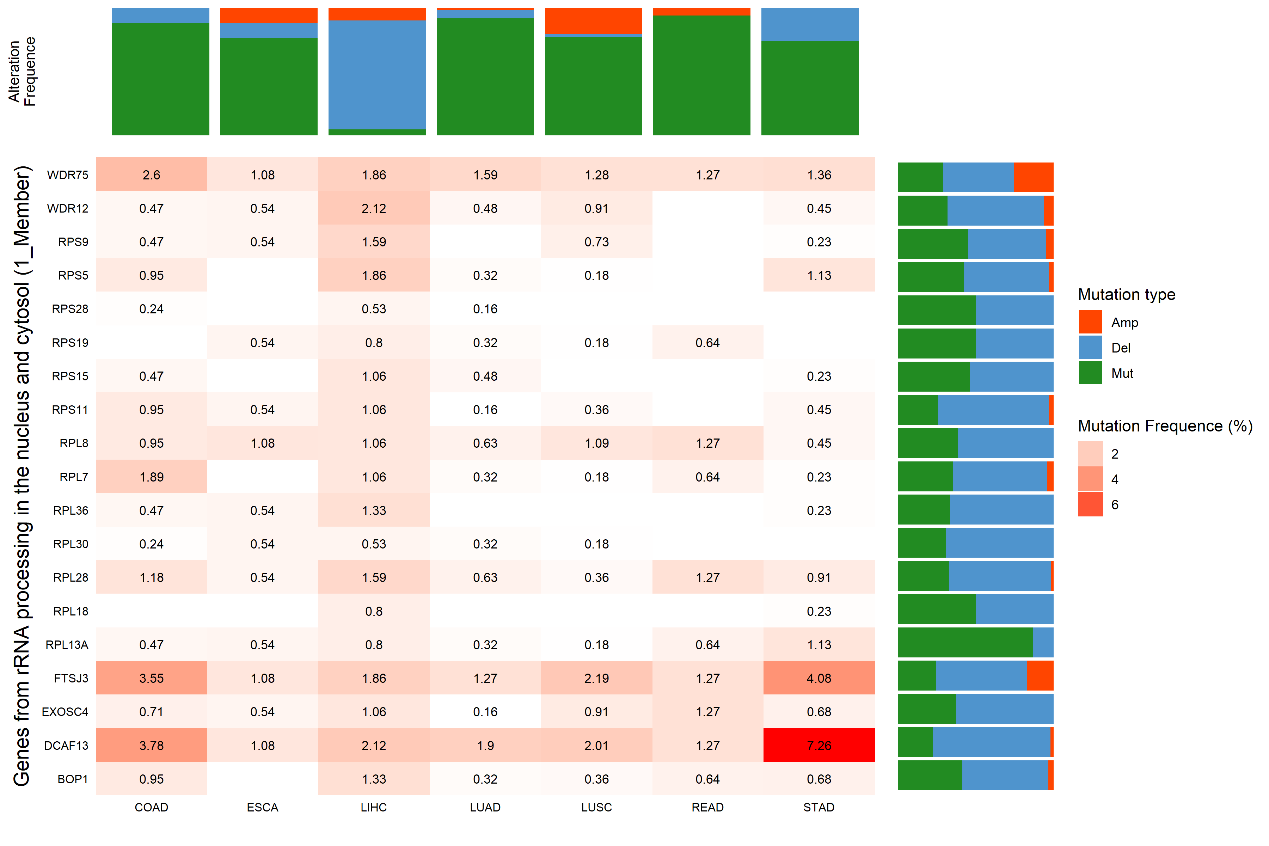


**Figure S13** (18) The copy number variances (CNVs) and mutation profile of genes in the key pathways


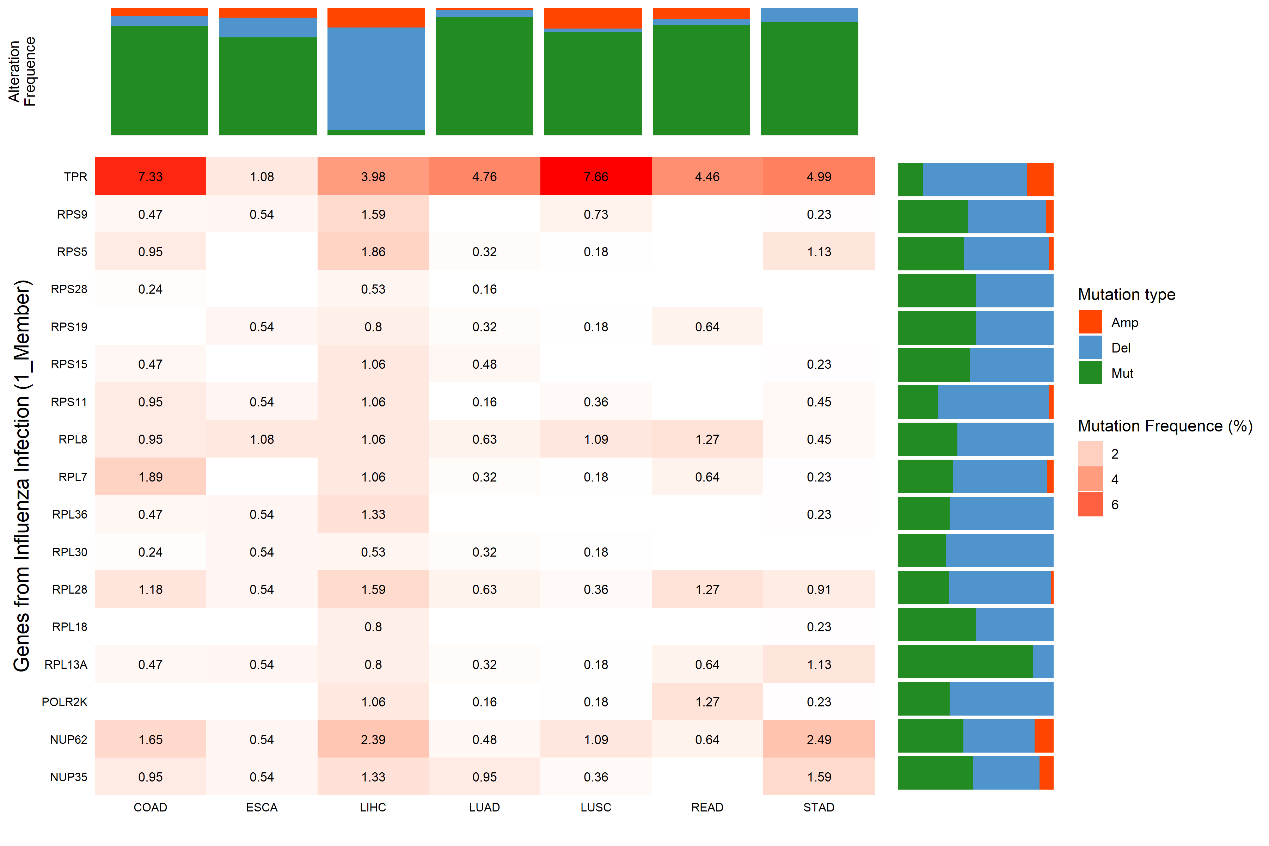


**Figure S13** (19) The copy number variances (CNVs) and mutation profile of genes in the key pathways


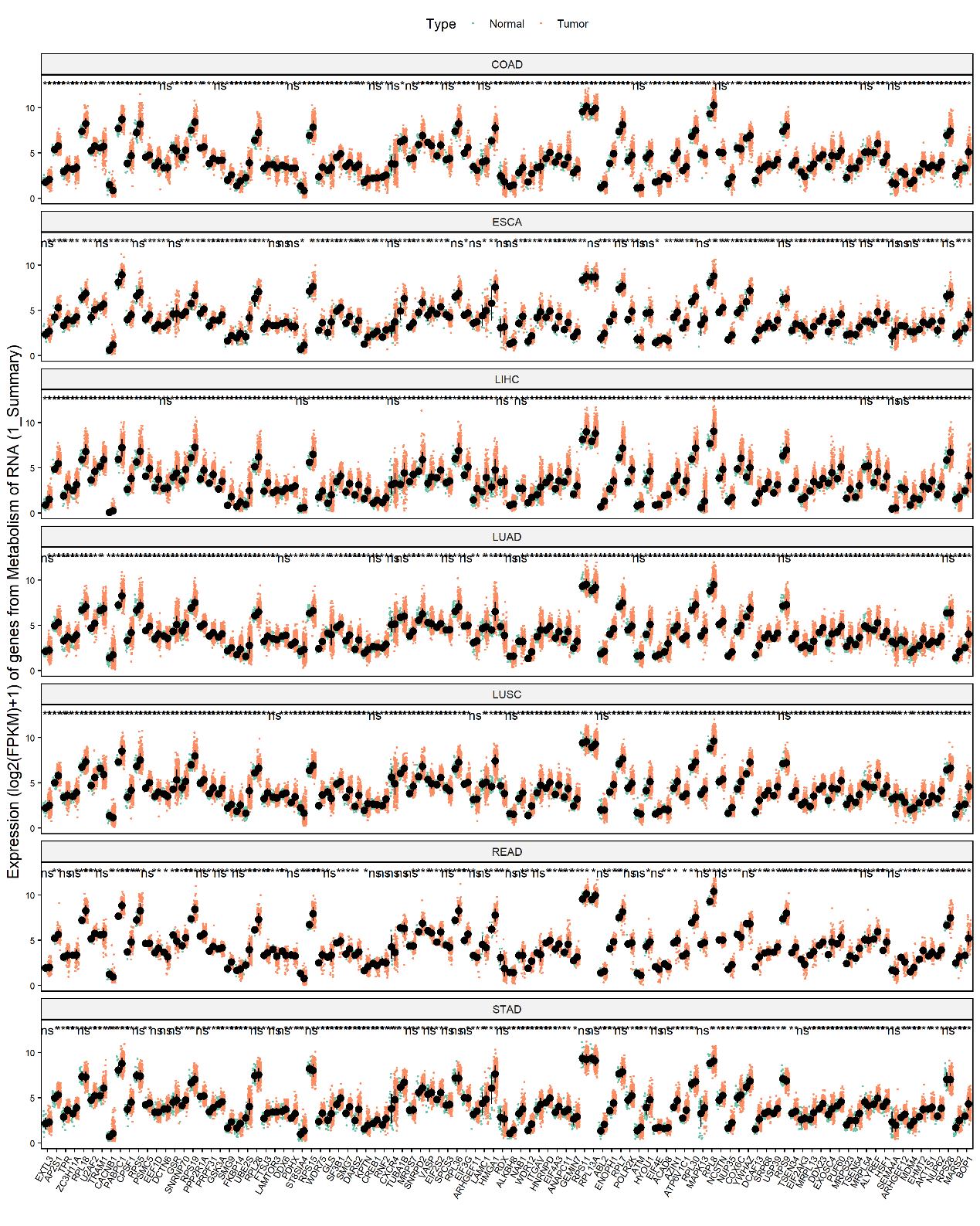


**Figure S14** (1) The expression profile of key genes in the key pathways


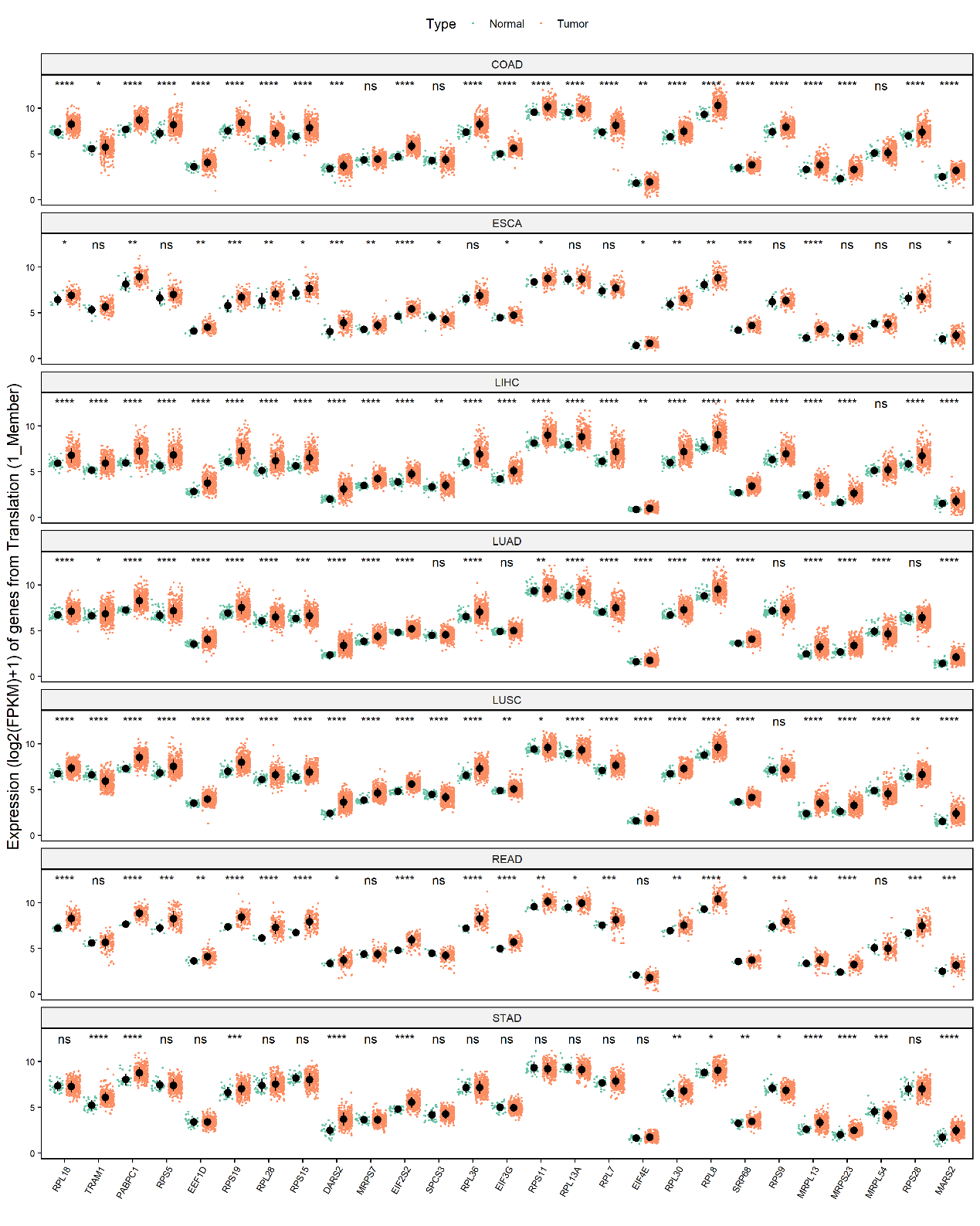


**Figure S14** (2) The expression profile of key genes in the key pathways


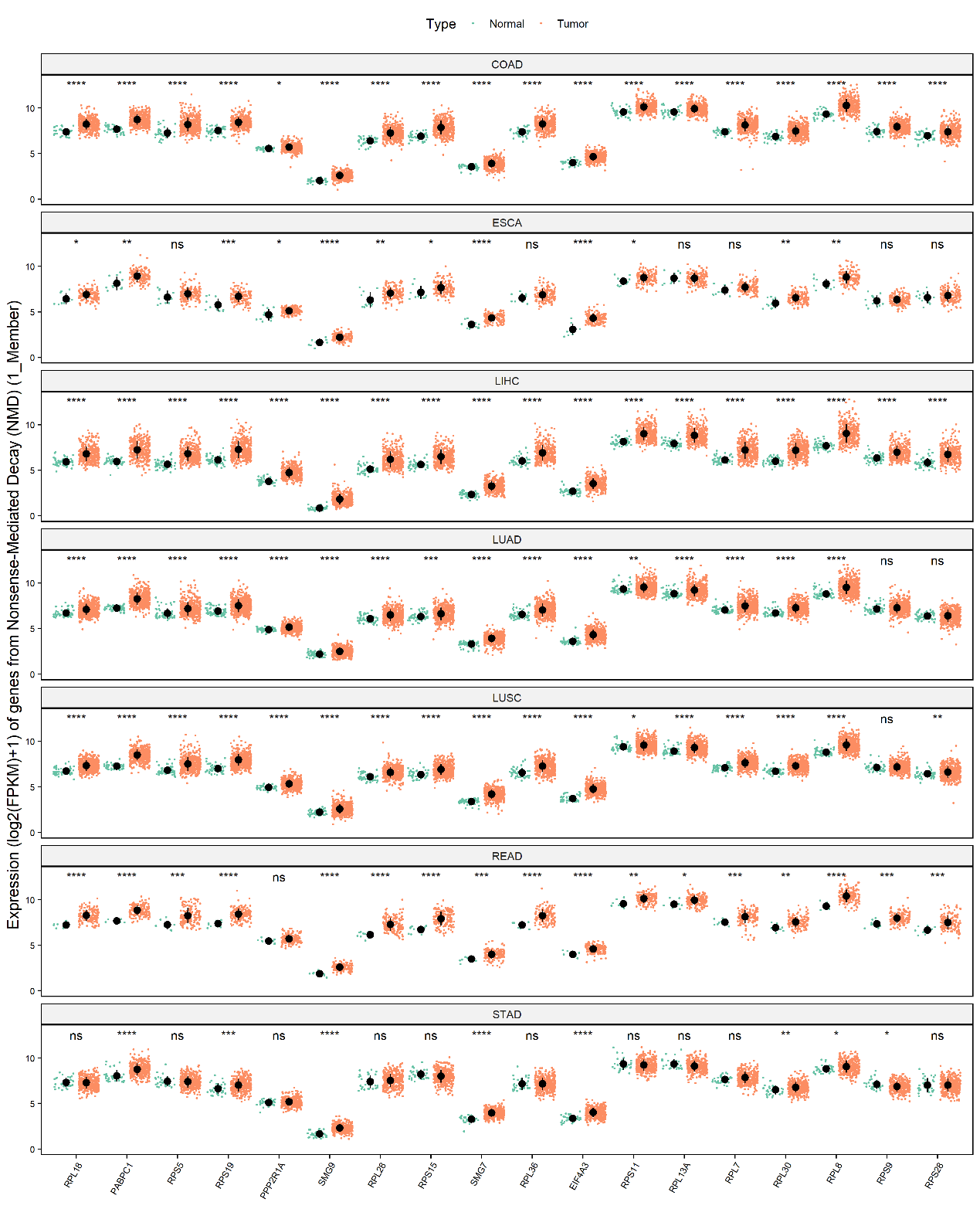


**Figure S14** (3) The expression profile of key genes in the key pathways


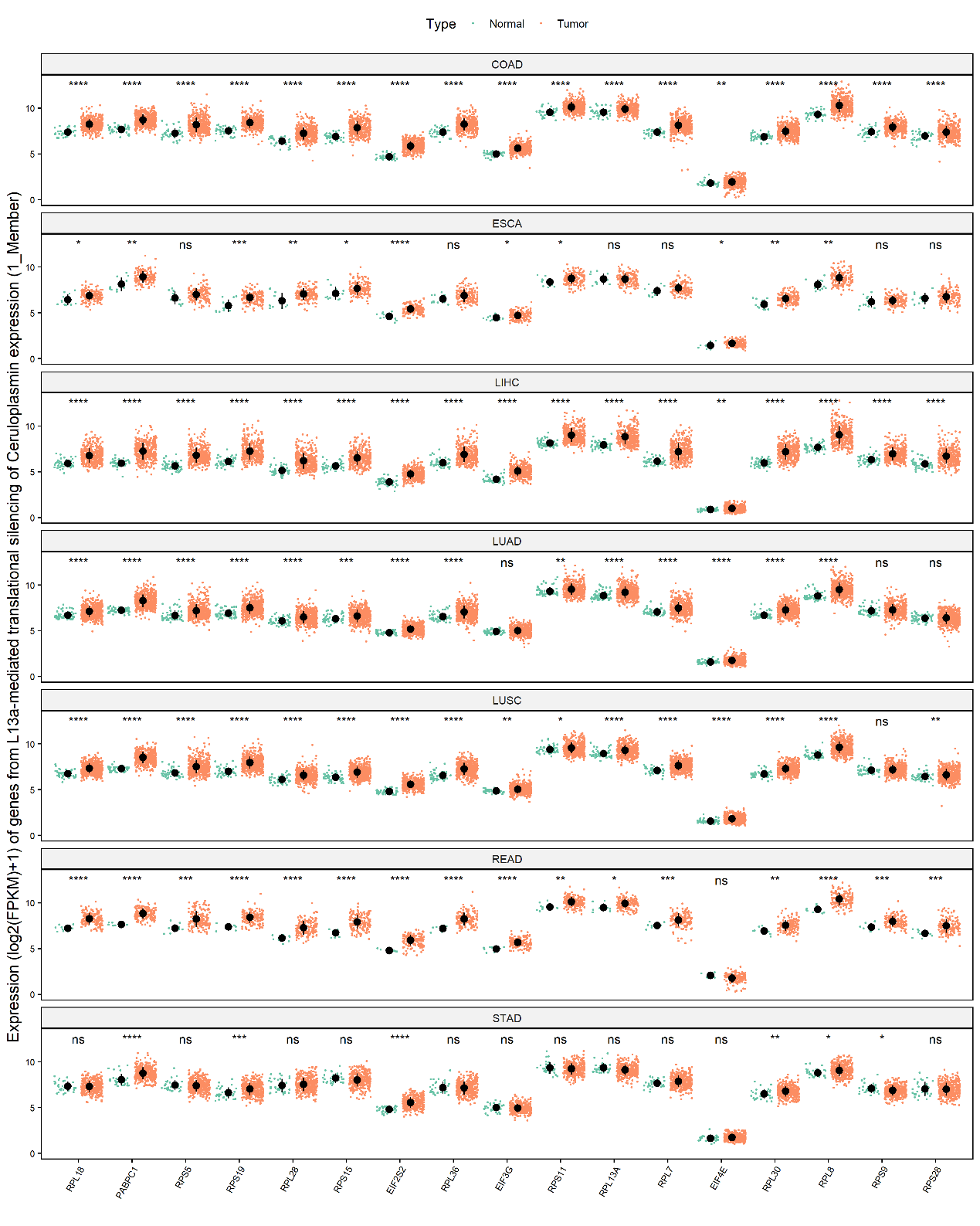


**Figure S14** (4) The expression profile of key genes in the key pathways


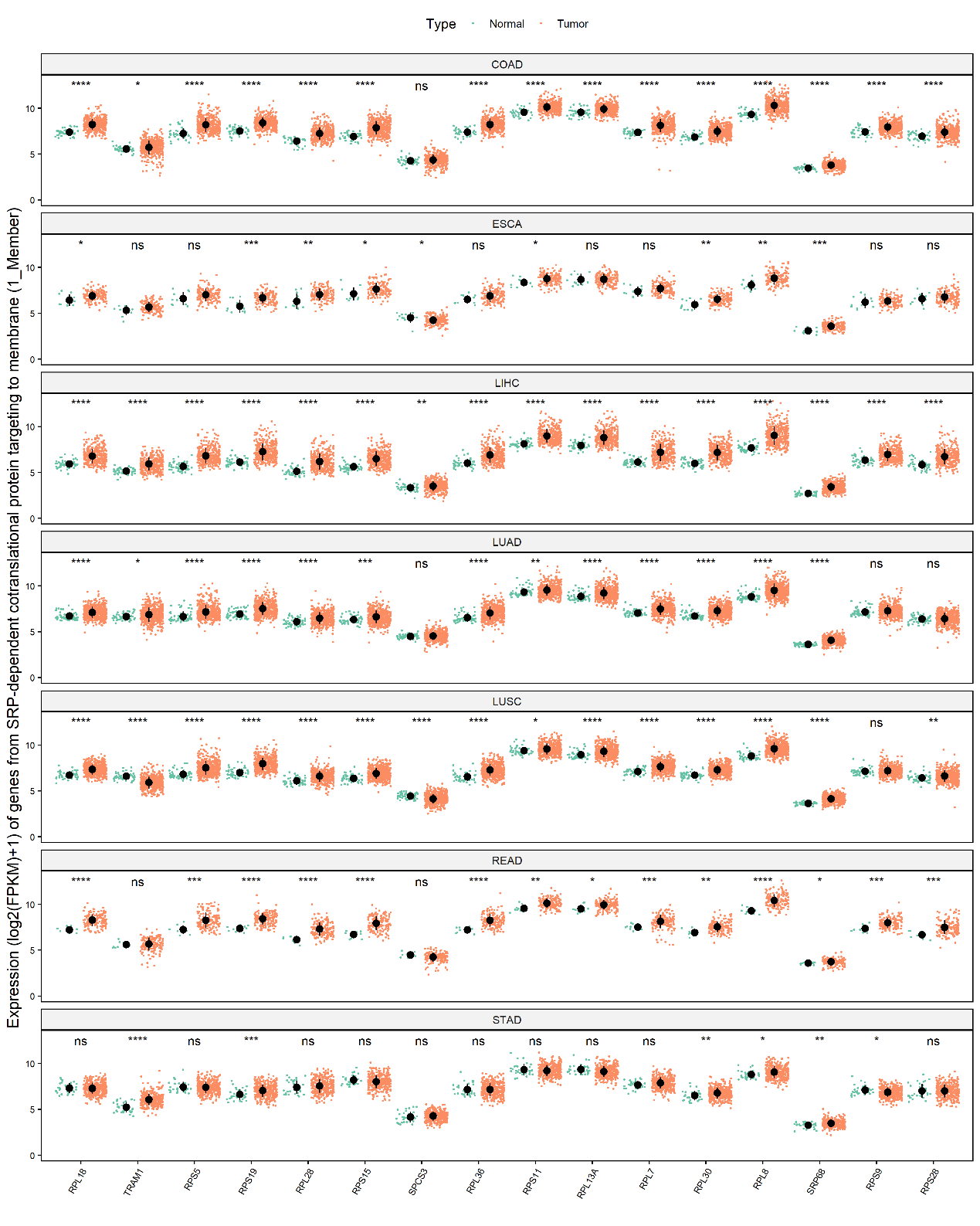


**Figure S14** (5) The expression profile of key genes in the key pathways


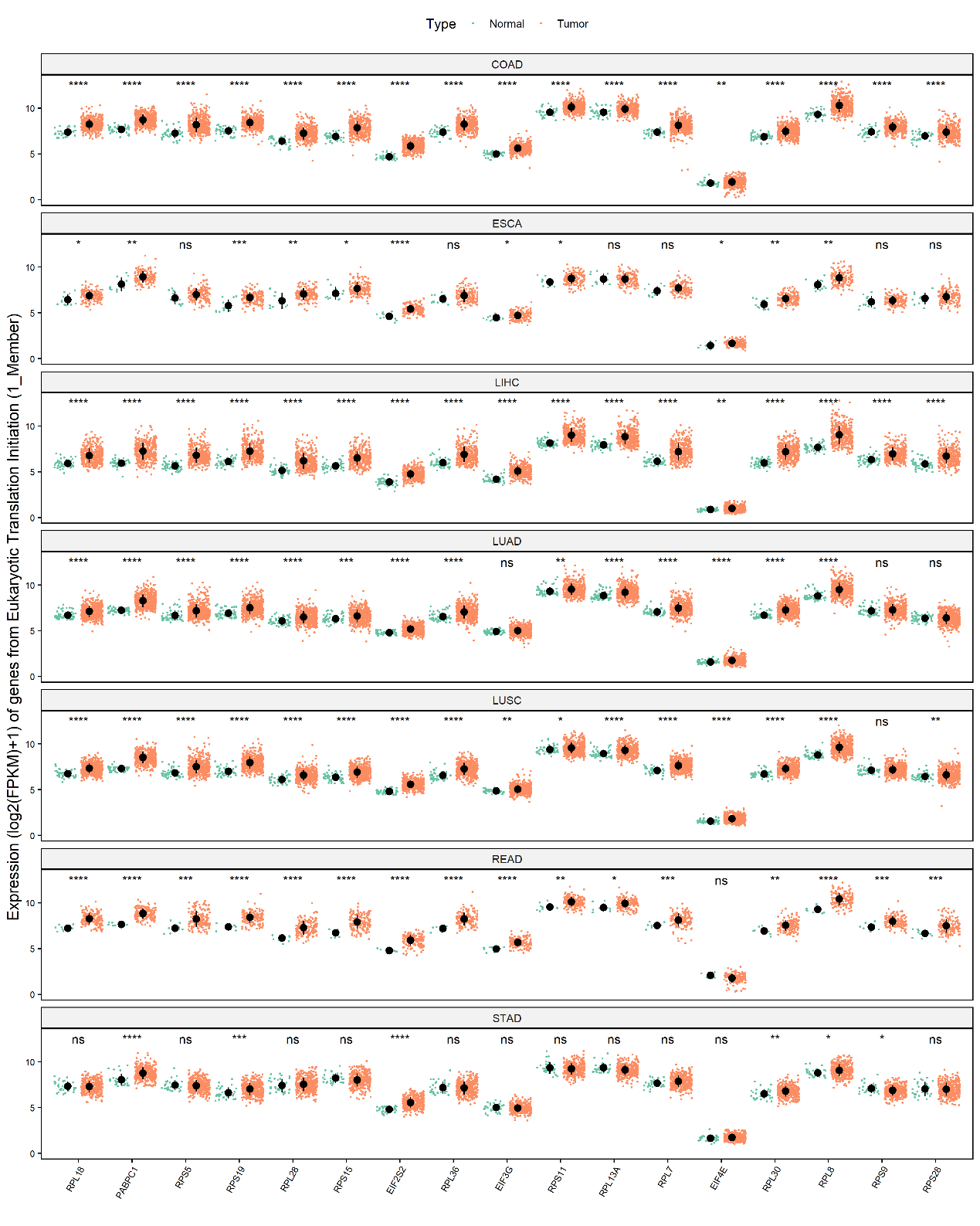


**Figure S14** (6) The expression profile of key genes in the key pathways


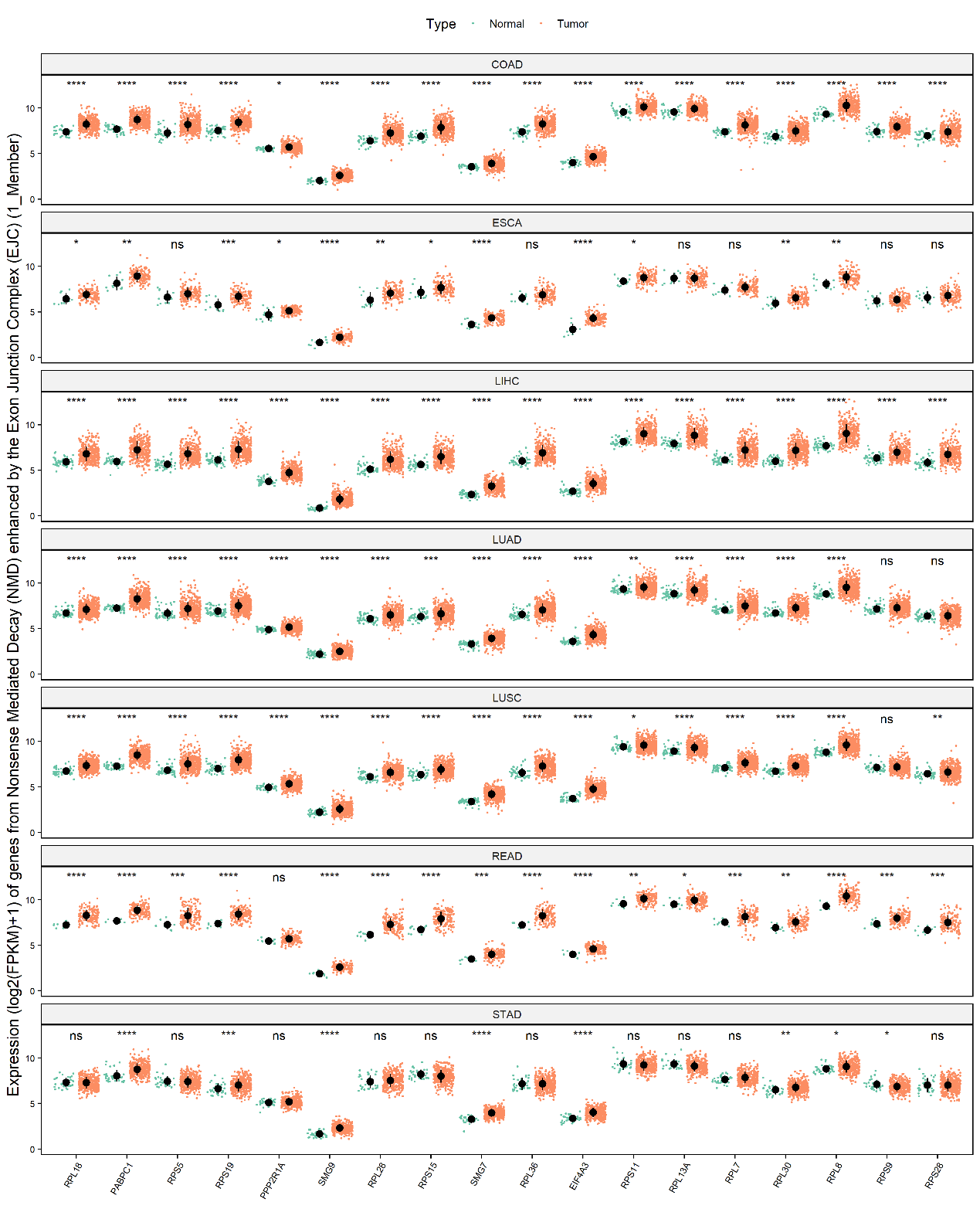


**Figure S14** (7) The expression profile of key genes in the key pathways


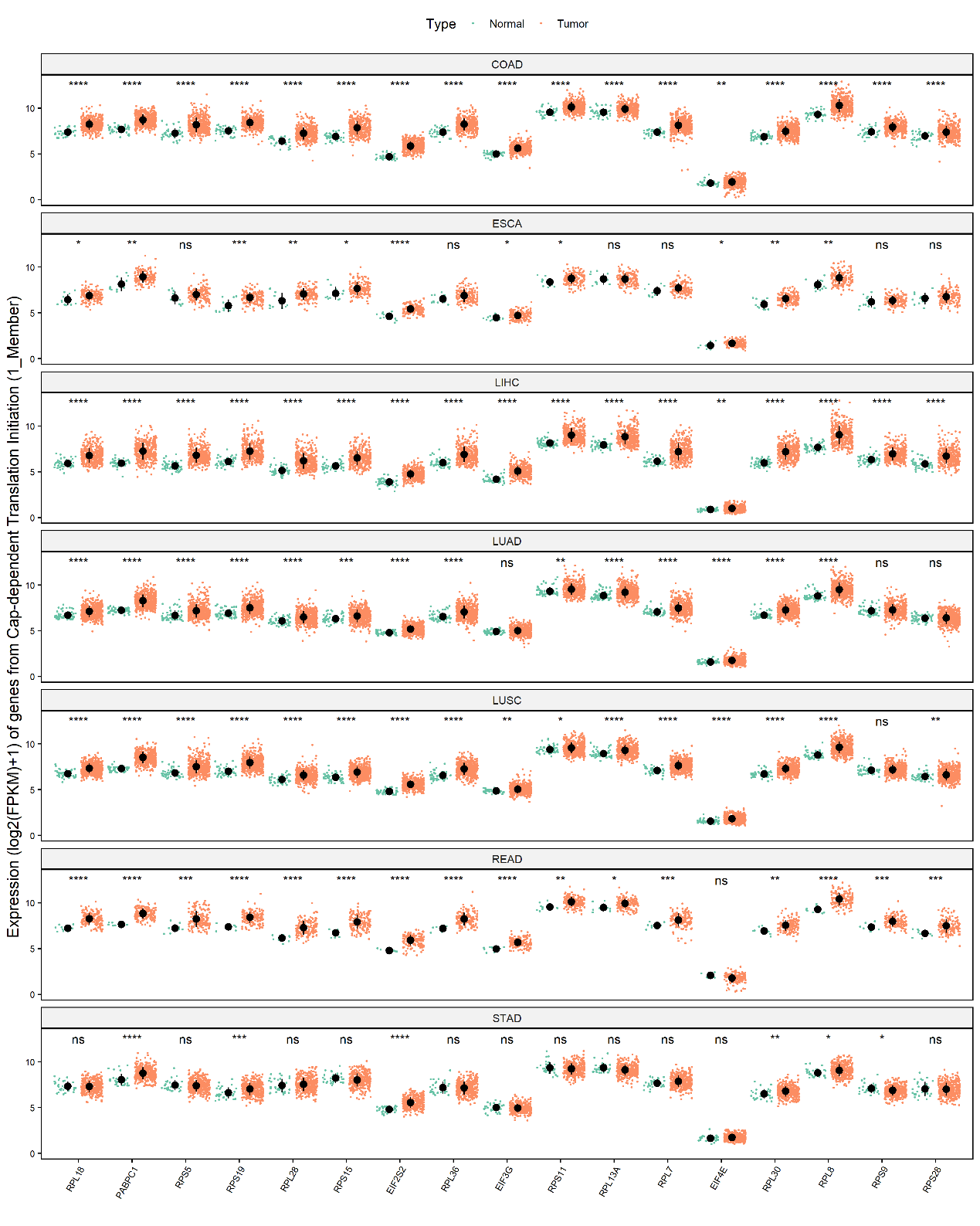


**Figure S14** (8) The expression profile of key genes in the key pathways


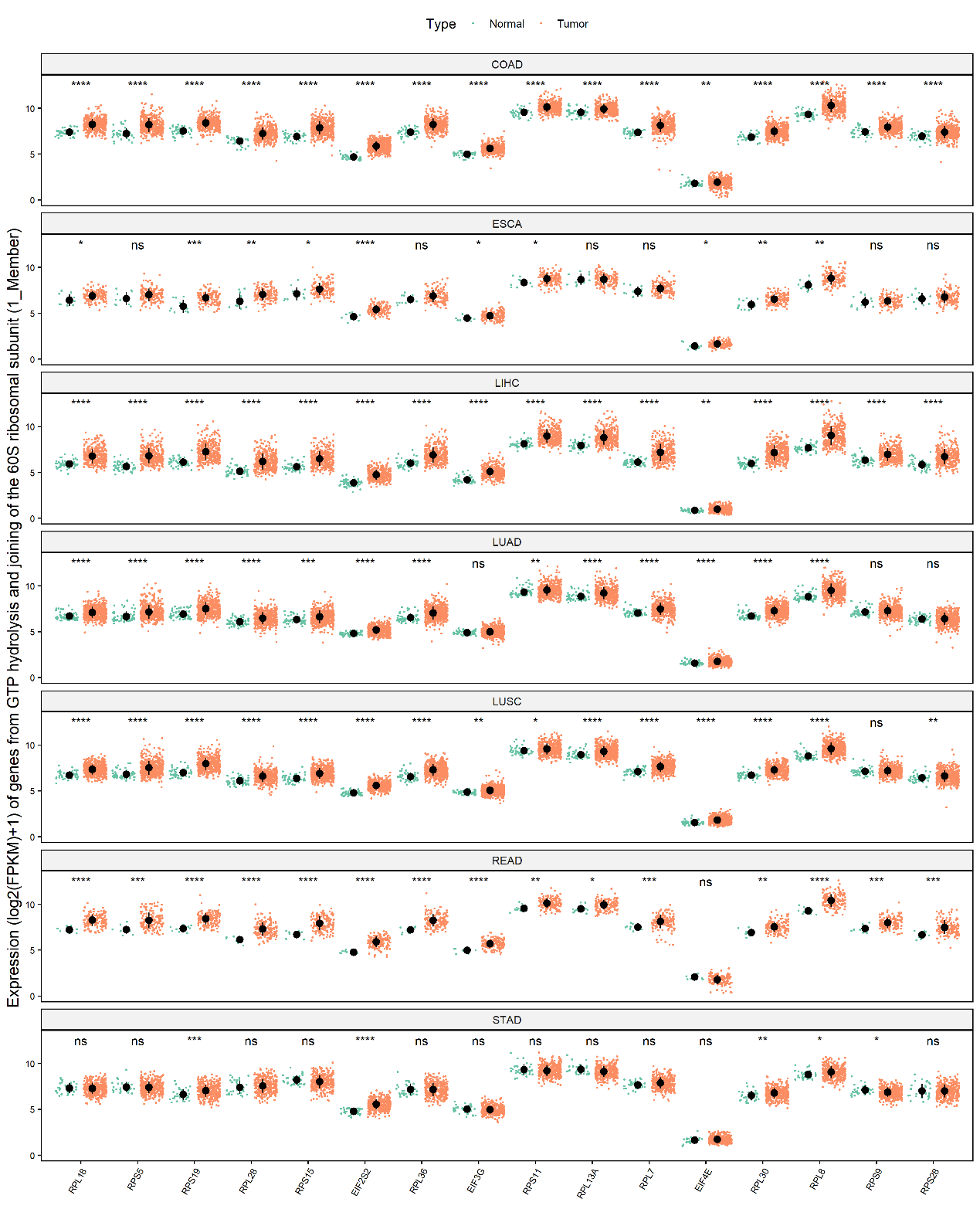


**Figure S14** (9) The expression profile of key genes in the key pathways


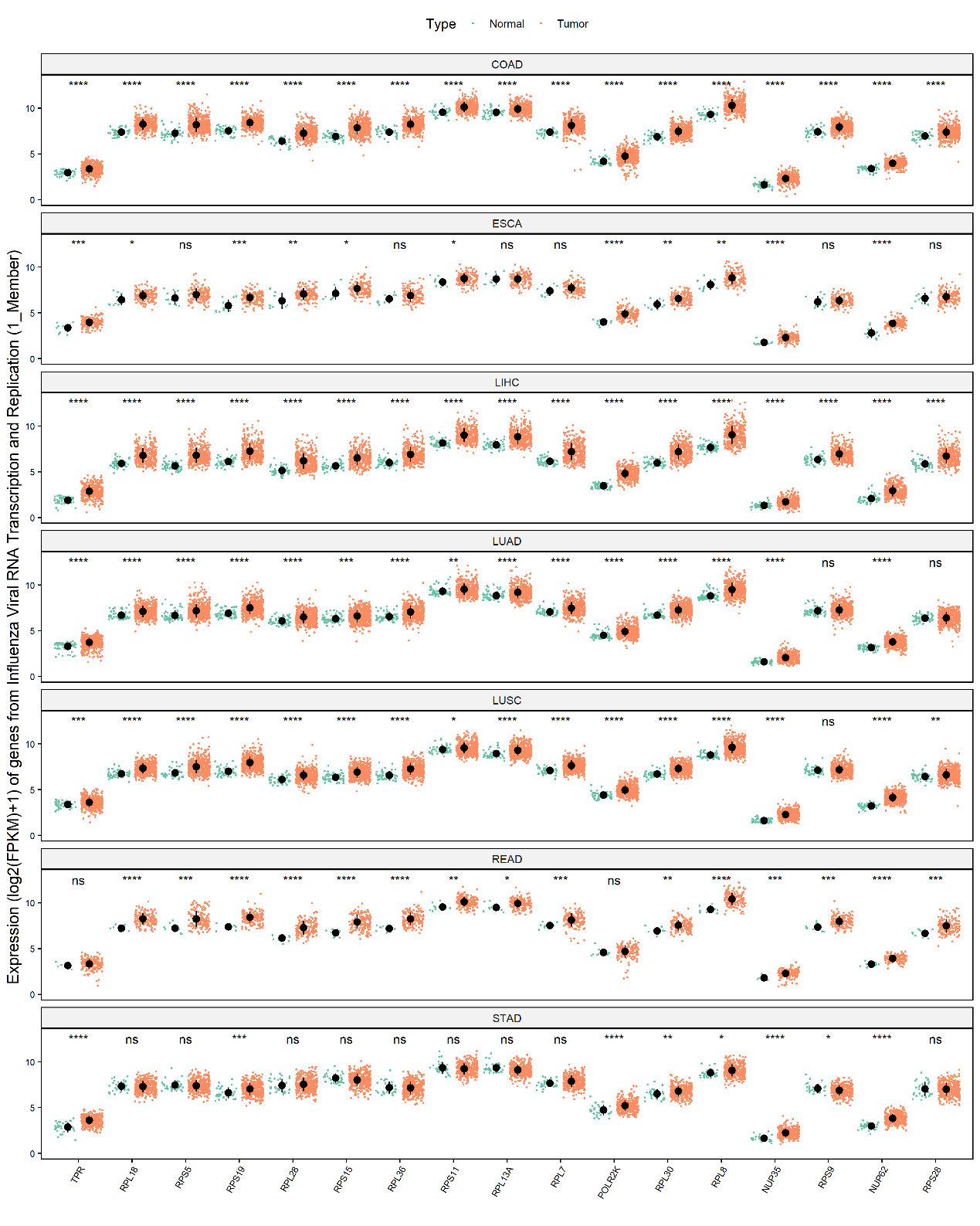


**Figure S14** (10) The expression profile of key genes in the key pathways


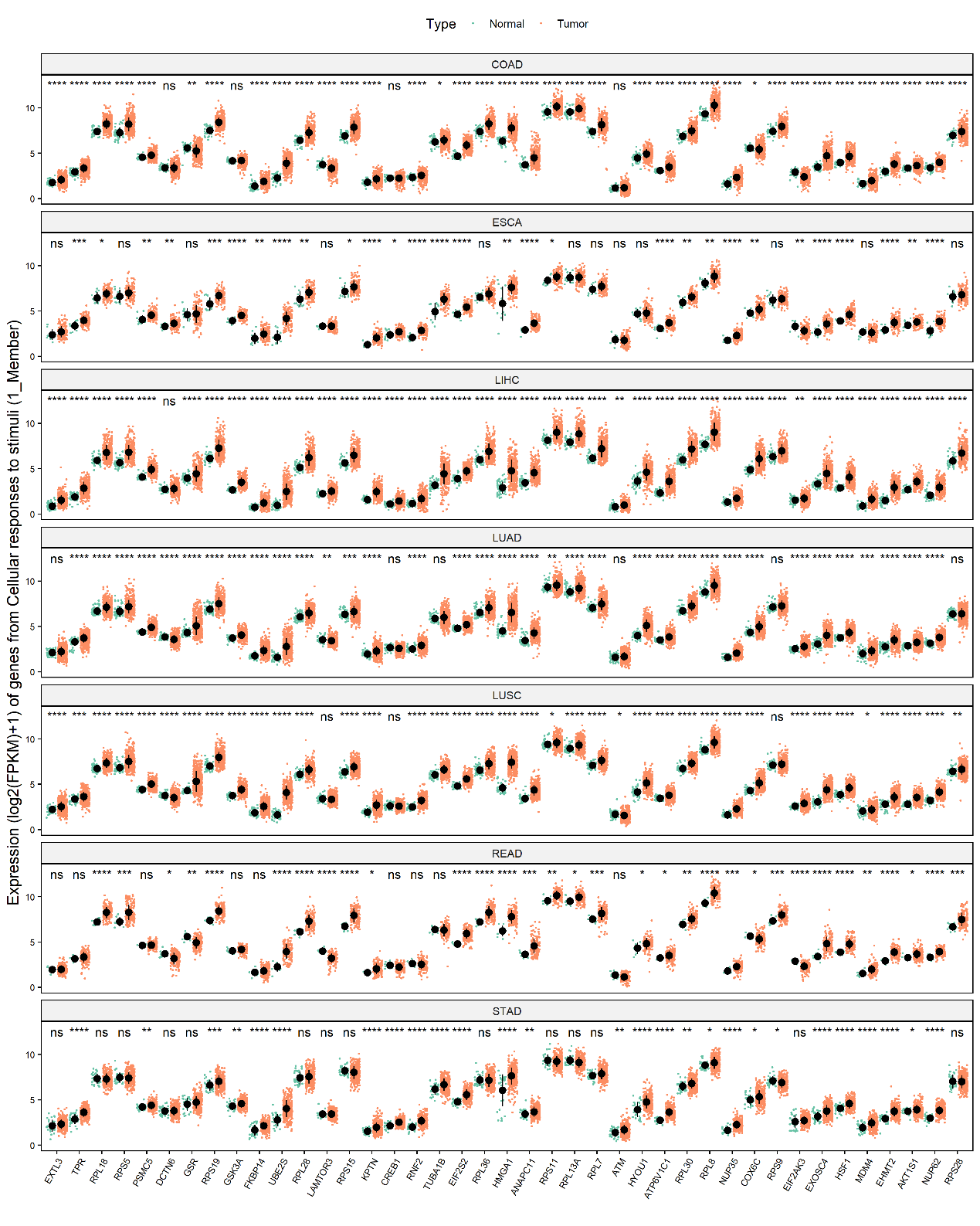


**Figure S14** (11) The expression profile of key genes in the key pathways


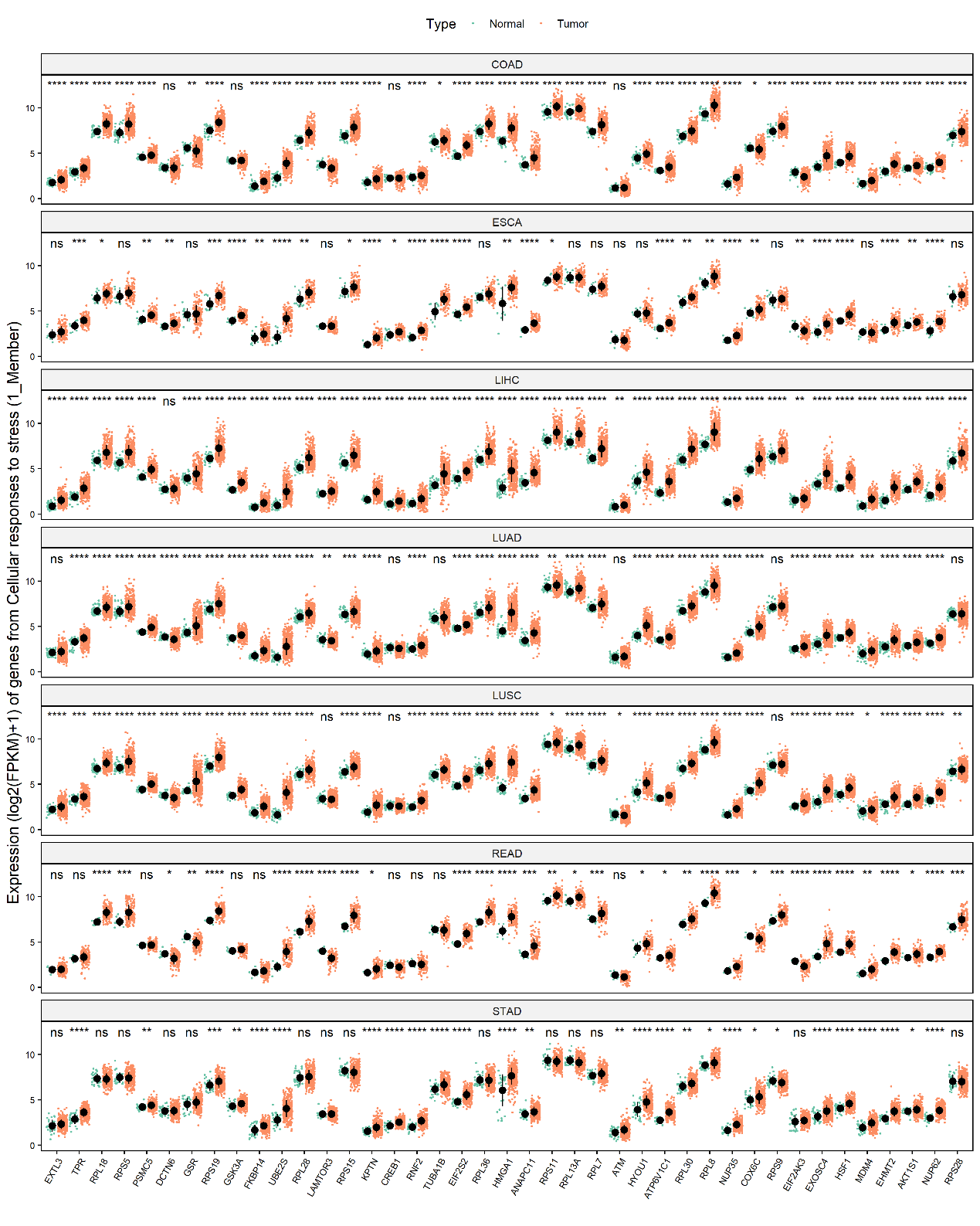


**Figure S14** (12) The expression profile of key genes in the key pathways


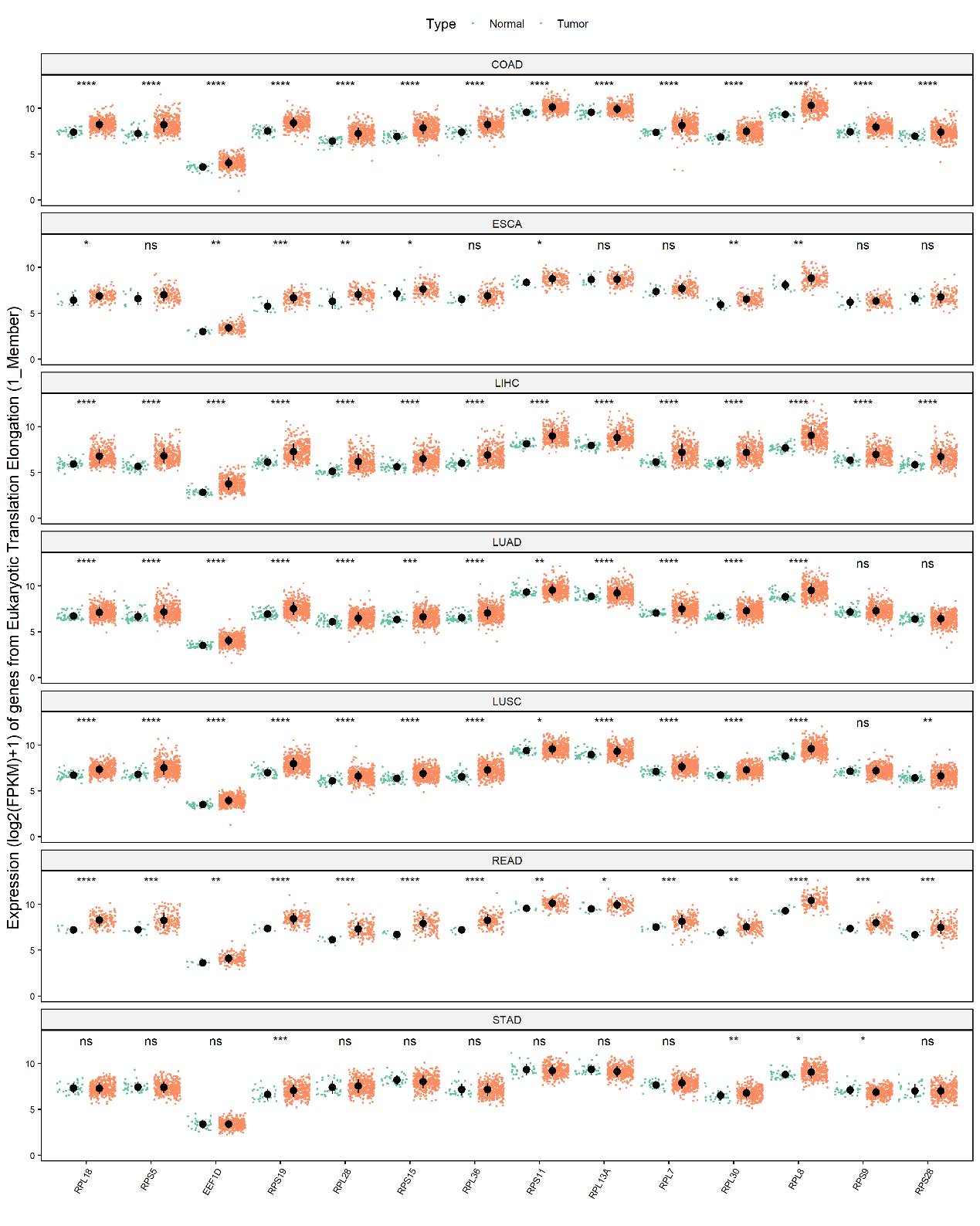


**Figure S14** (13) The expression profile of key genes in the key pathways


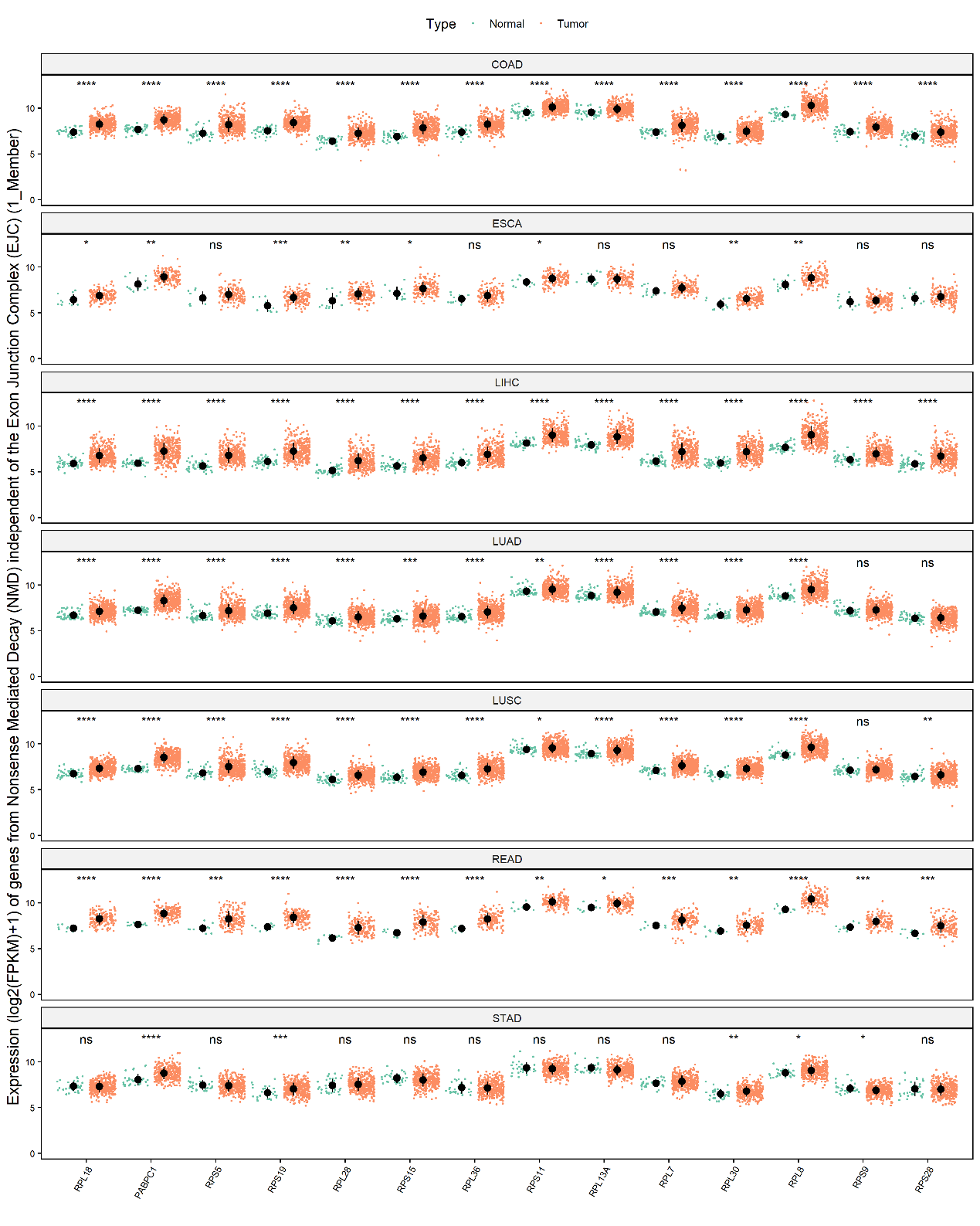


**Figure S14** (14) The expression profile of key genes in the key pathways


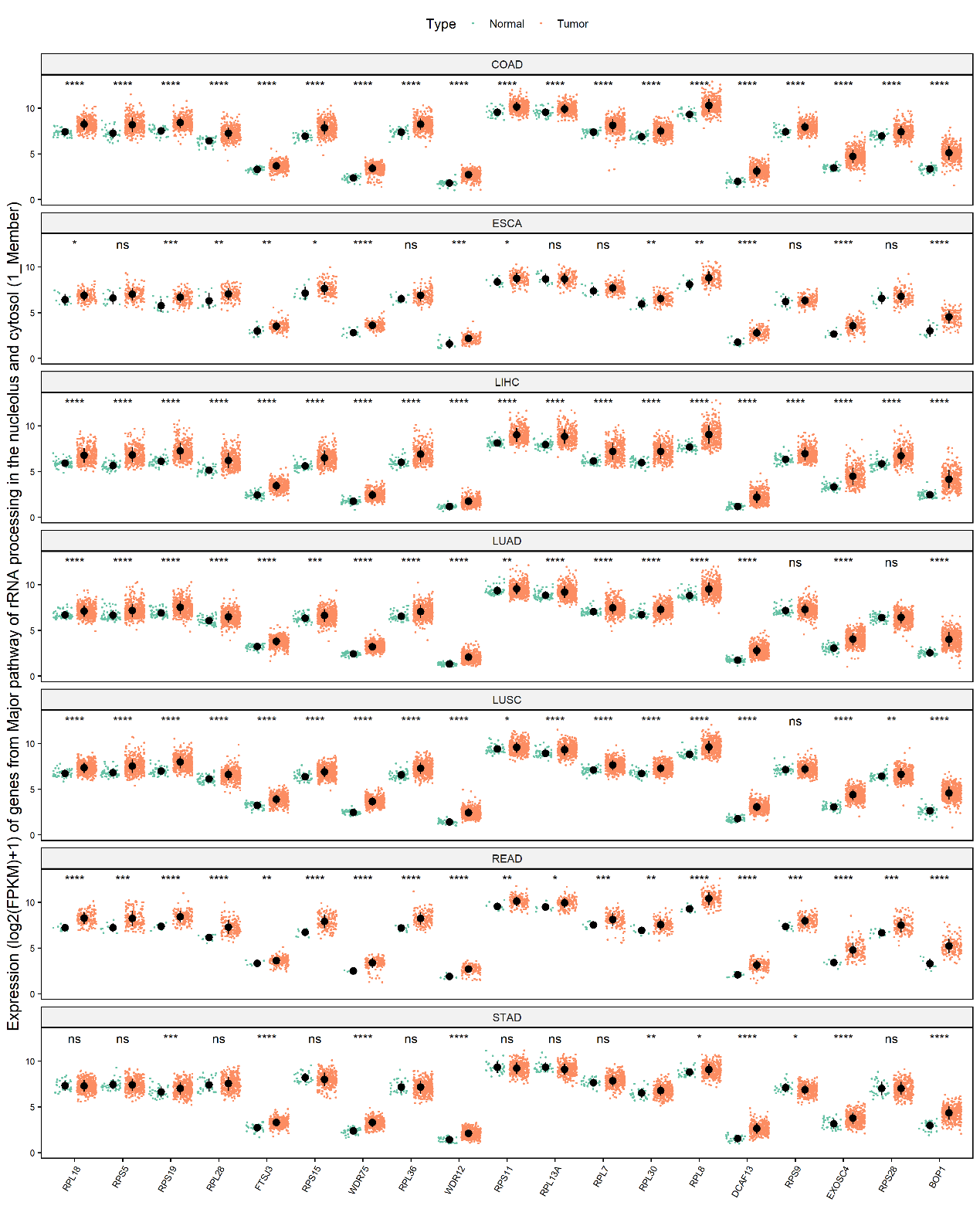


**Figure S14** (15) The expression profile of key genes in the key pathways


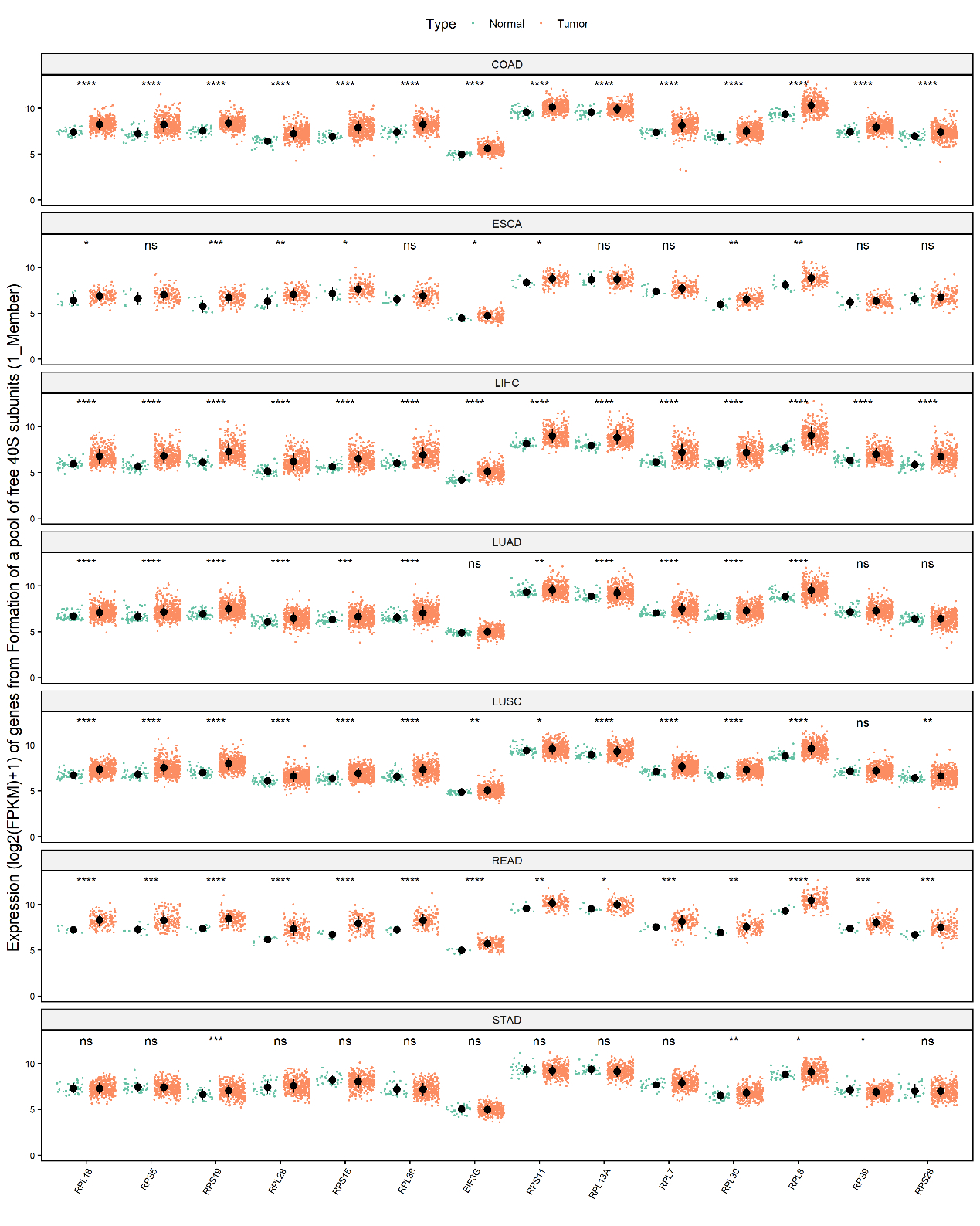


**Figure S14** (16) The expression profile of key genes in the key pathways


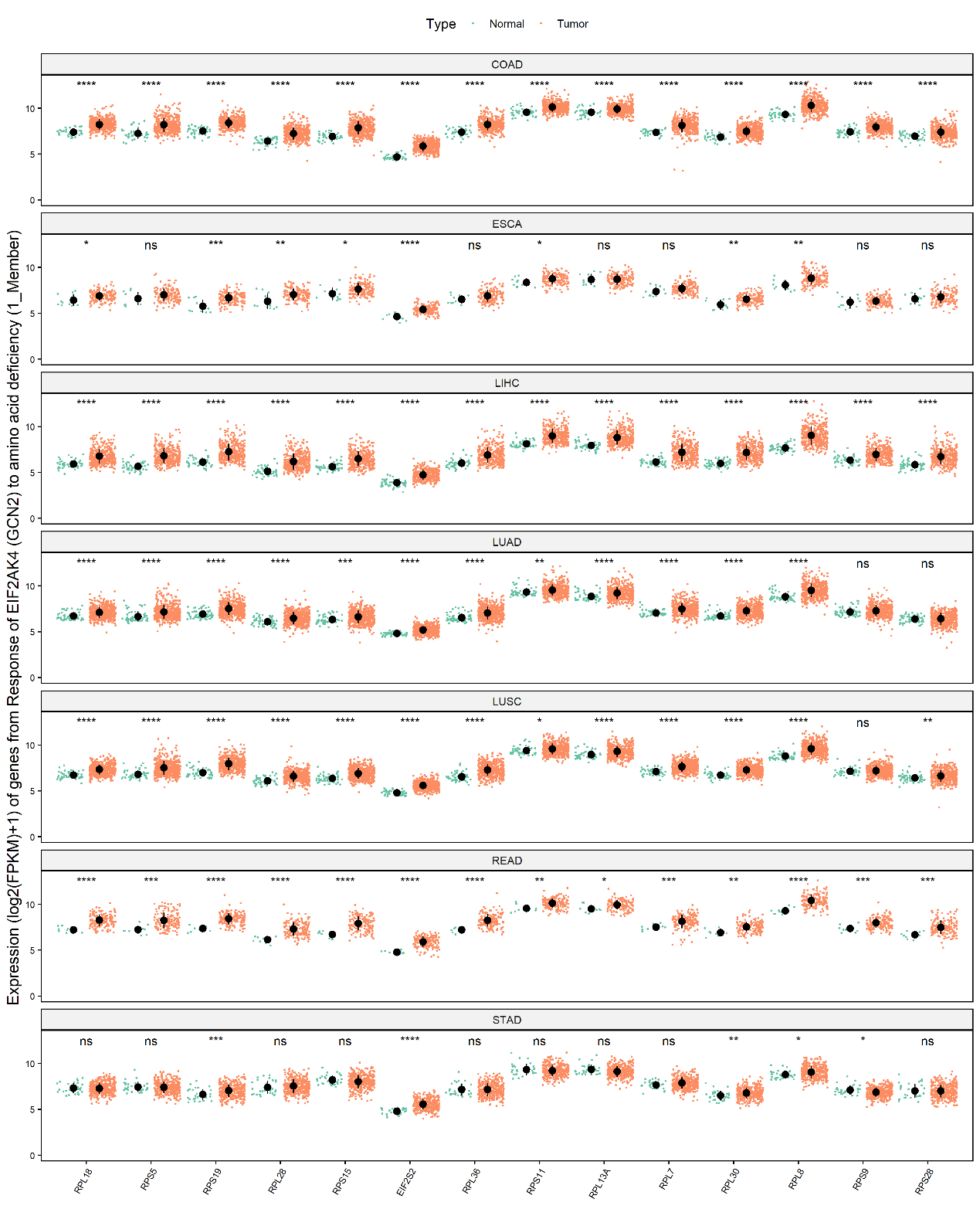


**Figure S14** (17) The expression profile of key genes in the key pathways


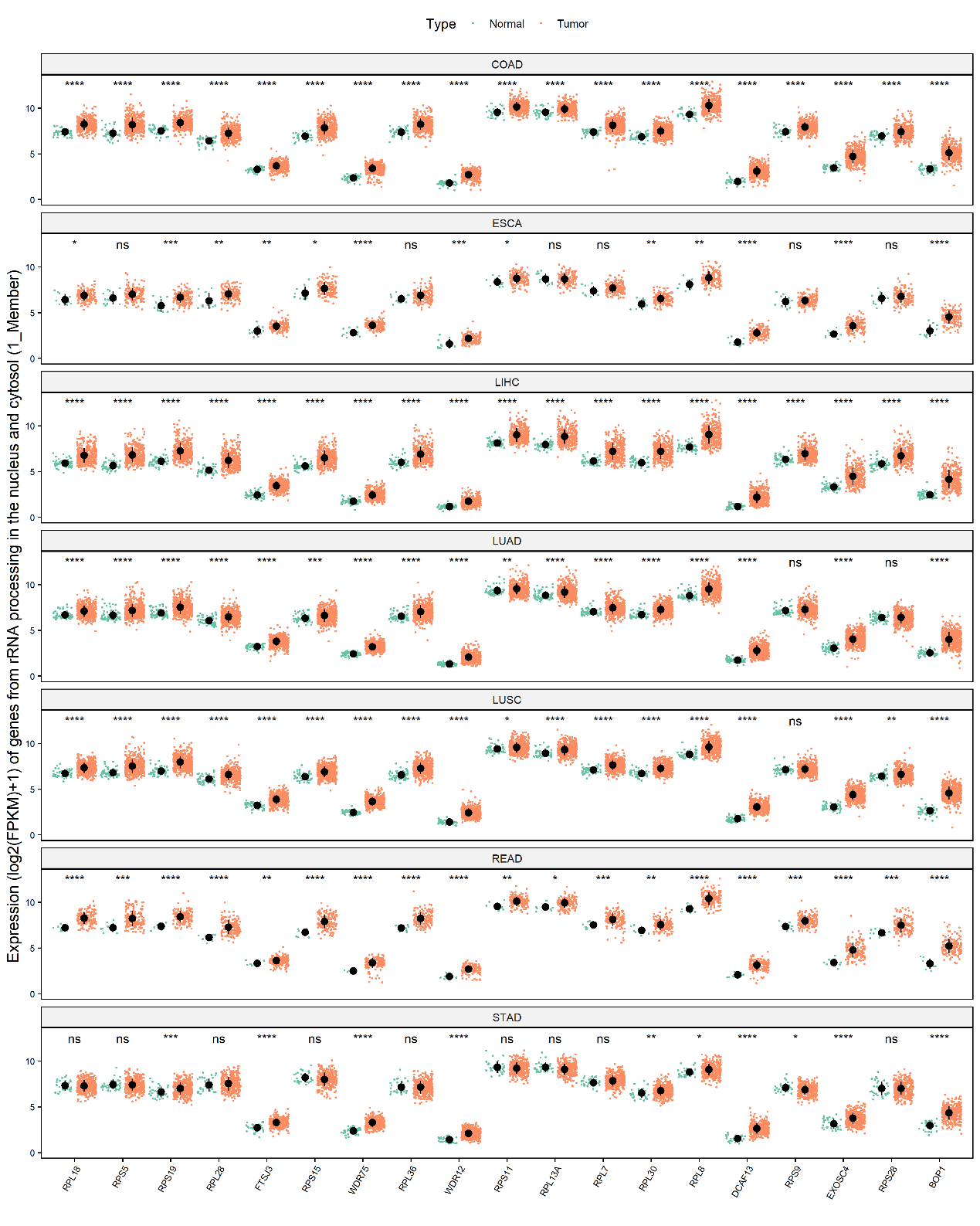


**Figure S14** (18) The expression profile of key genes in the key pathways


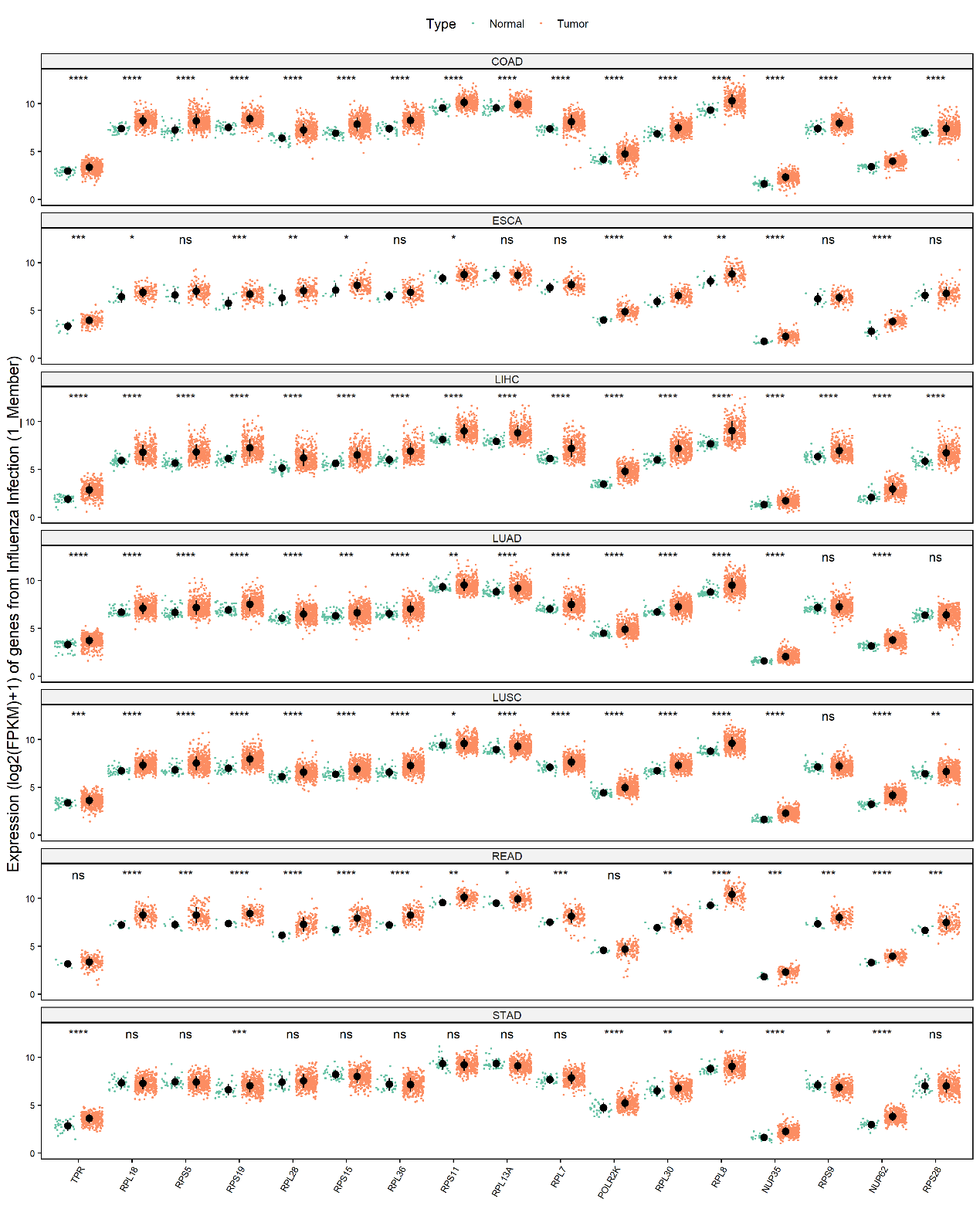


**Figure S14** (19) The expression profile of key genes in the key pathways
